# Identification of Differentially Expressed and Prognostic lncRNAs for the Construction of ceRNA Networks in Lung Adenocarcinoma

**DOI:** 10.1155/2021/2659550

**Published:** 2021-12-27

**Authors:** Yimeng Cui, Yaowen Cui, Ruixue Gu, Yuechao Liu, Xin Wang, Lulu Bi, Shuai Zhang, Weina Fan, Fanglin Tian, Yuning Zhan, Ningzhi Zhang, Ying Xing, Li Cai

**Affiliations:** The Fourth Department of Medical Oncology, Harbin Medical University Cancer Hospital, 150 Haping Road, Harbin 150040, China

## Abstract

**Background:**

Long noncoding RNAs (lncRNAs) could function as competitive endogenous RNAs (ceRNAs) to competitively adsorb microRNAs (miRNAs), thereby regulating the expression of their target protein-coding mRNAs. In this study, we aim to identify more effective diagnostic and prognostic markers for lung adenocarcinoma (LUAD).

**Methods:**

We obtained differentially expressed lncRNAs (DElncRNAs), miRNAs (DEmiRNAs), and mRNAs (DEmRNAs) for LUAD by using The Cancer Genomes Atlas (TCGA) portal. Weighted gene coexpression network analysis (WGCNA) was performed to unveil core gene modules associated with LUAD. The Cox proportional hazards model was performed to determine the prognostic significance of DElncRNAs. The diagnostic and prognostic significance of DElncRNAs was further verified based on the receiver operating characteristic curve (ROC). Cytoscape was used to construct the ceRNA networks comprising the lncRNAs-miRNAs-mRNAs axis based on the correlation obtained from the miRcode, miRDB, and TargetScan.

**Results:**

Compared with normal lung tissues, 2355 DElncRNAs, 820 DEmiRNAs, and 17289 DEmRNAs were identified in LUAD tissues. We generated 8 WGCNA core modules in the lncRNAs coexpression network, 5 modules in the miRNAs, and 12 modules in the mRNAs coexpression network, respectively. One lncRNA module (blue) consisting of 441 lncRNAs, two miRNA modules (blue and turquoise) containing 563 miRNAs, and one mRNA module (turquoise), which consisted of 15162 mRNAs, were mostly significantly related to LUAD status. Furthermore, 67 DEmRNAs were found to be tumor-associated as well as the target genes of the DElncRNAs-DEmiRNAs axis. Survival analyses showed that 6 lncRNAs (LINC01447, WWC2-AS2, OGFRP1, LINC00942, LINC01168, and AC005863.1) were significantly correlated with the prognosis of LUAD patients. Ultimately, the potential ceRNA networks including 6 DElncRNAs, 4 DEmiRNAs, and 22 DEmRNAs were constructed.

**Conclusion:**

Our study indicated that 6 DElncRNAs had the possibilities as diagnostic and prognostic biomarkers for LUAD. The lncRNA-mediated ceRNA networks might provide novel insights into the molecular mechanisms of LUAD progression.

## 1. Introduction

Lung cancer is the leading cause of cancer-related death worldwide, of which lung adenocarcinoma (LUAD) is the dominant histological subtype, accounting for 40% of all cases [1, 2]. Statistics show that a dismal 5-year survival rate is less than 20% despite recent advances in therapies [3]. The major factors in unfavorable prognosis of LUAD are diagnosis at terminal cancer and the propensity for metastasis [4]. Hence, there is an urgent need to identify new biomarkers to predict diagnosis and prognosis at an early stage and explore novel therapeutic targets for LUAD [5].

High-throughput genome sequencing and microarrays have indicated that 75% of the human genomes are transcribed into noncoding RNAs with the exception of protein-coding genes [6, 7]. Long noncoding RNAs (LncRNAs) are a class of RNA transcripts with a length of more than 200 nucleotides without protein-coding ability [8]. LncRNAs are broadly perceived for their functions in regulating biological processes through different mechanisms in various cancer types and have held substantial promise as novel biomarkers for cancer therapy [9–11]. MicroRNAs (miRNAs) have also been confirmed to play an important role in cancer progression over the past decades [12, 13]. Intriguingly, increasing evidence supports that lncRNAs act as endogenous molecular sponges that recognize and competitively bind to miRNAs by sharing miRNA response elements (MREs), indirectly regulating target mRNAs at a post-transcriptional level [14, 15]. Besides, the hypothesis that the complicated ceRNA networks participate in tumor development has been verified [16, 17]. For instance, the lncRNA ITGB8-AS1-miR-33b-5p-ITGA3 axis was reported to promote invasion and migration in colorectal cancer [18]. LncRNA PVT1, as a ceRNA for miR-143, upregulated HK2 expression and promoted proliferation of gallbladder cancer cells [19].

Weighted gene coexpression network analysis (WGCNA) lied in the construction of scale-free gene coexpression networks to identify crucial modules of highly correlated genes that are associated with specific clinical features [20, 21]. The advantage of WGCNA is that it can identify and cluster highly correlated genes into the same module. At present, WGCNA plays a significant role in multiple fields, such as cancer, nervous system, and genetic data analysis, which is extremely useful for identifying potential candidate biomarkers or novel treatment targets [22–25].

In the current study, we identified differently expressed lncRNAs (DElncRNAs), miRNAs (DEmiRNAs), and mRNAs (DEmRNAs) and obtained the key modules relevant to LUAD traits by using WGCNA. Six diagnostic and prognostic DElncRNAs and 6 lncRNAs-4 miRNAs-22 mRNAs ceRNA networks may provide a useful basis for formulating early diagnosis and individualized treatments in LUAD.

## 2. Methods

### 2.1. Research Process Design

**Figure 1:**
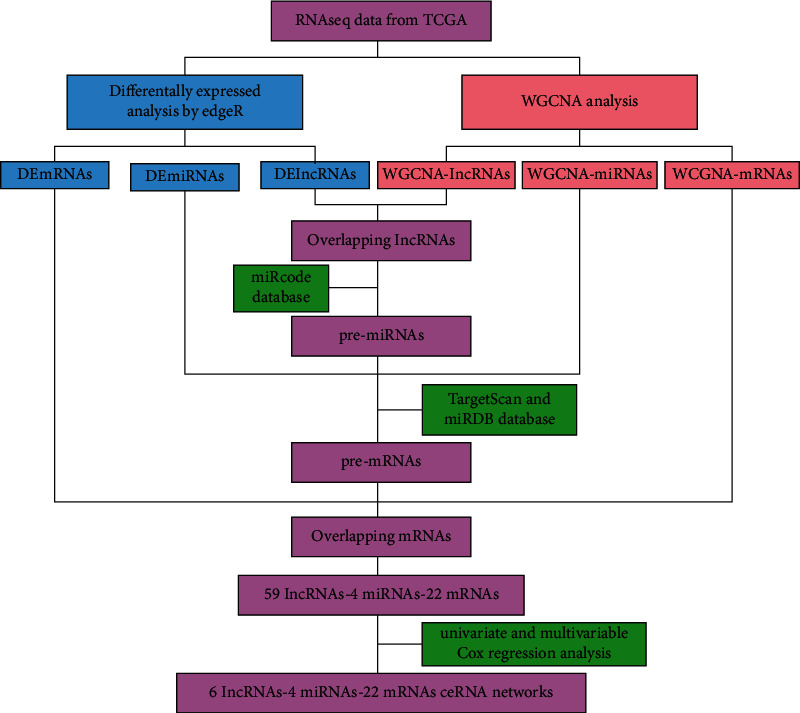
Research diagram of the ceRNA networks in LUAD.

The bioinformatics scheme design of the study is shown in Figure 1.

### 2.2. Data Collection and Processing

The transcriptome profiling data and clinical data of patients with LUAD (tumor = 534; normal = 59) were obtained from the TCGA database (https://portal.gdc.cancer.gov/) (Supplementary Table S3). LncRNA-seq data were extracted by comparing lncRNA annotation according to Genecode (https://www.gencodegenes.org/). We performed data analysis based on ‘Level 3' read count. TMM (trimmed mean of *M* value) normalization and differential expression analysis were implemented with the *R* package edgeR (|logFC| > 1.5 and *p* value < 0.05). Volcano maps were created using ggplot2 on Sangerbox (https://sangerbox.com/). The Venn diagram was performed using the Venny website (https://bioinfogp.cnb.csic.es/tools/venny/index.html).

### 2.3. Construction of the Weighted Gene Coexpression Network and Identification of Module Eigengenes

We incorporated RPKM (Reads Per Kilobase per Million) files of lncRNAs, miRNAs, and mRNAs into WGCNA analysis and constructed gene coexpression networks using the WGCNA *R* package [26]. The process included the following key steps [20, 21]: Firstly, the outliers were removed using the abline function for the clustered samples. Secondly, the established similarity matrix was converted into an adjacency matrix based on the *β* value. On this foundation, a topological overlap matrix (TOM) was constructed which was used to carry out the corresponding dissimilarity, and the hierarchical clustering tree of genes (dendrogram) was generated through hierarchical clustering to implement module detection. Finally, Module Members (MMs) and Gene Significance (GS) were counted and further investigated for module signature genes that were closely associated with cancer progression.

The construction process among lncRNA, miRNA, and mRNA coexpression networks was similar with the exception of some parameters: in the selection of soft power values, *β* values of lncRNAs, miRNAs, and mRNAs were 4, 3, and 1, respectively. The height cutoff MEDissThres of lncRNA, miRNA, and mRNA settings of similar modules was 0.5, 0.8, and 0.4, respectively. In terms of recognizing dynamic modules, 3 kinds of RNAs had the same conditions (deepSplit = 2, minModuleSize = 30).

### 2.4. Prediction of lncRNAs-miRNAs-mRNAs Networks

Forecasting target genes for lncRNAs and miRNAs through website tools: first of all, the overlapping lncRNA-targeted miRNAs (pre-miRNAs) were predicted via the miRcode website (https://www.mircode.org/) from which we obtained miRNA response element (MRE) information. The mRNAs (pre-mRNAs) targeted by shared miRNAs were predicted by TargetScan (https://www.targetscan.org/vert_72/) and miRDB databases (https://mirdb.org/). Genes with the same targeting relationship were extracted to construct the lncRNAs-miRNAs-mRNAs ceRNA networks using Cytoscape for visualization.

### 2.5. Survival Analysis

In combination with clinical information of TCGA-LUAD samples, univariate and multivariate Cox regression analysis were performed using survival *R* package Coxph function to clarify the relationship between characteristic lncRNAs and overall survival (OS), and forest maps were drawn using forestplot *R* package for visualization. LncRNAs significantly associated with prognosis were involved in the construction of the ceRNA regulatory networks. The area under the curve (AUC) for 1-year, 3-year, and 5-year OS was calculated by the ‘timeROC' *R* package to assess the predictive accuracy of prognosis. In addition, diagnostic ROC curves were plotted with IBM SPSS Statistics 26 for the lncRNA signature. *P* < 0.05 was considered statistically significant.

## 3. Results

### 3.1. Identification of DElncRNAs, DEmiRNAs, and DEmRNAs in LUAD

**Figure 2:**
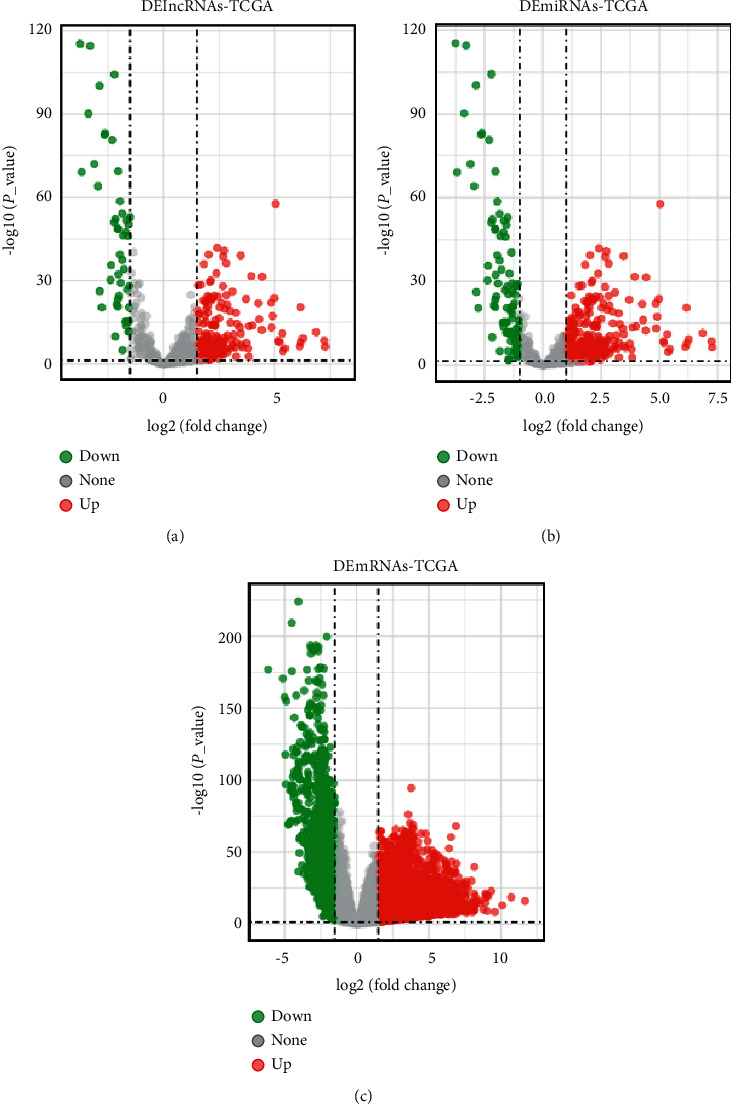
Identification of DElncRNAs, DEmiRNAs, and DEmRNAs in TCGA-LUAD. (a) Volcano plot of DElncRNAs from the TCGA database. (b) Volcano plot of DEmiRNAs from the TCGA database. (c) Volcano plot of DEmRNAs from the TCGA database. The *x*-axis and *y*-axis stood for log2 (fold change) of gene expression and lg-transformed *p* value, respectively. Red dots: the significantly overexpressed genes, green dots: downregulated genes, and gray dots: not significantly differentially expressed genes. |log2FC| > 1.5 and *p* < 0.05 were the cutoff criteria. Volcano maps were created using ggplot2 on the Sangerbox website (https://www.sangerbox.com/tool).

TCGA-LUAD mRNA expression data, including 534 LUAD samples and 59 normal samples, were downloaded and matched with Genecode v38 for obtaining lncRNA expression data. The expression profiles of miRNAs in 521 tumor samples and 46 normal samples were explored. Original count data were standardized, and differential expression analysis was implemented with the *R* package edgeR. In total, 641 DElncRNAs, 224 DEmiRNAs, and 5000 DEmRNAs were screened out (|logFC| > 1.5 and *p* < 0.05) (Supplementary Table S1). Volcano plots presented that 109 lncRNAs were downregulated, 48 miRNAs and 536 mRNAs were downregulated, and 532 lncRNAs, 176 miRNAs, and 4464 mRNAs were upregulated in LUAD samples (Figures 2(a)–2(c)).

### 3.2. Construction of Gene Coexpression Networks to Obtain Hub Modules

**Figure 3:**
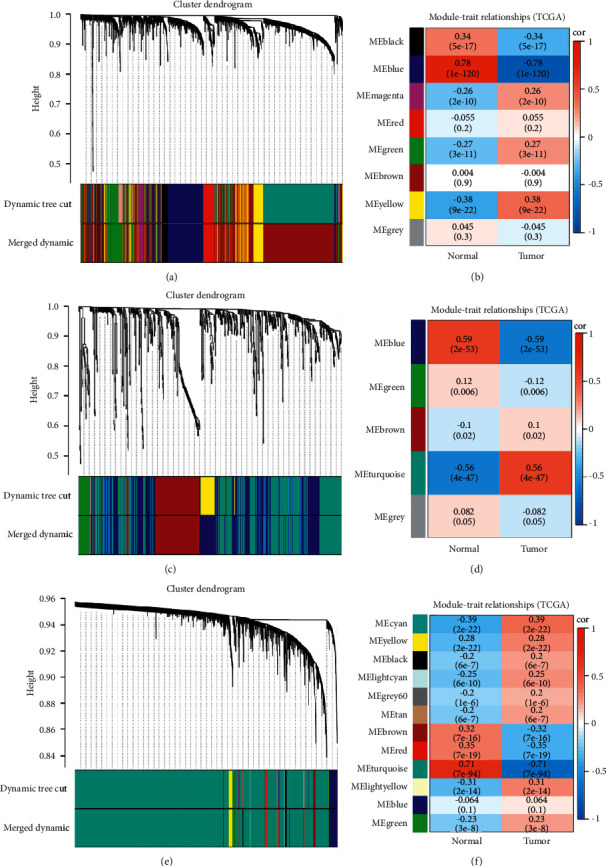
Drawing hierarchical clustering dendrograms of identified coexpressed genes and assessing the associations between module traits and the LUAD phenotype. Clustering dendrograms of lncRNAs (a), miRNAs (b), and mRNAs (c). *Note*. Each short vertical line corresponded to a gene and an expression module of genes that was highly interconnected (labeled on each branch). Two coloured rows below the dendrograms separately represented the original modules and merged modules. Analysis of module-trait relationships of LUAD based on lncRNA data (d), miRNA data (e), and mRNA data (f). *Note*. Each row corresponded to a module eigengene, and each column corresponded to a trait. Each cell contained the corresponding correlation (first line) and *p* value (second line). Color coding the table was according to the correlation of the color legend. *P* value < 0.05 represented statistical significance.

WGCNA, a systematic biological approach, was conducted to certify clinical phenotype in relation to coexpressed genes in networks. Selection of soft threshold power was a critical step in constructing WGCNA. To determine the relative balance between scale independence and average connectivity, we analyzed network topologies with soft threshold power ranging from 1 to 20. When the power value (*β*) was confirmed to 4 (lncRNAs), 3 (miRNAs), and 1 (mRNAs), the corresponding fitting index reached 0.9, and the coexpression network satisfied the scale-free distribution (Supplementary Figures S1(a)-S1(c)). We generated 8, 5, and 12 key modules (noted by different colors) in lncRNA, miRNA, and mRNA coexpression networks through the dynamic tree cutting method (Figures 3(a)–3(f)). Each module was color coded, but the genes in the gray module did not belong to any other module. Notably, we also identified the relationship of each module with the LUAD phenotype.

**Figure 4:**
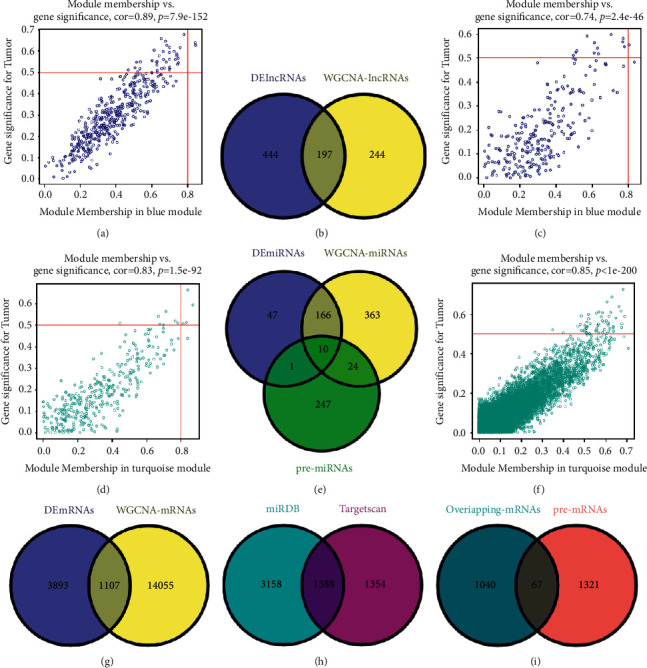
Scatter plots of gene significance (GS) and module membership (MM) in tumor-specific coexpression modules were displayed, and lncRNAs, miRNAs, and mRNAs were preliminarily screened for further analysis. Association between the vital modules included module eigengenes and tumor phenotypes in the coexpression network of (a) lncRNAs (weighted correlation of blue module characteristics = 0.89), (c, d) miRNAs (blue and turquoise modules trait weighted correlation = 0.74/0.83), and (f) mRNAs (turquoise module trait weighted correlation = 0.85). (b) The overlapping lncRNAs shared by DElncRNAs and WGCNA-lncRNAs. (e) The Venn diagram showed the intersection of DEmiRNAs, WGCNA-miRNAs, and pre-miRNAs (the target miRNAs of lncRNAs predicted by miRcode online prediction tools). (g) Venn diagram presented 1107 common mRNAs by intersecting DEmRNAs and WGCNA-mRNAs. (h) Based on the TargetScan and miRDB website, 1388 target genes of miRNAs were mostly overlapped. (i) The Venn diagram showed the unique correlation of genes among DEmRNAs, WGCNA-mRNAs, and pre-mRNAs.

The results showed that there was a significant association between the blue module and tumor phenotype in the lncRNAs coexpression networks (weighted correlation of module features = 0.78) (Figure 3(b)). Meanwhile, the turquoise module was obviously correlated with tumor characteristics in the mRNAs coexpression networks (module trait weighted correlation = 0.71) (Figure 3(f)). For miRNA coexpression networks, both blue and turquoise modules were significantly correlated with the tumor phenotype (module trait weighted correlation = 0.59/0.56) (Figure 3(d)). The genes in the core module were extracted for further analysis (WGCNA-lncRNAs = 441, WGCNA-miRNAs = 563, and WGCNA-mRNAs = 15162) (Figures 4(a), 4(c), 4(d), and 4(f)).

### 3.3. Prediction of lncRNAs-miRNAs and miRNAs-mRNAs Pairs

At first, we screened out 197 lncRNAs through matching the DElncRNAs with WGCNA-lncRNAs using the Venny website (Figure 4(b)). The predicted potential miRNAs (pre-miRNAs) that interacted with 197 lncRNAs were obtained using the miRcode database and identified a total of 7770 lncRNAs-miRNAs pairs, including 150 lncRNAs and 282 miRNAs. Taking the intersection of 24 DEmiRNAs, 282 pre-miRNAs, and 563 WGCNA-miRNAs, 10 miRNAs were ultimately included (Figure 4(e)). Then, 1107 mRNAs were selected by taking the intersection of 5000 DEmRNAs and 15162 WGCNA-mRNAs (Figure 4(g)). Next, 10 intersectional miRNAs were predicted by TargetScan and miRDB online target gene prediction tools for their target genes (Figure 4(e)). No targeted genes were predicted for miR-142-3p at the TargetScan website, and results of the remaining 9 miRNAs showed that 3074 miRNAs-mRNAs pairs included 2742 target genes; 6121 miRNAs-mRNAs pairs were retrieved on the miRDB website, containing 2742 target genes. There were 1388 mRNAs that were duplicated in both sites (Figure 4(h)). Finally, 67 target mRNAs were selected from DEmRNAs, mRNAs in the WGCNA core module, and predicted mRNAs (pre-mRNAs) (Figures 4(g) and 4(i)) (Supplementary Table S2). We performed reverse inferences based on 67 target genes and received 38 pairs of miRNAs-mRNAs (including 6 miRNAs and 38 mRNAs). The interaction effect between 6 miRNAs and 99 lncRNAs was also concluded at length.

### 3.4. Construction of lncRNAs-miRNAs-mRNAs Networks for LUAD

**Figure 5:**
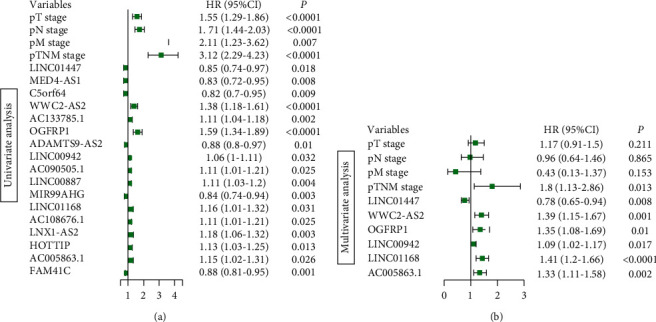
Univariate and multivariate Cox regression analysis. (a) The forest plot showed the prognostic factors associated with overall survival rates using univariate analysis. (b) Six lncRNAs were independent prognostic factors for patients with LUAD by performing multivariate Cox regression analysis. Hazard ratios (HRs) > 1 indicated a factor with poor prognosis, whereas HRs < 1 were related to favorable prognosis. All the variables shown were statistically significant with *p* value < 0.05.

**Figure 6:**
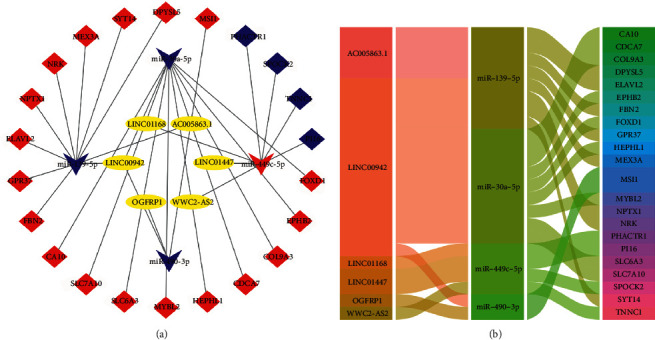
Visualization of ceRNAs networks. (a) Construction of 6 lncRNAs-4 miRNAs-22 mRNAs ceRNAs networks. *Note*. Diamonds denoted miRNAs, squares represented mRNAs, and yellow round rectangles represented lncRNAs. Red and blue indicated upregulated and downregulated genes in LUAD. (b) The alluvial plot of 6 lncRNAs-4 miRNAs-22 mRNAs ceRNA regulatory networks consisted of 3 columns (lncRNAs-miRNAs-mRNAs).

When miRNA binds to MRE on lncRNAs, mRNA expression is not inhibited; hence, miRNAs are mostly negatively correlated with lncRNA and mRNA expression (Supplementary Figure S2) [27]. Therefore, we screened for negatively associated genes, which included 59 lncRNAs, 4 miRNAs, and 22 mRNAs. Clinical data were downloaded from TCGA-LUAD, of which 512 samples had complete clinical information. Clinicopathological features of pT stage, pN stage, pM stage, and pTNM stage were incorporated into analysis, and the Coxph function in the survival *R* package was used to perform univariate and multivariate Cox regression analysis (Supplementary Tables S4 and S5.). As a consequence, 6 lncRNAs were identified as crucial prognostic factors (WWC2-AS2, OGFRP1, LINC00942, LINC01168, and AC005863.1 were risk factors, and only LINC01447 belonged to protective factor) (Figures 5(a) and 5(b)) (Supplementary Table S6). Cox regression analysis was performed to obtain risk scores of each sample which were used for ROC analysis of the prognosis classification utilizing the ‘timeROC' *R* package. As shown in Supplementary Figure S3(a), the lncRNA signature is an independent predictor which reached an optimism-corrected AUC of 0.79 (1 year), 0.79 (3 years), and 0.77 (5 years). Meanwhile, diagnostic ROC curves further demonstrated the superior clinical utility of the prognostic lncRNA model (AUC = 0.728) (Supplementary Figure S3(b)). Eventually, we constructed ceRNA networks for 6 lncRNAs, 4 miRNAs, and 22 mRNAs, that were visualized using Cytoscape v3.7.2 software and an alluvial plot (Figures 6(a) and 6(b)).

## 4. Discussion

Due to the unfavorable prognosis and high mortality rate of LUAD, it is necessary to improve the strategy of diagnosis and treatment. The lncRNA-mediated ceRNA hypothesis proposed that lncRNA functions as a ceRNA to regulate the gene expression by influencing miRNA activity. A previous study suggested that lncRNA-KRTAP5-AS1 and lncRNA-TUBB2A could serve as ceRNA to reinforce proliferation, invasion, and EMT function of Claudin-4 [28]. HOXD-AS1 was bound to miR-130a-3p in a competitive manner, which activated the expression of EZH2 and MMP2 and facilitated liver cancer metastasis [29]. Previous studies suggested that lncRNA as ceRNA played an important biological function in LUAD, but the tumor-specific ceRNA networks launched by lncRNAs remained largely unknown [30, 31]. Different from lncRNA-regulated ceRNA networks in LUAD established by Wu et al., six distinct lncRNAs were exhibited in our ceRNA networks. The reason may be that different bioinformatics tools and concerns were applied (i.e., we used the WGCNA analysis and conducted Cox regression analysis to identify cancer-related prognostic lncRNAs).

In the present study, we identified 6 differentially expressed and prognostic lncRNAs. Among them, LINC01447, LINC01168, and AC005863.1 have not been reported to date. It is interesting to explore the biofunctional role in the development and progression of LUAD for these three lncRNAs. The other three were lncRNA OGFRP1, LINC00942, and WWC2-AS2, which were both reported in the field of malignant tumors [32–36]. OGFRP1 promoted tumor progression by increasing the activity of the AKT/mTOR pathway or directly interacting with miR-4640-5p [32, 33]. Recent studies have shown that LINC00942 potentiated breast cancer cell proliferation and progression by affecting METTL14-mediated m6A methylation [34]. WWC2-AS2 and LINC00942 were involved in the construction of a prognostic lncRNA signature in cervical cancer and lung adenocarcinoma [35, 36]. In the current study, high expression of OGFRP1, LINC00942, and WWC2-AS2 was associated with poor prognosis of LUAD patients, which was in line with the abovereported results. Nevertheless, all these lncRNAs associated with molecular events still need further experimental validation in LUAD.

Four predicted miRNAs in ceRNA networks stand out in our study. These DEmiRNAs are as follows: miR-139-5p (downregulated), miR-30a-5p (downregulated), miR-490-3p (downregulated), and miR-449c-5p (upregulated). Consistently, miR-139-5p was downregulated in LUAD and exerted the ability to inhibit proliferation, migration, and invasion of cancer cells by targeting MAD2L1 [37]. Moreover, several studies have found that miR-30a-5p inhibited the proliferation of multiple cancers, such as breast cancer, glioma, and lung squamous cell carcinoma [38–40]. It is reported that miR-490-3p overexpression significantly inhibited the proliferation, invasion, and migration of hepatocellular carcinoma cells by activating BCYRN1 [41]. MiR-449c-5p was a hub for circ-NOTCH1 to promote metastasis and stemness of gastric cancer cells, leading to the disease progression of gastric cancer [42]. These DEmiRNAs might serve as putative targets for LUAD diagnosis and therapy.

In the established ceRNA networks, the 22 DEmRNAs attracted the researchers' attention, and they found that they were effective regulators during cancer progression [43–49]. EPHB2 has been associated with cancer stemness and acquired sorafenib resistance via the *β*-catenin/TCF1 axis [43]. CXCL5 as a tumor angiogenic factor promoted the expression of FOXD1 by activating the AKT/NF-*κ*B pathway in colorectal cancer [44]. High expression of CDCA7 promoted tumorigenesis and predicted poorer prognosis in patients with TNBC and ESCC [45, 46]. Downregulation of TNNC1 (Troponin C1) expression accelerated tumor formation and increased mortality in LUAD patients [47]. Glioma cells with low SYT14 (Synaptotagmin 14) expression were observed to suppress the proliferation capacity [48]. Upregulation of SPOCK2 negatively regulated MMP2 gene expression, which in turn inhibited the invasion and metastasis of prostate cancer cells [49]. These studies indicated these potent cancer regulators involved in the present ceRNA networks.

## 5. Conclusions

We used bioinformatics methods to construct the LUAD-specific lncRNA-mediated ceRNA regulatory networks. We also identified 6 DElncRNAs as prognostic biomarkers which might play critical roles in tumorigenesis and development of lung cancer. Further experimental verification is needed to elucidate the underlying regulatory mechanism in the future.

## Data Availability

All data generated or analyzed during this study are included in this published article and its supplementary information files.

## Supplementary Materials

Supplementary Materials Supplementary Figure S1. Analysis of network topology for soft thresholding powers (weighting coefficient, *β*) in lncRNAs, miRNAs, and mRNAs. The *x*-axis represented different soft thresholding powers. Upper: assessment for *R*^2^ of log(*k*) and log(*p*(*k*)) correlation coefficients corresponding to different *β* values in the network. The red line indicated a scale-free topology fitting index *R*^2^ of 0.9. Lower: analysis of the mean connectivity for various *β* values. Supplementary Figure S2. Correlation analysis of lncRNAs, miRNAs, and mRNAs in the ceRNA network. (a) LncRNAs were negatively correlated with miRNAs. (b) Negative correlation among mRNAs and miRNAs. (c) LncRNAs had a positive correlation with mRNAs. Supplementary Figure S3. ROC plots of the prognostic lncRNA signature in the TCGA-LUAD dataset. (a) Survival-dependent ROC curves attested the prognostic significance of DElncRNAs. The area under the red line represented the 1-year AUC; the area under the blue line represented the 3-year AUC; and the area under the black line represented the 5-year AUC. (b) ROC curve analysis showed the application value of DElncRNAs in the diagnosis. Supplementary Table S1. The summarized data of DERNAs. Supplementary Table S2. The integrated results of DERNAs via differential expression analysis, WGCNA, and website prediction. Supplementary Table S3. Baseline clinicopathological characteristics of the TCGA-LUAD cohort. (*n* = 513). Supplementary Table S4. Univariate Cox regression analysis of factors associated with overall survival in the TCGA-LUAD dataset. Supplementary Table S5. Multivariable Cox regression analysis of factors associated with overall survival in the TCGA-LUAD dataset. Supplementary Table S6. 6 prognostic lncRNA-mediated ceRNA networks.
