# Supplementary material for: Identification of Differentially Expressed and Prognostic lncRNAs for the Construction of ceRNA Networks in Lung Adenocarcinoma
Source: J Oncol. 2021 Dec 27;2021:2659550. doi: 10.1155/2021/2659550 (PMC8723861; doi:10.1155/2021/2659550)
Supplement: Supplementary Materials — Supplementary Figure S1. Analysis of network topology for soft thresholding powers (weighting coefficient, β) in lncRNAs, miRNAs, and mRNAs. The x-axis represented different soft thresholding powers. Upper: assessment for R2 of log(k) and log(p(k)) correlation coefficients corresponding to different β values in the network. The red line indicated a scale-free topology fitting index R2 of 0.9. Lower: analysis of the mean connectivity for various β values. Supplementary Figure S2. Correlation analysis of lncRNAs, miRNAs, and mRNAs in the ceRNA network. (a) LncRNAs were negatively correlated with miRNAs. (b) Negative correlation among mRNAs and miRNAs. (c) LncRNAs had a positive correlation with mRNAs. Supplementary Figure S3. ROC plots of the prognostic lncRNA signature in the TCGA-LUAD dataset. (a) Survival-dependent ROC curves attested the prognostic significance of DElncRNAs. The area under the red line represented the 1-year AUC; the area under the blue line represented the 3-year AUC; and the area under the black line represented the 5-year AUC. (b) ROC curve analysis showed the application value of DElncRNAs in the diagnosis. Supplementary Table S1. The summarized data of DERNAs. Supplementary Table S2. The integrated results of DERNAs via differential expression analysis, WGCNA, and website prediction. Supplementary Table S3. Baseline clinicopathological characteristics of the TCGA-LUAD cohort. (n = 513). Supplementary Table S4. Univariate Cox regression analysis of factors associated with overall survival in the TCGA-LUAD dataset. Supplementary Table S5. Multivariable Cox regression analysis of factors associated with overall survival in the TCGA-LUAD dataset. Supplementary Table S6. 6 prognostic lncRNA-mediated ceRNA networks. [file 2659550.f1.zip › 2659550.f1/Supplementary Table S1..doc]

| Supplementary Table S1. The summarized data of DERNAs. | | |
| --- | --- | --- |
| DElncRNAs | DEmiRNAs | DEmRNAs |
| LANCL1-AS1 | hsa-miR-486-5p | TFF2 |
| FENDRR | hsa-miR-139-3p | REG4 |
| LINC00968 | hsa-let-7d-3p | LINC00676 |
| LINC01082 | hsa-miR-30c-2-3p | DEFA5 |
| HID1-AS1 | hsa-miR-144-5p | TFF1 |
| C14orf132 | hsa-miR-30a-3p | ALB |
| LINC00656 | hsa-miR-143-3p | RNU5B-1 |
| PCAT19 | hsa-miR-195-5p | FGB |
| AC104984.4 | hsa-miR-144-3p | SPINK4 |
| AC128709.3 | hsa-miR-197-3p | CGA |
| LINC00163 | hsa-miR-4732-3p | FGF19 |
| LINC01290 | hsa-miR-451a | MAGEA4 |
| AC010976.2 | hsa-miR-378a-3p | CALCA |
| MIR22HG | hsa-miR-210-3p | AFP |
| MBNL1-AS1 | hsa-miR-218-5p | TM4SF5 |
| ADAMTS9-AS1 | hsa-let-7b-3p | PDX1 |
| LINC00551 | hsa-miR-190a-5p | MAGEA3 |
| LINC00987 | hsa-miR-145-3p | HOXC12 |
| EP300-AS1 | hsa-miR-6892-5p | INSL4 |
| OR7E47P | hsa-miR-374a-5p | GC |
| LINC01352 | hsa-miR-328-3p | GCG |
| LINC01197 | hsa-miR-218-1-3p | PITX2 |
| LINC01447 | hsa-let-7a-5p | MAGEA6 |
| TBX2-AS1 | hsa-let-7c-5p | SERPINA4 |
| MED4-AS1 | hsa-miR-423-5p | PSG1 |
| FAM83A-AS1 | hsa-let-7g-3p | PRB4 |
| LINC00702 | hsa-miR-96-5p | MAGEC1 |
| LINC01506 | hsa-let-7f-5p | TRIM48 |
| PTPRD-AS1 | hsa-miR-21-3p | C11orf86 |
| LINC00511 | hsa-miR-1287-3p | MAGEA10 |
| PVT1 | hsa-miR-708-3p | ACTL8 |
| AFAP1-AS1 | hsa-miR-30a-5p | DSCAM-AS1 |
| AC011239.2 | hsa-miR-708-5p | MAGEC2 |
| MNX1-AS1 | hsa-miR-7-1-3p | CPN1 |
| LINC00891 | hsa-miR-1-3p | KRT20 |
| MAGI2-AS3 | hsa-miR-139-5p | DCAF4L2 |
| DDX11-AS1 | hsa-miR-183-5p | LIN28B |
| MGC27382 | hsa-miR-150-3p | PAGE2 |
| RUNDC3A-AS1 | hsa-miR-301a-5p | PAEP |
| ZFPM2-AS1 | hsa-miR-153-5p | MUC17 |
| LINC01572 | hsa-miR-133b | RN7SKP255 |
| PCAT6 | hsa-miR-671-5p | RNU5A-1 |
| LINC01270 | hsa-miR-182-5p | REG1A |
| FGF10-AS1 | hsa-miR-6511b-3p | PSG4 |
| TBX5-AS1 | hsa-miR-21-5p | UGT1A10 |
| AC004947.2 | hsa-miR-26b-3p | PRAME |
| FEZF1-AS1 | hsa-miR-378c | ANXA10 |
| AC144831.1 | hsa-miR-33a-5p | RNY1 |
| HLX-AS1 | hsa-miR-27a-5p | RNA5-8SP2 |
| FOXD3-AS1 | hsa-miR-184 | LINC02418 |
| LINC01165 | hsa-miR-33b-5p | BARX1 |
| LINC01070 | hsa-miR-135b-5p | COX7B2 |
| DGCR5 | hsa-miR-133a-3p | CALML5 |
| AC004490.1 | hsa-miR-1307-5p | H1-5 |
| NAV2-AS2 | hsa-miR-200a-5p | CGB5 |
| C5orf64 | hsa-miR-4668-3p | NKX1-2 |
| C1orf220 | hsa-miR-20a-3p | PSG3 |
| TFAP2A-AS1 | hsa-miR-143-5p | AKR1B10 |
| LINC00472 | hsa-miR-577 | SST |
| LINC00857 | hsa-miR-301a-3p | PSG5 |
| VPS9D1-AS1 | hsa-miR-409-5p | MAGEA12 |
| LINC00844 | hsa-miR-605-5p | SPP2 |
| WWC2-AS2 | hsa-miR-141-5p | UGT2B11 |
| LINC01271 | hsa-miR-142-3p | FGL1 |
| LINC00092 | hsa-miR-130b-5p | RNU4-1 |
| WASIR2 | hsa-miR-744-3p | MAGEA1 |
| SNHG1 | hsa-miR-9-5p | EPS8L3 |
| CADM3-AS1 | hsa-miR-503-5p | F2 |
| BLACAT1 | hsa-miR-147b-3p | LINC01419 |
| LINC00211 | hsa-miR-1247-3p | CSAG1 |
| AC133785.1 | hsa-miR-183-3p | HOXB9 |
| LINC01412 | hsa-miR-29b-1-5p | AC026785.3 |
| AC007128.1 | hsa-miR-486-3p | KRTAP4-1 |
| SNHG4 | hsa-miR-196a-5p | APOBEC1 |
| AGAP11 | hsa-miR-127-5p | MAGEA11 |
| LINC00896 | hsa-miR-2110 | 7SK |
| BBOX1-AS1 | hsa-miR-194-3p | DLK1 |
| LINC01426 | hsa-miR-345-5p | AC011298.1 |
| AC007743.1 | hsa-miR-135b-3p | AC079466.1 |
| AF131215.6 | hsa-miR-29a-5p | TRPM8 |
| FGF14-AS2 | hsa-miR-616-5p | AL160271.2 |
| FOXD2-AS1 | hsa-miR-200a-3p | TFAP2D |
| LINC00862 | hsa-miR-301b-3p | SNORA71D |
| SLC2A1-AS1 | hsa-miR-130b-3p | RNY3 |
| NPSR1-AS1 | hsa-miR-141-3p | VIL1 |
| TYMSOS | hsa-miR-450a-5p | HOXC13 |
| CLDN10-AS1 | hsa-miR-31-5p | MAGEA9B |
| SNHG3 | hsa-miR-3170 | RNU4-2 |
| PGM5-AS1 | hsa-miR-301b-5p | SNORA74A |
| ZNF252P-AS1 | hsa-miR-136-5p | GAGE2A |
| GHET1 | hsa-miR-6502-5p | SLCO1B3 |
| CYP4A22-AS1 | hsa-miR-629-3p | PADI3 |
| SLC22A18AS | hsa-miR-338-5p | CST4 |
| LINC01460 | hsa-miR-4521 | FOXD3-AS1 |
| OGFRP1 | hsa-miR-424-5p | UGT1A8 |
| LINC01012 | hsa-miR-155-3p | PSG11 |
| ADAMTS9-AS2 | hsa-miR-589-3p | H4C13 |
| LINC00942 | hsa-miR-539-5p | FAM83A |
| KCNMB2-AS1 | hsa-miR-224-5p | TPSP2 |
| HOXC-AS2 | hsa-miR-548v | POU3F2 |
| LINC01031 | hsa-miR-188-3p | LINC02882 |
| MGAT3-AS1 | hsa-let-7a-2-3p | HOXC11 |
| AC104024.2 | hsa-miR-9-3p | HOTAIR |
| TMEM51-AS1 | hsa-miR-504-5p | DLL3 |
| LINC00629 | hsa-miR-3136-5p | SNORA23 |
| RAMP2-AS1 | hsa-miR-31-3p | SPAG11B |
| LINC00628 | hsa-miR-4652-5p | SP8 |
| TMPO-AS1 | hsa-miR-196b-5p | TINAG |
| LINC01395 | hsa-miR-137-3p | CYP24A1 |
| AC005256.1 | hsa-miR-1258 | SNORA73B |
| AC090505.1 | hsa-miR-193b-3p | SCARNA5 |
| LINC00887 | hsa-miR-1247-5p | VGLL2 |
| UBE2Q1-AS1 | hsa-miR-105-5p | NEUROD1 |
| LINC00525 | hsa-miR-1269a | FOXI3 |
| LINC00624 | hsa-miR-323b-3p | LHX1 |
| LINC00880 | hsa-miR-103a-2-5p | H3C7 |
| AC092171.4 | hsa-miR-616-3p | TMPRSS11E |
| LINC00461 | hsa-miR-3189-3p | PCSK1 |
| LINC01415 | hsa-miR-192-5p | CPS1 |
| AC024560.2 | hsa-miR-1277-3p | LINC02864 |
| LINC01348 | hsa-miR-187-3p | KLK6 |
| PTPRG-AS1 | hsa-miR-2277-5p | TUBA3C |
| LINC00665 | hsa-miR-490-3p | AC005256.1 |
| C1orf147 | hsa-miR-579-5p | AC133785.1 |
| F11-AS1 | hsa-miR-377-3p | BPIFB4 |
| PGM5P4-AS1 | hsa-miR-20b-3p | SNORA49 |
| VAC14-AS1 | hsa-miR-767-5p | H1-3 |
| MNX1-AS2 | hsa-miR-4724-5p | SPINK1 |
| RMDN2-AS1 | hsa-miR-4638-3p | SPAG11A |
| LINC00337 | hsa-miR-592 | HOXD13 |
| RHPN1-AS1 | hsa-miR-215-5p | CT83 |
| PRRT3-AS1 | hsa-miR-194-5p | LINC01833 |
| TRIM31-AS1 | hsa-miR-937-3p | CYP11B1 |
| LINC01208 | hsa-miR-372-3p | AF274573.1 |
| CASC16 | hsa-miR-4788 | FUT9 |
| UCA1 | hsa-miR-365a-5p | LINC00942 |
| LINC01354 | hsa-miR-205-5p | PASD1 |
| LINC00670 | hsa-miR-758-3p | AL513304.1 |
| LINC00160 | hsa-miR-3617-3p | ONECUT1 |
| LHFPL3-AS2 | hsa-miR-26a-1-3p | AL355075.4 |
| LINC01515 | hsa-miR-551b-3p | H2AC14 |
| LINC00866 | hsa-miR-675-3p | SP3P |
| PTGES2-AS1 | hsa-miR-767-3p | AC061975.6 |
| PACRG-AS1 | hsa-miR-493-3p | FTHL17 |
| LINC01559 | hsa-miR-1269b | SPANXB1 |
| LINC00884 | hsa-miR-323a-3p | TCN1 |
| PCAT7 | hsa-miR-4766-3p | DSCR8 |
| HOTAIR | hsa-miR-4664-3p | BARX1-DT |
| DUXAP8 | hsa-miR-6744-3p | FAM83A-AS1 |
| AP000577.2 | hsa-miR-1248 | MAGEB1 |
| CALML3-AS1 | hsa-miR-3194-5p | SSX1 |
| STEAP2-AS1 | hsa-miR-891a-5p | LIPK |
| AC002066.1 | hsa-miR-6815-3p | OLFM4 |
| TINCR | hsa-miR-653-5p | PAX7 |
| LINC00460 | hsa-miR-376b-3p | HOXA13 |
| CASC9 | hsa-miR-556-3p | IGF2BP1 |
| C20orf197 | hsa-miR-217-5p | CLDN10-AS1 |
| LUCAT1 | hsa-miR-381-5p | AL354685.1 |
| LINC01168 | hsa-miR-1293 | CST1 |
| UMODL1-AS1 | hsa-miR-545-5p | SCARNA10 |
| HOXC13-AS | hsa-miR-431-5p | EEF1A2 |
| AC112721.2 | hsa-miR-1251-5p | LINC01559 |
| STAU2-AS1 | hsa-miR-154-3p | AC114786.2 |
| VWA8-AS1 | hsa-miR-3664-5p | BCAR4 |
| LINC01166 | hsa-miR-6728-5p | H2BC10 |
| LINC01537 | hsa-miR-570-3p | HOXB13 |
| TRPM2-AS | hsa-miR-503-3p | SLC18A3 |
| SATB2-AS1 | hsa-miR-487a-3p | INHA |
| C11orf97 | hsa-miR-375-3p | PHOX2B |
| FAM83C-AS1 | hsa-miR-122-5p | MAGEA4-AS1 |
| LINC00858 | hsa-miR-371a-5p | PADI1 |
| AP001626.1 | hsa-miR-6510-3p | OTX2 |
| FIRRE | hsa-miR-5698 | H4C6 |
| LINC00501 | hsa-miR-345-3p | ASNSP1 |
| HELLPAR | hsa-miR-889-3p | MUC6 |
| CASC8 | hsa-miR-449b-5p | AFAP1-AS1 |
| SFTA1P | hsa-miR-5571-3p | IL37 |
| AC093390.1 | hsa-miR-556-5p | KCNU1 |
| LINC00707 | hsa-miR-3662 | AL139023.1 |
| LINC00973 | hsa-miR-548au-3p | ADGRG7 |
| MAFA-AS1 | hsa-miR-449c-5p | NR0B1 |
| MYO16-AS1 | hsa-miR-489-5p | DMBX1 |
| CECR7 | hsa-miR-489-3p | RN7SKP203 |
| AC123023.1 | hsa-miR-373-3p | MIR548XHG |
| ELFN1-AS1 | hsa-miR-1229-3p | TM4SF20 |
| DLX6-AS1 | hsa-miR-548y | LIN28A |
| ATP2A1-AS1 | hsa-miR-6860 | MYEOV |
| LINC01169 | hsa-miR-6850-5p | SLCO1B1 |
| SALRNA1 | hsa-miR-615-3p | FAR2P1 |
| FLJ12825 | hsa-miR-205-3p | KRT75 |
| NAALADL2-AS2 | hsa-miR-431-3p | ANKS4B |
| AC108676.1 | hsa-miR-216b-5p | H2BC14 |
| TMEM75 | hsa-miR-206 | DPPA2 |
| FAM222A-AS1 | hsa-miR-371a-3p | SLC2A2 |
| LINC00519 | hsa-miR-3127-3p | TRIM15 |
| PIK3CD-AS2 | hsa-miR-7112-3p | NEUROD4 |
| LINC00466 | hsa-miR-514b-5p | AC026336.3 |
| LINC01561 | hsa-miR-3161 | COL11A1 |
| AC073316.2 | hsa-miR-449a | IL36RN |
| TMEM92-AS1 | hsa-miR-6715a-3p | SOX14 |
| LINC00636 | hsa-miR-552-5p | SNORA54 |
| RMST | hsa-miR-376a-3p | H2BC3 |
| HOXC-AS3 | hsa-miR-376a-5p | POU6F2-AS2 |
| AC128709.2 | hsa-miR-662 | ETNPPL |
| HAGLROS | hsa-miR-4658 | PRSS2 |
| TTLL11-IT1 | hsa-miR-892a | MIR3609 |
| ERVH48-1 | hsa-miR-513c-5p | LINC01980 |
| EXTL3-AS1 | hsa-miR-449b-3p | RMRP |
| LINC01214 | hsa-miR-216a-3p | LINC01194 |
| LINC01529 | hsa-miR-548f-3p | MMP13 |
| LINC01436 | hsa-miR-146a-3p | KISS1R |
| PRC1-AS1 | hsa-miR-1911-5p | FEZF1-AS1 |
| LINC01484 | hsa-miR-519a-5p | GNGT1 |
| AC062028.1 | hsa-miR-512-3p | KLK12 |
| LINC01234 | hsa-miR-518f-5p | CRABP1 |
| HPN-AS1 | hsa-miR-541-3p | PAH |
| ITGB2-AS1 | hsa-miR-541-5p | IL1RAPL2 |
| EGOT | hsa-miR-873-5p | AC016044.1 |
| NALCN-AS1 | hsa-miR-552-3p | SPRR1B |
| MIR503HG | hsa-miR-483-5p | LINC00973 |
| LINC00941 | hsa-miR-520g-3p | BRDT |
| AC022182.1 | hsa-miR-124-3p | SLC6A4 |
| LINC00491 | hsa-miR-520h | PRSS1 |
| LINC01587 | hsa-miR-522-3p | CALML3 |
| AC012501.2 |  | AC106785.2 |
| GATA6-AS1 |  | GAGE1 |
| LINC01564 |  | UGT2B15 |
| LINC01393 |  | HOXC10 |
| LINC01305 |  | DUSP13 |
| LINC00626 |  | AP005233.2 |
| LINC01287 |  | AC079160.1 |
| AC112721.1 |  | HHIPL2 |
| MIR137HG |  | Z98257.1 |
| LINC01213 |  | PIWIL3 |
| HHIP-AS1 |  | RN7SKP9 |
| LINC00944 |  | AC098592.1 |
| AC144450.1 |  | MAGEB16 |
| HOXA11-AS |  | AC007128.2 |
| POU6F2-AS2 |  | EIF4E1B |
| FNDC1-IT1 |  | NPSR1 |
| SRGAP3-AS2 |  | GABRA3 |
| MUC2 |  | AF127577.3 |
| LINC00165 |  | PPP1R14D |
| LINC01518 |  | G6PC |
| LINC00582 |  | GPX2 |
| LINC01281 |  | H4C3 |
| LINC01535 |  | AL121949.2 |
| LEMD1-AS1 |  | PRB1 |
| AC026904.1 |  | LINC02616 |
| LINC00954 |  | LINC01667 |
| NFE4 |  | KCNC2 |
| LINC01136 |  | GFY |
| LINC00682 |  | MUC13 |
| LINC01513 |  | LINC02163 |
| C8orf31 |  | HOXC13-AS |
| AC092484.1 |  | VGF |
| LINC00342 |  | KRT81 |
| PGM5P3-AS1 |  | RN7SKP227 |
| HOXC-AS1 |  | MYBPC1 |
| HHATL-AS1 |  | LINC01518 |
| LINC00518 |  | HTR3A |
| LINC00676 |  | DKK4 |
| SCARNA10 |  | SP9 |
| SYNPR-AS1 |  | TBXT |
| AC124861.1 |  | RNVU1-2A |
| LL22NC03-63E9.3 |  | CHGB |
| LINC00567 |  | SNORA80A |
| DPP10-AS1 |  | AP003900.1 |
| AC093159.1 |  | H1-1 |
| PLAC4 |  | AKR1B15 |
| TTC3-AS1 |  | LINC01287 |
| SSTR5-AS1 |  | NPSR1-AS1 |
| LINC01511 |  | RNA5-8SP4 |
| FAM167A-AS1 |  | UGT3A1 |
| BCAR4 |  | FABP7 |
| LINC01267 |  | PNPLA5 |
| TM4SF1-AS1 |  | ONECUT3 |
| LNX1-AS2 |  | LINC02484 |
| GS1-594A7.3 |  | AL359955.1 |
| ZNF571-AS1 |  | KLF2P1 |
| LINC01385 |  | DSCR4 |
| MIR31HG |  | HOXC-AS3 |
| AC092535.3 |  | TM4SF4 |
| AC008268.1 |  | HMGA2 |
| AC078942.1 |  | LNCAROD |
| LINC00824 |  | H3C11 |
| AC079466.1 |  | PSG6 |
| LHFPL3-AS1 |  | AC020907.1 |
| POU6F2-AS1 |  | LINC01214 |
| FLJ16779 |  | MUC2 |
| MYCNOS |  | FETUB |
| LINC00251 |  | HOXD11 |
| LINC01269 |  | SALL1 |
| HOXA10-AS |  | LINC02588 |
| DSCAM-AS1 |  | B3GNT6 |
| MIAT |  | LINC01511 |
| LINC01194 |  | C6orf15 |
| AC010148.1 |  | AKR1B10P1 |
| LINC00536 |  | UNC93A |
| H19 |  | FGF5 |
| AC003092.1 |  | BAGE2 |
| AC058791.1 |  | VAX1 |
| HOXB-AS4 |  | VCX3A |
| LINC00898 |  | H2AC4 |
| LINC01224 |  | INS |
| LINC00659 |  | LINC02178 |
| SAMSN1-AS1 |  | H3C12 |
| EHMT2-AS1 |  | CLDN6 |
| SMCR2 |  | H1-4 |
| AC073316.1 |  | DPYSL5 |
| AC008278.2 |  | LY6D |
| LINC00589 |  | FAR2P4 |
| PACERR |  | CALB1 |
| LINC01344 |  | COL2A1 |
| FAM181A-AS1 |  | AL117329.1 |
| LINC00184 |  | SLC9A4 |
| TBC1D3P1-DHX40P1 |  | LINC02377 |
| CASC19 |  | PSG9 |
| LINC01583 |  | CTAG2 |
| DEPDC1-AS1 |  | PGLYRP3 |
| LINC01299 |  | ERVV-2 |
| AP001619.2 |  | TMEM132D-AS1 |
| SPATA3-AS1 |  | PRSS56 |
| ST7-OT4 |  | SPANXC |
| CHL1-AS1 |  | ZIC3 |
| LINC00524 |  | AL033397.1 |
| SOX21-AS1 |  | LINC02525 |
| KIF25-AS1 |  | FEZF1 |
| AC010729.2 |  | SCARNA21 |
| AP001429.1 |  | CA9 |
| HNF1A-AS1 |  | SNORA84 |
| LINC00958 |  | SNORD17 |
| LINC01356 |  | LINC00392 |
| ARNTL2-AS1 |  | PANCR |
| AC072062.1 |  | LINC02476 |
| LINC01206 |  | UCA1 |
| LINC01571 |  | CRABP2 |
| C5orf66-AS1 |  | EFNA2 |
| LINC01449 |  | AC093791.1 |
| LINC00943 |  | LINC02582 |
| LINC00592 |  | RN7SL3 |
| LINC00601 |  | LINC02404 |
| LINC01116 |  | AL133325.3 |
| MEG3 |  | LHX1-DT |
| LINC00648 |  | LINC01611 |
| TMLHE-AS1 |  | LINC00491 |
| P3H2-AS1 |  | RNVU1-2 |
| DPYD-AS1 |  | PSG8 |
| LINC01524 |  | PNMA5 |
| LINC01429 |  | AC120498.4 |
| AC092652.1 |  | ATOH1 |
| LINC00355 |  | AC010595.1 |
| ABCA9-AS1 |  | NGB |
| LINC01036 |  | SLCO1B7 |
| LINC01543 |  | AC055717.2 |
| LINC01485 |  | PLA2G2F |
| MIR181A1HG |  | RNU5E-1 |
| ZFHX4-AS1 |  | FAM133A |
| BFSP2-AS1 |  | RNVU1-31 |
| ZBTB20-AS1 |  | CYCSP6 |
| LINC00221 |  | BBOX1-AS1 |
| AGBL1-AS1 |  | SNORA74D |
| SNAP47-AS1 |  | KRT6A |
| LINC01419 |  | KIF1A |
| FER1L6-AS2 |  | AC120498.7 |
| CTD-3080P12.3 |  | CDX2 |
| LINC00668 |  | ATP10B |
| LINC01202 |  | LINC01036 |
| HAR1B |  | SALL3 |
| TBL1XR1-AS1 |  | AL133370.1 |
| C15orf54 |  | KRT83 |
| LINC00319 |  | MAGEA8 |
| CTD-2297D10.2 |  | MIR9-1HG |
| PRKCA-AS1 |  | CASC9 |
| AC099342.1 |  | DMRTC2 |
| AF230666.2 |  | CHAT |
| LINC00543 |  | SOHLH1 |
| TUSC8 |  | KCNMB2-AS1 |
| LINC00330 |  | NLRP5 |
| CPS1-IT1 |  | KCNJ18 |
| DSG1-AS1 |  | PPP4R3C |
| LINC01456 |  | SYT12 |
| SH3PXD2A-AS1 |  | AC122134.1 |
| LINC01257 |  | AC010789.1 |
| LINC00393 |  | H2AC12 |
| LINC01397 |  | ZIC5 |
| LINC00645 |  | NAA11 |
| LINC01446 |  | PRDM9 |
| AC092198.1 |  | AC007128.1 |
| LINC01098 |  | LINC01234 |
| FMR1-AS1 |  | LINC01299 |
| LINC01249 |  | BNIP5 |
| GRM5-AS1 |  | H4C2 |
| DENND5B-AS1 |  | SPANXD |
| SLC8A1-AS1 |  | H2AC16 |
| VCAN-AS1 |  | H4C4 |
| GDNF-AS1 |  | AC068228.1 |
| LINC00698 |  | LINC02267 |
| PROX1-AS1 |  | AC090809.1 |
| LINC00470 |  | S100A7 |
| FAM230C |  | ABCA12 |
| AC005307.1 |  | CSAG2 |
| LINC01185 |  | TERT |
| LINC00602 |  | PCDH8 |
| LINC01096 |  | SLCO1B3-SLCO1B7 |
| LINC00392 |  | AP000542.2 |
| SIRPG-AS1 |  | CEACAM7 |
| LINC01399 |  | PCP4 |
| AC009264.1 |  | AC025252.2 |
| LDLRAD4-AS1 |  | CCDC198 |
| AC002401.1 |  | LHX8 |
| AC104794.4 |  | AL365181.2 |
| RMRP |  | PPY2P |
| BRWD1-AS1 |  | AC106771.1 |
| LINC01556 |  | ZFPM2-AS1 |
| LSAMP-AS1 |  | ZYG11A |
| ATP13A4-AS1 |  | POU4F1 |
| FOXCUT |  | SCARNA6 |
| GACAT3 |  | BPIFA2 |
| AP004372.1 |  | KCNQ2 |
| LINC00871 |  | AC112495.1 |
| IFNG-AS1 |  | CLPSL2 |
| AL133243.4 |  | KHDC1L |
| ST8SIA6-AS1 |  | AC012213.1 |
| FLJ42969 |  | AC069061.2 |
| OVAAL |  | NAALADL2-AS2 |
| ARHGAP26-AS1 |  | GAL |
| CASC20 |  | LINC00501 |
| LINC01204 |  | ADAM6 |
| AC015923.1 |  | RN7SL151P |
| FGF12-AS2 |  | C3P1 |
| LINC00706 |  | CDH17 |
| LINC01079 |  | LINC02492 |
| LINC01210 |  | NKX2-4 |
| MIR663AHG |  | PRDM13 |
| LINC00308 |  | TLX1 |
| FOXP1-IT1 |  | NKAIN3 |
| HOTTIP |  | LINC01345 |
| AC106875.1 |  | H4C1 |
| LINC01411 |  | AC026462.3 |
| GS1-24F4.2 |  | H4C5 |
| GPR1-AS |  | LINC00682 |
| LINC00970 |  | AC120036.1 |
| CHODL-AS1 |  | FAM230C |
| LINC01346 |  | AC080037.2 |
| LINC01391 |  | MMP1 |
| NHS-AS1 |  | AL606490.8 |
| AC093911.1 |  | AL163953.1 |
| LCMT1-AS2 |  | MMP11 |
| LINC01502 |  | OTOS |
| SLC7A11-AS1 |  | AL138760.1 |
| TESC-AS1 |  | AC003092.1 |
| AC104088.1 |  | AL356274.2 |
| GS1-279B7.1 |  | LINC01385 |
| SCHLAP1 |  | TBX10 |
| LINC01312 |  | PTPRH |
| LINC01117 |  | CRYBA2 |
| AC078852.2 |  | EPYC |
| ASH1L-IT1 |  | APCS |
| SLCO4A1-AS1 |  | AC010329.2 |
| AC016710.1 |  | BPIFA1 |
| LINC01297 |  | ISX |
| LINC01221 |  | TAS2R2P |
| LINC00540 |  | ZIC2 |
| TRPC7-AS1 |  | AC116049.1 |
| LINC01342 |  | LINC01608 |
| GRM7-AS3 |  | KRT33A |
| IL20RB-AS1 |  | SNORA74B |
| SCARNA9 |  | GLB1L3 |
| RBAKDN |  | AL121949.3 |
| LINC01444 |  | SSTR5-AS1 |
| AC002511.2 |  | LINC02466 |
| FGF12-AS3 |  | ITPKA |
| BIRC6-AS2 |  | BX276092.7 |
| LINC00705 |  | PRSS3 |
| MACC1-AS1 |  | SNRPCP1 |
| OTX2-AS1 |  | GCNT3 |
| NAALADL2-AS3 |  | RNU1-88P |
| LINC01143 |  | AC122710.1 |
| LINC00922 |  | AC012501.2 |
| GPC5-AS1 |  | AL135929.2 |
| E2F3-IT1 |  | AC100826.1 |
| LINC00051 |  | G2E3-AS1 |
| AC092168.2 |  | AL157778.1 |
| PAQR9-AS1 |  | MNX1-AS1 |
| LINC01361 |  | RNF186 |
| XXYLT1-AS1 |  | LINC02335 |
| ZBTB46-AS1 |  | AC096656.1 |
| AC006262.4 |  | AC106799.3 |
| SPANXA2-OT1 |  | HAVCR1 |
| IGF2-AS |  | LINC01305 |
| SLC26A4-AS1 |  | PAGE1 |
| MRGPRG-AS1 |  | H2AC21 |
| GACAT2 |  | TUBAP13 |
| MYHAS |  | AC023824.5 |
| PLCB1-IT1 |  | AL513548.1 |
| RAPGEF4-AS1 |  | CHRNA9 |
| FLJ36000 |  | GJB6 |
| ATP6V1B1-AS1 |  | AL353693.1 |
| LINC01433 |  | FADS6 |
| HOXD-AS2 |  | NKX2-2 |
| AP000997.2 |  | PRAC2 |
| LINC01250 |  | DDX53 |
| C21orf91-OT1 |  | FOXG1 |
| PCAT18 |  | PIWIL1 |
| AC008592.4 |  | RN7SKP71 |
| LINC01322 |  | LHX5 |
| CHL1-AS2 |  | AL513123.1 |
| LINC00456 |  | ZNF280A |
| LINC01087 |  | GTF2IP6 |
| MIR205HG |  | ARL14 |
| AC093702.1 |  | LINC00221 |
| LINC00867 |  | AC116345.1 |
| AC008277.1 |  | CSAG3 |
| TDRG1 |  | PAGE5 |
| LINC00879 |  | AC008163.1 |
| LINC01133 |  | AL355596.1 |
| LINC01448 |  | LINC00858 |
| EPHA5-AS1 |  | EVX1 |
| MEG9 |  | HOXC-AS2 |
| ST7-AS2 |  | SLC30A10 |
| AC004009.1 |  | AC105290.1 |
| MIR7-3HG |  | AC106799.2 |
| TCEAL3-AS1 |  | AL445647.1 |
| AC106053.1 |  | NPW |
| AC096669.1 |  | H2BC7 |
| AC012485.2 |  | AL606469.1 |
| EDNRB-AS1 |  | LASTR |
| DPYD-AS2 |  | MROH3P |
| LINC01450 |  | PAGE2B |
| TCL6 |  | S100P |
| AC009303.2 |  | SNORA53 |
| LINC01163 |  | AC097512.1 |
| CLYBL-AS2 |  | NXPH1 |
| CACNA1C-IT3 |  | ASCL1 |
| UPK1A-AS1 |  | TRIM51BP |
| LINC00609 |  | RTBDN |
| FER1L6-AS1 |  | PSMD10P2 |
| LINC01060 |  | RPSAP52 |
| AC004053.1 |  | TAC3 |
| B3GALT5-AS1 |  | ANKRD34B |
| AC023481.1 |  | ARSH |
| AP000439.2 |  | NTS |
| PSORS1C3 |  | PRSS29P |
| LINC01551 |  | SLC6A10P |
| C10orf71-AS1 |  | PPP2R2C |
| LINC01362 |  | ARHGAP36 |
| AC003958.2 |  | ECEL1 |
| CYP4F26P |  | AKR1C4 |
| AP000997.3 |  | IGFL1P2 |
| TLR8-AS1 |  | AKR7A3 |
| TUNAR |  | GREM1 |
| ITGB5-AS1 |  | AC016687.3 |
| DIO2-AS1 |  | CSAG4 |
| OSBPL10-AS1 |  | LINC01807 |
| UCHL1-AS1 |  | DMRTA2 |
| AL589986.2 |  | LINC01896 |
| PART1 |  | AC025575.2 |
| ASTN2-AS1 |  | SERPINB4 |
| AC008937.2 |  | AC131211.1 |
| EGLN3-AS1 |  | LINC02253 |
| AC009313.1 |  | AC092484.1 |
| LINC01192 |  | LINC01633 |
| AC012506.3 |  | NPY |
| LINC00488 |  | SLC24A2 |
| MTOR-AS1 |  | AOC1 |
| KC6 |  | SNORA79B |
| DSCR10 |  | AC083809.1 |
| LINC00200 |  | LINC01395 |
| LINC01425 |  | AL691420.1 |
| C8orf17 |  | AL139327.1 |
| LINC01470 |  | LINC02783 |
| LINC00440 |  | MAGEB2 |
| AC067956.1 |  | LHFPL5 |
| ARHGEF3-AS1 |  | FOXR2 |
| LINC01443 |  | C1QL2 |
| LNX1-AS1 |  | OLIG3 |
| LINC00581 |  | ADAM2 |
| LINC01574 |  | ZIC4 |
| LINC01179 |  | AC091179.1 |
| DBET |  | ONECUT2 |
| LINC00939 |  | ZNF716 |
| LINC00485 |  | AGER |
| MIR124-2HG |  | UGT2A3 |
| C5orf17 |  | SIRLNT |
| LINC01580 |  | LRRC38 |
| NFIA-AS1 |  | UGT2B28 |
| C18orf15 |  | AL390318.1 |
| DLGAP1-AS5 |  | SYT14 |
| AC110769.3 |  | PHF2P2 |
| LPP-AS1 |  | MIR137HG |
| MIR3976HG |  | RN7SL434P |
| DISC1-IT1 |  | LBP |
| AP000462.2 |  | AL589182.1 |
| LINC01475 |  | LINC01831 |
| RASA2-IT1 |  | AL133284.1 |
| AC064874.1 |  | KLK8 |
| ARHGAP26-IT1 |  | TPTE |
| AC005863.1 |  | DEFA8P |
| LINC01040 |  | DEFA9P |
| AC016735.1 |  | CA10 |
| BIRC6-AS1 |  | AC005090.1 |
| DOCK4-AS1 |  | LINC01804 |
| ATG10-AS1 |  | H2AC13 |
| EMX2OS |  | SPRR2D |
| AP000462.1 |  | AC011632.1 |
| LINC01549 |  | AC010329.5 |
| SMAD1-AS2 |  | ABCC2 |
| ANKRD62P1-PARP4P3 |  | LINC02490 |
| ATP11A-AS1 |  | PDCL2 |
| MAGI1-AS1 |  | CD300LG |
| PHEX-AS1 |  | AC023421.1 |
| HECW1-IT1 |  | DLX6 |
| SLC9A9-AS1 |  | PLUT |
| LINC01067 |  | NDUFA5P6 |
| LINC01555 |  | CBY2 |
| PCA3 |  | LINC02830 |
| LINC01205 |  | LINC01977 |
| AC023347.1 |  | CDH18 |
| C8orf87 |  | LINC01748 |
| LINC00399 |  | RN7SL342P |
| RC3H1-IT1 |  | CLEC2A |
| CACNA1C-AS4 |  | ZIC1 |
| AC116609.1 |  | STRA8 |
| ZBTB20-AS3 |  | LINC02016 |
| STX18-IT1 |  | BNIP3P6 |
| AIRN |  | TAC1 |
| GPC6-AS1 |  | CLPSL1 |
| USP12-AS1 |  | TMPRSS4 |
| BACH1-IT3 |  | AC011294.1 |
| LINC01522 |  | AC012213.4 |
| FOXN3-AS2 |  | AC023824.6 |
| ZNF385D-AS1 |  | ALX3 |
| SMG6-IT1 |  | RNU1-11P |
| AC003092.2 |  | CNGA3 |
| MAST4-IT1 |  | SOX11 |
| DOCK9-AS1 |  | LINC01210 |
| LINC00454 |  | LINC00393 |
| LINC01532 |  | FABP4 |
| LINC01019 |  | RNA5-8SP6 |
| LINC01242 |  | SOHLH2 |
|  |  | ZMAT4 |
|  |  | AC073365.1 |
|  |  | AL589655.1 |
|  |  | KRT31 |
|  |  | HRG |
|  |  | GACAT3 |
|  |  | LINC01202 |
|  |  | H2BC17 |
|  |  | PLAC1 |
|  |  | DCAF8L2 |
|  |  | CEACAM5 |
|  |  | AC114786.1 |
|  |  | AF279873.3 |
|  |  | C12orf56 |
|  |  | KRT16 |
|  |  | H2AC17 |
|  |  | AL138789.1 |
|  |  | CNMD |
|  |  | SPP1 |
|  |  | AC125603.2 |
|  |  | LINC01249 |
|  |  | SNORA12 |
|  |  | SNORA63D |
|  |  | B4GALNT4 |
|  |  | OR56A3 |
|  |  | TMEM82 |
|  |  | BMP2KL |
|  |  | AC117386.2 |
|  |  | AC007159.1 |
|  |  | AL391863.2 |
|  |  | YBX2 |
|  |  | TUBB3 |
|  |  | SEPTIN14 |
|  |  | LIPF |
|  |  | SLC7A10 |
|  |  | NETO1 |
|  |  | TF |
|  |  | PDIA2 |
|  |  | CPS1-IT1 |
|  |  | RN7SL507P |
|  |  | BPIFB2 |
|  |  | SKOR2 |
|  |  | LINC02474 |
|  |  | RNA5-8SN1 |
|  |  | AC005993.1 |
|  |  | NKAIN1 |
|  |  | AC117386.1 |
|  |  | BNIP3P2 |
|  |  | PRKCA-AS1 |
|  |  | AC010275.1 |
|  |  | TBC1D3P1-DHX40P1 |
|  |  | AC006484.1 |
|  |  | SLITRK1 |
|  |  | AC084357.3 |
|  |  | LHX2 |
|  |  | BPIFA4P |
|  |  | AC011352.3 |
|  |  | SERTM1 |
|  |  | RNU6-140P |
|  |  | AC098826.2 |
|  |  | MUC3A |
|  |  | NSG2 |
|  |  | RPS16P9 |
|  |  | FOXB1 |
|  |  | CYP4F23P |
|  |  | ERVH48-1 |
|  |  | FP325330.3 |
|  |  | DSG3 |
|  |  | RN7SL769P |
|  |  | H2BC9 |
|  |  | LINC00645 |
|  |  | CABYR |
|  |  | ENTPD8 |
|  |  | SFTPC |
|  |  | A2ML1 |
|  |  | DSG4 |
|  |  | AL139274.1 |
|  |  | MRPL45P1 |
|  |  | NTSR1 |
|  |  | LINC01257 |
|  |  | SCARNA1 |
|  |  | DLX6-AS1 |
|  |  | LINC01924 |
|  |  | AL359095.1 |
|  |  | TEX19 |
|  |  | LINC02122 |
|  |  | AKR1C2 |
|  |  | TGIF2LX |
|  |  | AL355796.1 |
|  |  | ZPLD1 |
|  |  | PSLNR |
|  |  | METTL11B |
|  |  | RFX6 |
|  |  | CACNA2D1-AS1 |
|  |  | CSTL1 |
|  |  | AC026355.3 |
|  |  | RN7SL217P |
|  |  | RN7SL149P |
|  |  | RTL1 |
|  |  | THRA1/BTR |
|  |  | UGT1A6 |
|  |  | LCN15 |
|  |  | COL10A1 |
|  |  | RN7SKP189 |
|  |  | NMU |
|  |  | C8orf74 |
|  |  | SPRR3 |
|  |  | GJB2 |
|  |  | DSG1-AS1 |
|  |  | GH2 |
|  |  | AL589642.2 |
|  |  | AC104793.1 |
|  |  | RHBG |
|  |  | LGSN |
|  |  | LINC00898 |
|  |  | PITX1 |
|  |  | FMR1NB |
|  |  | CSF3 |
|  |  | XAGE1A |
|  |  | LINC01346 |
|  |  | GABRA5 |
|  |  | LINC01456 |
|  |  | AC098798.1 |
|  |  | UGT1A1 |
|  |  | LINC02866 |
|  |  | DUSP9 |
|  |  | ITLN2 |
|  |  | NLRP3P1 |
|  |  | GPR1-AS |
|  |  | AC106799.1 |
|  |  | AL611929.1 |
|  |  | FGF21 |
|  |  | AC022784.1 |
|  |  | ITPRID1 |
|  |  | APOA2 |
|  |  | ADAMTS20 |
|  |  | PLCB1-IT1 |
|  |  | H2BC1 |
|  |  | GLYATL2 |
|  |  | KNOP1P5 |
|  |  | PLAC4 |
|  |  | SPTSSB |
|  |  | ADGRF4 |
|  |  | FSTL5 |
|  |  | AC096759.2 |
|  |  | LINC02506 |
|  |  | LINC02109 |
|  |  | AC125603.1 |
|  |  | SCARNA7 |
|  |  | LINC02293 |
|  |  | HMX2 |
|  |  | TMEM229A |
|  |  | CELF3 |
|  |  | UGT1A3 |
|  |  | AC018541.1 |
|  |  | CNTNAP4 |
|  |  | H3Y1 |
|  |  | AL356133.1 |
|  |  | UGT1A7 |
|  |  | FGF3 |
|  |  | LINC01687 |
|  |  | FGF4 |
|  |  | TCAM1P |
|  |  | AL356057.1 |
|  |  | GLYATL3 |
|  |  | STRA6 |
|  |  | MAGEA9 |
|  |  | A1CF |
|  |  | HSD3B2 |
|  |  | LINC00707 |
|  |  | AC105118.1 |
|  |  | LINC02448 |
|  |  | CCDC185 |
|  |  | AC106794.1 |
|  |  | AL365181.3 |
|  |  | AC008496.2 |
|  |  | NR2E1 |
|  |  | AC243972.2 |
|  |  | LINC02152 |
|  |  | HTR3C |
|  |  | FAM107A |
|  |  | ERVV-1 |
|  |  | AC106900.1 |
|  |  | MYOC |
|  |  | C20orf141 |
|  |  | AC095050.1 |
|  |  | SYNPR |
|  |  | INSM1 |
|  |  | HORMAD1 |
|  |  | AC004870.2 |
|  |  | FCN3 |
|  |  | GNG4 |
|  |  | SERPINB5 |
|  |  | LINC02313 |
|  |  | PRAP1 |
|  |  | NPC1L1 |
|  |  | AL008638.2 |
|  |  | FER1L6-AS2 |
|  |  | XAGE5 |
|  |  | POU6F2-AS1 |
|  |  | AL159987.1 |
|  |  | RBM46 |
|  |  | COL25A1 |
|  |  | FOXE1 |
|  |  | PIGFP2 |
|  |  | SLC28A2 |
|  |  | LINC02411 |
|  |  | AC020891.2 |
|  |  | AF121898.1 |
|  |  | IGKV2-40 |
|  |  | SLCO6A1 |
|  |  | B3GNT3 |
|  |  | PRB3 |
|  |  | OFCC1 |
|  |  | SAGE1 |
|  |  | PTPRN |
|  |  | RN7SKP186 |
|  |  | AC111149.2 |
|  |  | LINC01446 |
|  |  | ZNF695 |
|  |  | AC040174.1 |
|  |  | LINC00355 |
|  |  | ZNF736P9Y |
|  |  | SCARNA13 |
|  |  | CDK5R2 |
|  |  | FOXL2 |
|  |  | RN7SL564P |
|  |  | SBSN |
|  |  | CLEC4M |
|  |  | AL606970.1 |
|  |  | AC026120.1 |
|  |  | AC103705.1 |
|  |  | AC090192.2 |
|  |  | FAM9A |
|  |  | RN7SKP79 |
|  |  | GPM6A |
|  |  | AP005262.1 |
|  |  | AC105460.1 |
|  |  | AC103702.2 |
|  |  | HOXA11-AS |
|  |  | IRX4 |
|  |  | LINC02535 |
|  |  | AC004704.1 |
|  |  | AC093515.1 |
|  |  | LINC01996 |
|  |  | CASC19 |
|  |  | GUCA1A |
|  |  | ALPI |
|  |  | CHRM1 |
|  |  | SCARNA3 |
|  |  | H2AC7 |
|  |  | LINC00518 |
|  |  | ANKRD1 |
|  |  | AC009262.1 |
|  |  | HTR3B |
|  |  | AL356274.1 |
|  |  | AC100800.1 |
|  |  | AC034223.2 |
|  |  | CXCL14 |
|  |  | 5_8S_rRNA |
|  |  | AC023824.1 |
|  |  | CCNP |
|  |  | AP000542.1 |
|  |  | AL391056.1 |
|  |  | HBA1 |
|  |  | AC007326.1 |
|  |  | LINC00824 |
|  |  | TRIM72 |
|  |  | IVL |
|  |  | AC084030.1 |
|  |  | FOXL2NB |
|  |  | TAF7L |
|  |  | IL36G |
|  |  | KC877982.1 |
|  |  | DMRT1 |
|  |  | PINCR |
|  |  | OBP2A |
|  |  | AC099520.1 |
|  |  | AC005972.1 |
|  |  | MARCHF11 |
|  |  | B3GALT1-AS1 |
|  |  | RNU6-438P |
|  |  | SNORD15B |
|  |  | TMEM75 |
|  |  | RNU5A-8P |
|  |  | MNX1 |
|  |  | LINC02428 |
|  |  | H2AC9P |
|  |  | OR13Z2P |
|  |  | AC073316.1 |
|  |  | GPR50 |
|  |  | NEIL3 |
|  |  | IGFBP1 |
|  |  | RNU2-23P |
|  |  | PES1P1 |
|  |  | AL390334.1 |
|  |  | SYNGR4 |
|  |  | AF178030.1 |
|  |  | TMEM100 |
|  |  | NFE4 |
|  |  | CA4 |
|  |  | LINC00466 |
|  |  | LINC00460 |
|  |  | LINC02133 |
|  |  | RETNLB |
|  |  | AC023301.1 |
|  |  | AC092916.1 |
|  |  | DNAJC12 |
|  |  | VCX |
|  |  | AL031767.1 |
|  |  | SULT1C3 |
|  |  | SLC38A8 |
|  |  | LGALS13 |
|  |  | AL109946.1 |
|  |  | AC016550.2 |
|  |  | NCAPD2P1 |
|  |  | PGLYRP4 |
|  |  | RAB3B |
|  |  | LINC02697 |
|  |  | AL136981.1 |
|  |  | AC024595.1 |
|  |  | SPDYC |
|  |  | AC019278.1 |
|  |  | CPLX2 |
|  |  | CASC16 |
|  |  | NKX2-5 |
|  |  | ANKRD40CL |
|  |  | OR4C6 |
|  |  | GIP |
|  |  | LINC01967 |
|  |  | NLRP11 |
|  |  | LINC00879 |
|  |  | SMIM24 |
|  |  | SIM1 |
|  |  | FREM3 |
|  |  | FER1L6-AS1 |
|  |  | CA6 |
|  |  | PPP1R1AP2 |
|  |  | AL139393.2 |
|  |  | H2BC6 |
|  |  | SNORA40B |
|  |  | ISL1 |
|  |  | UMODL1 |
|  |  | RNU6-681P |
|  |  | AC089983.1 |
|  |  | AL356234.2 |
|  |  | SNRPGP18 |
|  |  | AL606517.2 |
|  |  | MEX3A |
|  |  | LINP1 |
|  |  | UBE2C |
|  |  | TMPRSS11D |
|  |  | RN7SL296P |
|  |  | AL356364.1 |
|  |  | LINC02416 |
|  |  | AC106875.1 |
|  |  | AC023813.2 |
|  |  | HOXA10 |
|  |  | TAS2R38 |
|  |  | AC093281.2 |
|  |  | HOXA11 |
|  |  | LINC01992 |
|  |  | MAFA-AS1 |
|  |  | NXPH4 |
|  |  | PCMTD1P3 |
|  |  | BARHL2 |
|  |  | AP002957.1 |
|  |  | COL17A1 |
|  |  | CDK8P2 |
|  |  | SLC25A48 |
|  |  | TMEM59L |
|  |  | AL121949.1 |
|  |  | SLC14A2 |
|  |  | MTND2P2 |
|  |  | LGI3 |
|  |  | PSG7 |
|  |  | AC128709.3 |
|  |  | SYT13 |
|  |  | GABRG2 |
|  |  | CALCB |
|  |  | HNRNPA1P29 |
|  |  | LINC00251 |
|  |  | FER1L4 |
|  |  | AC113346.1 |
|  |  | LINC02159 |
|  |  | AC069277.1 |
|  |  | SNORA22C |
|  |  | HBB |
|  |  | PSG2 |
|  |  | HOXA10-AS |
|  |  | AC073578.1 |
|  |  | LYPD8 |
|  |  | AC022335.1 |
|  |  | RBFOX1 |
|  |  | SLC30A6P1 |
|  |  | PYDC1 |
|  |  | AC021534.1 |
|  |  | AZGP1P1 |
|  |  | LINC02473 |
|  |  | FOXH1 |
|  |  | IL17C |
|  |  | AL133467.4 |
|  |  | INA |
|  |  | LINC00461 |
|  |  | LINC02820 |
|  |  | COBLP1 |
|  |  | LINC02003 |
|  |  | AC007368.1 |
|  |  | RNF183 |
|  |  | AC090023.2 |
|  |  | LINC01597 |
|  |  | LINC01208 |
|  |  | ANGPT4 |
|  |  | AC090505.1 |
|  |  | CDHR2 |
|  |  | SH3GL3 |
|  |  | EN2 |
|  |  | HOXB-AS4 |
|  |  | AP005435.1 |
|  |  | CGREF1 |
|  |  | NAALADL2-AS3 |
|  |  | AC010729.2 |
|  |  | AP000859.1 |
|  |  | GPR87 |
|  |  | OR6K3 |
|  |  | MIR4713HG |
|  |  | CGB3 |
|  |  | IGFL2-AS1 |
|  |  | AL033397.2 |
|  |  | CPHL1P |
|  |  | APCDD1L |
|  |  | C2CD4A |
|  |  | AC113398.2 |
|  |  | AC015631.1 |
|  |  | IGKV1D-33 |
|  |  | AC140479.2 |
|  |  | ZPBP2 |
|  |  | WDR87 |
|  |  | AC069228.1 |
|  |  | SNORA38B |
|  |  | AC092139.1 |
|  |  | CLDN10 |
|  |  | SSX3 |
|  |  | LYPD1 |
|  |  | AC006270.2 |
|  |  | SNORA71A |
|  |  | PLPP4 |
|  |  | AC093001.1 |
|  |  | BCL2L10 |
|  |  | TRIM31 |
|  |  | TNFSF11 |
|  |  | AP002498.1 |
|  |  | AC025580.1 |
|  |  | RAD51AP1P1 |
|  |  | PRORY |
|  |  | IL31RA |
|  |  | LINC01082 |
|  |  | XDH |
|  |  | AL589740.1 |
|  |  | SERPINA7 |
|  |  | HBA2 |
|  |  | SEPTIN12 |
|  |  | AC024230.1 |
|  |  | AC010343.3 |
|  |  | AC009093.3 |
|  |  | GCNT1P4 |
|  |  | AC114803.1 |
|  |  | UPK3B |
|  |  | CEACAM18 |
|  |  | NLRP13 |
|  |  | REG3A |
|  |  | AP000696.1 |
|  |  | PURPL |
|  |  | AL133467.2 |
|  |  | CRLF1 |
|  |  | AC107308.1 |
|  |  | KRT6B |
|  |  | RPSAP71 |
|  |  | AC073347.1 |
|  |  | LINC02141 |
|  |  | H2BC8 |
|  |  | LINC01414 |
|  |  | SLC15A1 |
|  |  | RASAL1 |
|  |  | AL139420.2 |
|  |  | TFPI2-DT |
|  |  | AP000462.2 |
|  |  | AC018641.1 |
|  |  | IGKV1-33 |
|  |  | AC016710.1 |
|  |  | MYH6 |
|  |  | TMPRSS11A |
|  |  | RAET1L |
|  |  | AC112178.1 |
|  |  | KRT14 |
|  |  | WNT3A |
|  |  | MIOX |
|  |  | TMEM63C |
|  |  | AL158819.1 |
|  |  | AC254629.1 |
|  |  | AC104984.1 |
|  |  | RANBP20P |
|  |  | IGFL3 |
|  |  | AL139120.1 |
|  |  | RN7SL464P |
|  |  | PROC |
|  |  | LINC01886 |
|  |  | KLF2P4 |
|  |  | ALAS2 |
|  |  | CACNA1C-IT3 |
|  |  | SUN3 |
|  |  | RNU2-61P |
|  |  | MYCN |
|  |  | HOXD12 |
|  |  | AC114489.1 |
|  |  | LINC02263 |
|  |  | LINC01529 |
|  |  | IGF2BP3 |
|  |  | SLC6A3 |
|  |  | AC244107.1 |
|  |  | AC106744.1 |
|  |  | MLXP1 |
|  |  | AP003500.1 |
|  |  | FAM9C |
|  |  | LINC02672 |
|  |  | RTKN2 |
|  |  | NCAPGP2 |
|  |  | RN7SL314P |
|  |  | SCAT8 |
|  |  | UCN2 |
|  |  | SLC17A4 |
|  |  | LINC02029 |
|  |  | LINC00648 |
|  |  | AP002856.2 |
|  |  | POU6F2 |
|  |  | AC078923.1 |
|  |  | CD5L |
|  |  | AC087857.1 |
|  |  | AC108451.2 |
|  |  | SCARNA12 |
|  |  | AC025244.1 |
|  |  | PRLHR |
|  |  | CALY |
|  |  | RN7SKP48 |
|  |  | AL139420.1 |
|  |  | LINC02327 |
|  |  | MUC5B |
|  |  | AL161658.1 |
|  |  | AL139095.3 |
|  |  | LINC02048 |
|  |  | RN7SL786P |
|  |  | RPL32P33 |
|  |  | MMP12 |
|  |  | LCT |
|  |  | AC135012.3 |
|  |  | SLC39A5 |
|  |  | FOXD3 |
|  |  | USH1C |
|  |  | SLC6A15 |
|  |  | AL157402.2 |
|  |  | OR10H1 |
|  |  | APOC3 |
|  |  | CTCFL |
|  |  | RN7SL394P |
|  |  | TMEM179 |
|  |  | TNS4 |
|  |  | AC002378.1 |
|  |  | LINC02475 |
|  |  | RNVU1-28 |
|  |  | NF1P8 |
|  |  | AC005307.1 |
|  |  | MCEMP1 |
|  |  | MIA |
|  |  | AC064872.1 |
|  |  | MTCYBP32 |
|  |  | IGKV2D-28 |
|  |  | UGT2B10 |
|  |  | GSG1L2 |
|  |  | BECN2 |
|  |  | POU3F3 |
|  |  | AL645608.8 |
|  |  | TUBB2B |
|  |  | WNT16 |
|  |  | MTND5P26 |
|  |  | LINC00668 |
|  |  | CYP2B6 |
|  |  | AHSG |
|  |  | AC104984.4 |
|  |  | ZNF75BP |
|  |  | AL731684.1 |
|  |  | AL391427.1 |
|  |  | DCAF8L1 |
|  |  | AC092868.1 |
|  |  | AL391097.1 |
|  |  | LINC02086 |
|  |  | WFDC3 |
|  |  | FLJ36000 |
|  |  | MMP10 |
|  |  | LINC02014 |
|  |  | LGALS4 |
|  |  | HRH3 |
|  |  | SCHLAP1 |
|  |  | RN7SL480P |
|  |  | AC009135.1 |
|  |  | AC124290.2 |
|  |  | AC093787.1 |
|  |  | AC008763.3 |
|  |  | FGA |
|  |  | LINC01460 |
|  |  | FUT6 |
|  |  | BARX2 |
|  |  | ANGPTL3 |
|  |  | ZFHX4-AS1 |
|  |  | AC009065.5 |
|  |  | BPIFB6 |
|  |  | CARD18 |
|  |  | FRG2 |
|  |  | AL354714.1 |
|  |  | SEPTIN14P12 |
|  |  | TSPY2 |
|  |  | KRTAP2-3 |
|  |  | APOA4 |
|  |  | FP236383.5 |
|  |  | ATP4A |
|  |  | RN7SL791P |
|  |  | AC021876.1 |
|  |  | ALG1L3P |
|  |  | C5orf66-AS1 |
|  |  | HOMER2P1 |
|  |  | EPHA8 |
|  |  | MMP3 |
|  |  | LINC00887 |
|  |  | TOP2A |
|  |  | C10orf67 |
|  |  | EIF1AXP2 |
|  |  | LINC02332 |
|  |  | AC018692.2 |
|  |  | AC110813.1 |
|  |  | RNU6-77P |
|  |  | LINC01213 |
|  |  | AC135068.5 |
|  |  | HJURP |
|  |  | IL20RB |
|  |  | AC104794.4 |
|  |  | AC090125.1 |
|  |  | AC092969.1 |
|  |  | SCARNA22 |
|  |  | GLRA3 |
|  |  | SLC25A5P5 |
|  |  | H2AC8 |
|  |  | AC025881.1 |
|  |  | RPL13AP17 |
|  |  | AC110015.1 |
|  |  | OVCH1 |
|  |  | MAGEC3 |
|  |  | RNY4 |
|  |  | AC009884.1 |
|  |  | TFPI2 |
|  |  | AC022022.2 |
|  |  | GPD1 |
|  |  | LINC02037 |
|  |  | KIF4A |
|  |  | AC007431.2 |
|  |  | TEX13B |
|  |  | RN7SL116P |
|  |  | SCGB2A2 |
|  |  | AL136114.1 |
|  |  | ETV4 |
|  |  | AC087273.1 |
|  |  | RN7SL398P |
|  |  | AP000424.1 |
|  |  | TUNAR |
|  |  | AC027228.2 |
|  |  | FAM172BP |
|  |  | MYBL2 |
|  |  | TROAP |
|  |  | RN7SL7P |
|  |  | SMOC1 |
|  |  | RNU1-89P |
|  |  | AL445687.2 |
|  |  | DEPDC1 |
|  |  | RNVU1-7 |
|  |  | SULT4A1 |
|  |  | AC239584.1 |
|  |  | MCCD1 |
|  |  | AC018470.1 |
|  |  | AP003108.5 |
|  |  | AC010486.3 |
|  |  | CFHR4 |
|  |  | AC073316.2 |
|  |  | AC006357.1 |
|  |  | SSTR5 |
|  |  | AC087379.1 |
|  |  | IGHG4 |
|  |  | ATP11AUN |
|  |  | H2BC13 |
|  |  | C10orf90 |
|  |  | DEFB126 |
|  |  | PPP1R2P10 |
|  |  | ZNF560 |
|  |  | ELFN1-AS1 |
|  |  | DIMT1P1 |
|  |  | LINC00567 |
|  |  | SNORA20 |
|  |  | RIMS2 |
|  |  | AP005230.1 |
|  |  | HGD |
|  |  | LINC01221 |
|  |  | FENDRR |
|  |  | TEX37 |
|  |  | AC087269.1 |
|  |  | FBXO36P1 |
|  |  | HNF1A |
|  |  | ANKRD20A19P |
|  |  | AC012354.7 |
|  |  | ANLN |
|  |  | CASP14 |
|  |  | MYPN |
|  |  | DSPP |
|  |  | AC009065.2 |
|  |  | AL135929.1 |
|  |  | RN7SL191P |
|  |  | AC007639.1 |
|  |  | MIR924HG |
|  |  | KCNA4 |
|  |  | LINC01956 |
|  |  | AC091151.2 |
|  |  | LINC01711 |
|  |  | MUC5AC |
|  |  | AC091133.5 |
|  |  | TUBA3E |
|  |  | FER1L6 |
|  |  | CU104787.1 |
|  |  | HDGFL1 |
|  |  | TUSC8 |
|  |  | LINC01705 |
|  |  | CDC20 |
|  |  | LINC00862 |
|  |  | YWHAEP7 |
|  |  | SNORA38 |
|  |  | KIF18B |
|  |  | SAPCD2 |
|  |  | RNA5-8SP5 |
|  |  | RN7SL300P |
|  |  | RN7SL466P |
|  |  | FAM83C |
|  |  | KCNK9 |
|  |  | RPL23AP77 |
|  |  | BNIP3P7 |
|  |  | GAD1 |
|  |  | AC073578.2 |
|  |  | AC104332.1 |
|  |  | AC104232.2 |
|  |  | IGFL2 |
|  |  | CDC37P2 |
|  |  | ESX1 |
|  |  | CTHRC1 |
|  |  | DRAIC |
|  |  | C4orf54 |
|  |  | TNR |
|  |  | PHOX2A |
|  |  | H3C1 |
|  |  | ITGB1-DT |
|  |  | KIF14 |
|  |  | NCCRP1 |
|  |  | AF127577.6 |
|  |  | LINC01776 |
|  |  | RN7SL4P |
|  |  | AC084262.1 |
|  |  | VSX1 |
|  |  | SPRR1A |
|  |  | RN7SL643P |
|  |  | TFAP2A |
|  |  | RN7SKP90 |
|  |  | NEK2 |
|  |  | AC084026.3 |
|  |  | MUC21 |
|  |  | EDDM3CP |
|  |  | AL590666.2 |
|  |  | NRK |
|  |  | SLAMF6P1 |
|  |  | AC093904.4 |
|  |  | CNTNAP5 |
|  |  | PHKBP2 |
|  |  | LINC02789 |
|  |  | RASGEF1C |
|  |  | CR392039.3 |
|  |  | AP003467.2 |
|  |  | TBX15 |
|  |  | AC142384.1 |
|  |  | AF254983.1 |
|  |  | SGCG |
|  |  | E2F3-IT1 |
|  |  | RNU6-522P |
|  |  | AC090502.2 |
|  |  | ASPM |
|  |  | AC027627.1 |
|  |  | YBX1P7 |
|  |  | LINC00634 |
|  |  | BX842568.1 |
|  |  | PRODH2 |
|  |  | AC103740.2 |
|  |  | LINC01983 |
|  |  | AL162582.1 |
|  |  | CYP1A2 |
|  |  | LINC02303 |
|  |  | NPTX1 |
|  |  | AC090164.3 |
|  |  | RUNDC3A-AS1 |
|  |  | LILRP1 |
|  |  | LRRC37A9P |
|  |  | MSTN |
|  |  | IGLVI-70 |
|  |  | COCH |
|  |  | AC099509.1 |
|  |  | FRGCA |
|  |  | EMX2 |
|  |  | SNRPEP10 |
|  |  | PYCR1 |
|  |  | SLC6A11 |
|  |  | NUF2 |
|  |  | H1-12P |
|  |  | NTSR2 |
|  |  | SPINK13 |
|  |  | AC131009.2 |
|  |  | AC099487.1 |
|  |  | OR1F1 |
|  |  | SOX1 |
|  |  | H3C8 |
|  |  | MYBPH |
|  |  | RGS17 |
|  |  | DPYD-AS2 |
|  |  | C10orf88B |
|  |  | HMGB3P18 |
|  |  | IL22RA2 |
|  |  | LINC00871 |
|  |  | AC068535.1 |
|  |  | ABCA4 |
|  |  | SSTR4 |
|  |  | AC010776.3 |
|  |  | AC109454.3 |
|  |  | IGBP1P4 |
|  |  | HS6ST2 |
|  |  | BX005019.1 |
|  |  | MED15P9 |
|  |  | LINC00602 |
|  |  | AC079384.1 |
|  |  | TIMM9P1 |
|  |  | DLGAP5 |
|  |  | AC024405.2 |
|  |  | AC114878.1 |
|  |  | POU5F2 |
|  |  | BAIAP2L2 |
|  |  | RN7SL197P |
|  |  | LINC00659 |
|  |  | IGKV3OR2-5 |
|  |  | BIRC5 |
|  |  | AC090502.3 |
|  |  | TRIM54 |
|  |  | RN7SKP8 |
|  |  | GALNT14 |
|  |  | AC026117.1 |
|  |  | HEPACAM2 |
|  |  | AL160408.4 |
|  |  | AC021134.1 |
|  |  | ARHGAP40 |
|  |  | MAEL |
|  |  | LINC02438 |
|  |  | LINC01297 |
|  |  | STXBP5L |
|  |  | FP671120.3 |
|  |  | CDC25C |
|  |  | AL034349.1 |
|  |  | SOX3 |
|  |  | MYCNOS |
|  |  | AC133106.1 |
|  |  | CRHR1 |
|  |  | AC022973.1 |
|  |  | CASKIN1 |
|  |  | OTOG |
|  |  | MIR2052HG |
|  |  | AC016831.4 |
|  |  | TDRD5 |
|  |  | LY6K |
|  |  | TPX2 |
|  |  | RN7SL664P |
|  |  | B4GALNT2 |
|  |  | AC006065.4 |
|  |  | ZNF722P |
|  |  | AC034206.1 |
|  |  | TEX55 |
|  |  | RHOV |
|  |  | MTNR1A |
|  |  | KCNV1 |
|  |  | DDX43P3 |
|  |  | AC139792.3 |
|  |  | CYP4F8 |
|  |  | CRP |
|  |  | GKN1 |
|  |  | RN7SL14P |
|  |  | CRCT1 |
|  |  | AL445072.1 |
|  |  | LPP-AS1 |
|  |  | CHODL-AS1 |
|  |  | AC013268.3 |
|  |  | TSPY1 |
|  |  | GRIA1 |
|  |  | RN7SL508P |
|  |  | RN7SL359P |
|  |  | CENPA |
|  |  | AL079305.1 |
|  |  | EPHA10 |
|  |  | MELK |
|  |  | RHCG |
|  |  | RAB26 |
|  |  | RS1 |
|  |  | IQGAP3 |
|  |  | AC015849.5 |
|  |  | LINC01614 |
|  |  | AIG1P1 |
|  |  | AC073530.1 |
|  |  | LINC02196 |
|  |  | KIF18BP1 |
|  |  | AL121904.1 |
|  |  | AC069120.1 |
|  |  | F13B |
|  |  | HSPA8P13 |
|  |  | KIRREL2 |
|  |  | AC010997.6 |
|  |  | BNIP3P42 |
|  |  | AC092427.1 |
|  |  | GPIHBP1 |
|  |  | TMEM238L |
|  |  | AC120349.2 |
|  |  | SOSTDC1 |
|  |  | TEX101 |
|  |  | IAPP |
|  |  | EXO1 |
|  |  | CHRNA5 |
|  |  | DEPDC1B |
|  |  | SPATA16 |
|  |  | CHIAP2 |
|  |  | AC116666.1 |
|  |  | AF130417.1 |
|  |  | SLC25A48-AS1 |
|  |  | LINC00160 |
|  |  | ZNF679 |
|  |  | AC007848.1 |
|  |  | CDHR5 |
|  |  | CLEC3B |
|  |  | TEX48 |
|  |  | NUS1P2 |
|  |  | LINC00628 |
|  |  | BHMT |
|  |  | AC007494.3 |
|  |  | TESC |
|  |  | AL590226.1 |
|  |  | LINC01681 |
|  |  | RN7SL732P |
|  |  | KIF2C |
|  |  | AC106794.2 |
|  |  | TMEM14EP |
|  |  | AC096741.1 |
|  |  | EIF4E2P1 |
|  |  | AC090954.1 |
|  |  | AC022031.2 |
|  |  | ANGPTL7 |
|  |  | COL22A1 |
|  |  | RNU2-59P |
|  |  | AL365258.1 |
|  |  | AL118556.2 |
|  |  | PAQR9-AS1 |
|  |  | NDUFA8P1 |
|  |  | CRISP1 |
|  |  | AC012568.1 |
|  |  | GAST |
|  |  | AC012354.2 |
|  |  | RN7SL735P |
|  |  | AC130895.1 |
|  |  | FRMD5 |
|  |  | LINC02223 |
|  |  | AC012531.1 |
|  |  | ZBTB20-AS5 |
|  |  | AC083801.2 |
|  |  | SNORA37 |
|  |  | AC096721.1 |
|  |  | AC093895.1 |
|  |  | AC129507.4 |
|  |  | GABRG3 |
|  |  | LINC01444 |
|  |  | SNORA14A |
|  |  | SNORA14B |
|  |  | RDM1 |
|  |  | AL023755.1 |
|  |  | UHRF2P1 |
|  |  | AC010359.1 |
|  |  | LINC00601 |
|  |  | RAD17P2 |
|  |  | LARP1BP1 |
|  |  | AC091987.1 |
|  |  | MCM10 |
|  |  | AL022324.3 |
|  |  | CGB8 |
|  |  | AC006206.1 |
|  |  | CHCHD4P5 |
|  |  | SPINT4 |
|  |  | LINC01206 |
|  |  | VN1R54P |
|  |  | IGFL1 |
|  |  | RN7SL630P |
|  |  | AC005537.1 |
|  |  | AL049775.2 |
|  |  | COMETT |
|  |  | AC091133.3 |
|  |  | AL590632.1 |
|  |  | AC087855.2 |
|  |  | AC106744.2 |
|  |  | RPL35AP2 |
|  |  | ANKRD30BP2 |
|  |  | REG1B |
|  |  | AC141557.2 |
|  |  | RN7SL801P |
|  |  | SNORA35B |
|  |  | OTC |
|  |  | MIR3140 |
|  |  | CLIC5 |
|  |  | CDKN2A |
|  |  | SLC2A1 |
|  |  | AC005336.1 |
|  |  | GRHL3 |
|  |  | AC037487.2 |
|  |  | AP003559.1 |
|  |  | OR7A19P |
|  |  | KRT3 |
|  |  | AMBP |
|  |  | MIR3976HG |
|  |  | TEDC2 |
|  |  | RAX |
|  |  | LANCL1-AS1 |
|  |  | SNORA7B |
|  |  | TBL1XR1-AS1 |
|  |  | PLK5 |
|  |  | OR4A16 |
|  |  | BCL2L15 |
|  |  | LINC02826 |
|  |  | SLC5A5 |
|  |  | AC011270.2 |
|  |  | ZNF729 |
|  |  | GPR26 |
|  |  | DGCR5 |
|  |  | SMC1B |
|  |  | AF015262.1 |
|  |  | AC025419.1 |
|  |  | AC136759.1 |
|  |  | AC068985.1 |
|  |  | KIF3AP1 |
|  |  | MGAT4EP |
|  |  | LINC02544 |
|  |  | CENPF |
|  |  | DRAXINP1 |
|  |  | RPS17P11 |
|  |  | AC011352.1 |
|  |  | NKX3-2 |
|  |  | AC010328.1 |
|  |  | AP000829.1 |
|  |  | HMGB3 |
|  |  | GCGR |
|  |  | HNF4G |
|  |  | AL442224.1 |
|  |  | ACTL6B |
|  |  | CHRNA2 |
|  |  | VSIG1 |
|  |  | CP |
|  |  | PSAT1 |
|  |  | VEGFD |
|  |  | AC122694.1 |
|  |  | NFYAP1 |
|  |  | PTPRQ |
|  |  | NMRAL2P |
|  |  | RNU6-82P |
|  |  | AL512326.3 |
|  |  | GDA |
|  |  | CILP2 |
|  |  | PI16 |
|  |  | AC073651.1 |
|  |  | AC123905.1 |
|  |  | AC015574.1 |
|  |  | HPDL |
|  |  | LHFPL4 |
|  |  | IGKV1-13 |
|  |  | TMED11P |
|  |  | PNMA6F |
|  |  | AL135878.1 |
|  |  | AL645608.2 |
|  |  | DPPA5 |
|  |  | H3C4 |
|  |  | WFDC5 |
|  |  | MYO3B |
|  |  | AC093730.1 |
|  |  | RN7SL18P |
|  |  | ADM2 |
|  |  | PPP1R14B-AS1 |
|  |  | HOXB8 |
|  |  | LINC02385 |
|  |  | TRPM2-AS |
|  |  | AC114316.1 |
|  |  | LYZL2 |
|  |  | LINC01926 |
|  |  | RN7SL646P |
|  |  | AC093833.1 |
|  |  | TNNC1 |
|  |  | SCUBE1 |
|  |  | CARD14 |
|  |  | GABRQ |
|  |  | MIR3945HG |
|  |  | SYT7 |
|  |  | RN7SL767P |
|  |  | RN7SL273P |
|  |  | DNAJC22 |
|  |  | OR13K1P |
|  |  | OR2B6 |
|  |  | LINC01854 |
|  |  | THEG |
|  |  | AC009264.1 |
|  |  | LYPD3 |
|  |  | AL352984.1 |
|  |  | AURKB |
|  |  | GABRR1 |
|  |  | AL451137.2 |
|  |  | GUCY2EP |
|  |  | ADAMTS7P3 |
|  |  | SHOX2 |
|  |  | PHGR1 |
|  |  | AC144521.1 |
|  |  | AC090220.1 |
|  |  | SNORA46 |
|  |  | IQCM |
|  |  | ELAVL2 |
|  |  | LINC01842 |
|  |  | RN7SL546P |
|  |  | PACRG-AS3 |
|  |  | AC138305.1 |
|  |  | OGDHL |
|  |  | UCHL1 |
|  |  | AC027279.4 |
|  |  | AC092325.1 |
|  |  | TRIM49 |
|  |  | AC017099.1 |
|  |  | HHLA1 |
|  |  | OR7E62P |
|  |  | MOGAT3 |
|  |  | NANOGP1 |
|  |  | AL138713.1 |
|  |  | AC016769.3 |
|  |  | LINC00968 |
|  |  | CLDN18 |
|  |  | HOTTIP |
|  |  | LINC00629 |
|  |  | ADAMTS8 |
|  |  | CDC6 |
|  |  | LINC02398 |
|  |  | AC022537.1 |
|  |  | AC007599.1 |
|  |  | FBXL21P |
|  |  | RN7SL38P |
|  |  | TEX11 |
|  |  | MTND4P32 |
|  |  | AC008870.5 |
|  |  | IFNWP19 |
|  |  | AL136313.1 |
|  |  | GPT2 |
|  |  | RN7SL408P |
|  |  | AC074389.2 |
|  |  | IGLV3-29 |
|  |  | AC020551.1 |
|  |  | FMO9P |
|  |  | NCKAP5 |
|  |  | AC012065.1 |
|  |  | SSX5 |
|  |  | AC016716.1 |
|  |  | CASC20 |
|  |  | MAGEB6 |
|  |  | AC104574.2 |
|  |  | AL445183.2 |
|  |  | LINC01792 |
|  |  | INMT |
|  |  | RN7SL126P |
|  |  | GNAT3 |
|  |  | AP004607.7 |
|  |  | AL023754.1 |
|  |  | PCK1 |
|  |  | AC093904.2 |
|  |  | BUB1B |
|  |  | AL445523.1 |
|  |  | AC135626.1 |
|  |  | PSCA |
|  |  | CLCA3P |
|  |  | KLK14 |
|  |  | MTCO1P19 |
|  |  | LINC02192 |
|  |  | ATG10-AS1 |
|  |  | S100A7A |
|  |  | AC006372.1 |
|  |  | LINC02505 |
|  |  | AP003119.1 |
|  |  | AC007012.2 |
|  |  | RN7SKP133 |
|  |  | GJA10 |
|  |  | AP004289.1 |
|  |  | GPC2 |
|  |  | S100A2 |
|  |  | RN7SL659P |
|  |  | SLC13A5 |
|  |  | AC004865.1 |
|  |  | PLXNB3 |
|  |  | PBK |
|  |  | WASIR2 |
|  |  | LINC00626 |
|  |  | FOXM1 |
|  |  | COL6A4P1 |
|  |  | TAS2R30 |
|  |  | EDNRB |
|  |  | SALL4 |
|  |  | AL133370.2 |
|  |  | LINC00656 |
|  |  | AC009743.1 |
|  |  | AL627308.2 |
|  |  | CELSR3 |
|  |  | RNU6ATAC |
|  |  | RN7SL302P |
|  |  | FAM131C |
|  |  | DNASE1L3 |
|  |  | AL049539.1 |
|  |  | LRRC14B |
|  |  | IGHV1-24 |
|  |  | USH1G |
|  |  | AP000251.1 |
|  |  | GRM7-AS3 |
|  |  | LINC01543 |
|  |  | RN7SL308P |
|  |  | AC104164.2 |
|  |  | AC080129.1 |
|  |  | AC074237.1 |
|  |  | ZNF72P |
|  |  | AC100763.1 |
|  |  | KRT16P6 |
|  |  | DEFA3 |
|  |  | AP004289.2 |
|  |  | LINC00524 |
|  |  | CHRNB4 |
|  |  | AC003989.2 |
|  |  | AC105917.1 |
|  |  | NAA20P1 |
|  |  | AL450345.1 |
|  |  | H19 |
|  |  | CCKAR |
|  |  | AC090673.1 |
|  |  | AC097716.1 |
|  |  | ARMH2 |
|  |  | AC133561.1 |
|  |  | HMGA2-AS1 |
|  |  | UHRF1 |
|  |  | LINC02046 |
|  |  | MTND1P32 |
|  |  | CNTN6 |
|  |  | CDY4P |
|  |  | LINC01079 |
|  |  | AL451047.1 |
|  |  | C10orf71 |
|  |  | AC100791.2 |
|  |  | AC104237.3 |
|  |  | TEX15 |
|  |  | MAP7D2 |
|  |  | LINC02154 |
|  |  | ADRA1A |
|  |  | MKRN9P |
|  |  | SPRR2A |
|  |  | AL132709.7 |
|  |  | CU639417.5 |
|  |  | NAT8L |
|  |  | HMGB3P6 |
|  |  | AC003989.1 |
|  |  | IGLVIVOR22-1 |
|  |  | AC245128.1 |
|  |  | LINC00536 |
|  |  | OTX1 |
|  |  | KNG1 |
|  |  | AC092910.2 |
|  |  | AC023796.2 |
|  |  | AC004009.1 |
|  |  | LINC02387 |
|  |  | AC108136.1 |
|  |  | AC245100.3 |
|  |  | PABPC1L2B |
|  |  | AC084783.1 |
|  |  | FSHB |
|  |  | ARC |
|  |  | LINC00525 |
|  |  | MYOSLID |
|  |  | IGKV3D-7 |
|  |  | AL137803.1 |
|  |  | AL596223.1 |
|  |  | AC022148.1 |
|  |  | HMGB3P13 |
|  |  | HSPD1P13 |
|  |  | AC114402.1 |
|  |  | AC010947.1 |
|  |  | MIR31HG |
|  |  | AL035252.2 |
|  |  | AC010145.1 |
|  |  | CELF5 |
|  |  | CPP |
|  |  | AC104237.2 |
|  |  | AL079304.1 |
|  |  | USP24P1 |
|  |  | AC040174.2 |
|  |  | TTR |
|  |  | LINC01740 |
|  |  | BTNL9 |
|  |  | RN7SL2 |
|  |  | AL391863.1 |
|  |  | AC008060.1 |
|  |  | KCNK12 |
|  |  | HSD3B1 |
|  |  | DLX1 |
|  |  | FHL1 |
|  |  | AP000756.1 |
|  |  | MIR4527HG |
|  |  | AL021920.2 |
|  |  | SNX18P2 |
|  |  | AL445307.1 |
|  |  | LINC00896 |
|  |  | NR5A1 |
|  |  | SHISAL2B |
|  |  | SLC4A1 |
|  |  | CCDC92B |
|  |  | CFAP20DC-AS1 |
|  |  | AC004158.1 |
|  |  | RFPL4B |
|  |  | CREB3L2-AS1 |
|  |  | RPL37P3 |
|  |  | GRM4 |
|  |  | LINC02522 |
|  |  | RLIMP1 |
|  |  | AC016769.1 |
|  |  | CAV1 |
|  |  | SIX2 |
|  |  | AL359313.1 |
|  |  | UBE2T |
|  |  | AC090503.2 |
|  |  | RN7SL665P |
|  |  | CHRM2 |
|  |  | SCN4A |
|  |  | LINC02570 |
|  |  | AC119424.1 |
|  |  | AP003174.1 |
|  |  | AC058791.1 |
|  |  | AC009313.1 |
|  |  | PTPN5 |
|  |  | DMRTB1 |
|  |  | LINC01070 |
|  |  | RPS16P8 |
|  |  | GREB1L |
|  |  | EVX2 |
|  |  | TTK |
|  |  | LINC02365 |
|  |  | ERCC6L |
|  |  | IGHV3OR16-11 |
|  |  | AC020891.3 |
|  |  | LINC00880 |
|  |  | LL22NC03-63E9.3 |
|  |  | GAPDHP55 |
|  |  | KIF25-AS1 |
|  |  | SPTB |
|  |  | CCNE1 |
|  |  | POLQ |
|  |  | RHOXF2B |
|  |  | AL122126.1 |
|  |  | SLC1A6 |
|  |  | GJB3 |
|  |  | LINC00163 |
|  |  | CT47B1 |
|  |  | MAGEA10-MAGEA5 |
|  |  | SNHG27 |
|  |  | FAM177B |
|  |  | ERFE |
|  |  | RAD54L |
|  |  | AL157931.1 |
|  |  | GAGE12J |
|  |  | AP005901.1 |
|  |  | AL079303.1 |
|  |  | AL359851.1 |
|  |  | ALG1L |
|  |  | FOXP1-IT1 |
|  |  | SPDEF |
|  |  | EMX1 |
|  |  | FAM83B |
|  |  | RPL5P13 |
|  |  | DDIT4L |
|  |  | AC090578.2 |
|  |  | RPL23AP23 |
|  |  | DNAJB3 |
|  |  | RNU6-1042P |
|  |  | AL591719.1 |
|  |  | STXBP6 |
|  |  | DEFA6 |
|  |  | IRX4-AS1 |
|  |  | AC073358.1 |
|  |  | CIDEC |
|  |  | AC109583.1 |
|  |  | DHRS2 |
|  |  | RN7SL141P |
|  |  | LINC02156 |
|  |  | DCAF12L2 |
|  |  | HAS1 |
|  |  | LINC01629 |
|  |  | FOXA3 |
|  |  | AC112777.1 |
|  |  | AC002076.1 |
|  |  | HAO1 |
|  |  | PKD1L2 |
|  |  | CALML3-AS1 |
|  |  | VCAN-AS1 |
|  |  | LINC01391 |
|  |  | AL049555.1 |
|  |  | ST8SIA2 |
|  |  | CDIPTOSP |
|  |  | HOXC-AS1 |
|  |  | RN7SL736P |
|  |  | RNA5SP174 |
|  |  | PNLIPRP3 |
|  |  | AC131182.1 |
|  |  | AC016493.1 |
|  |  | SYN3-AS1 |
|  |  | IGLJCOR18 |
|  |  | AC009948.3 |
|  |  | RN7SKP106 |
|  |  | ATP5MC1P1 |
|  |  | AL353747.1 |
|  |  | CEP55 |
|  |  | AC027088.3 |
|  |  | ARHGEF3-AS1 |
|  |  | PRX |
|  |  | RN7SL543P |
|  |  | AC008115.2 |
|  |  | AC091812.1 |
|  |  | ABCA9-AS1 |
|  |  | AKR1C1 |
|  |  | RN7SL57P |
|  |  | AC026369.3 |
|  |  | RN7SL396P |
|  |  | IGSF9 |
|  |  | COX6B2 |
|  |  | IGKV6D-41 |
|  |  | LINC01665 |
|  |  | MUCL1 |
|  |  | LINC01524 |
|  |  | HOXC9 |
|  |  | C1QL4 |
|  |  | AC011700.1 |
|  |  | PODXL2 |
|  |  | AL606970.3 |
|  |  | KCNN4 |
|  |  | LINC02527 |
|  |  | AC114812.2 |
|  |  | MRGPRG-AS1 |
|  |  | FERMT1 |
|  |  | LINC00308 |
|  |  | AL161668.1 |
|  |  | C2orf69P1 |
|  |  | MAST1 |
|  |  | ISM2 |
|  |  | AL354993.2 |
|  |  | LUZP4 |
|  |  | MTATP8P1 |
|  |  | SPIC |
|  |  | AC025254.1 |
|  |  | AC002463.1 |
|  |  | CD177 |
|  |  | CACNA1C-AS4 |
|  |  | ASTN2-AS1 |
|  |  | AC005077.4 |
|  |  | MB |
|  |  | ADAM18 |
|  |  | AC078820.1 |
|  |  | LINC02105 |
|  |  | CAMK2N2 |
|  |  | PLK1 |
|  |  | RRM2 |
|  |  | AC105031.2 |
|  |  | LINC01312 |
|  |  | RPS27AP13 |
|  |  | AC091931.1 |
|  |  | ADCY8 |
|  |  | CACNA1S |
|  |  | STEAP2-AS1 |
|  |  | LSAMP-AS1 |
|  |  | TNNT1 |
|  |  | AC104024.2 |
|  |  | MIR181A1HG |
|  |  | RN7SL635P |
|  |  | XRCC2 |
|  |  | ALX1 |
|  |  | DPP10-AS1 |
|  |  | ST8SIA6 |
|  |  | KRT34 |
|  |  | NKAIN4 |
|  |  | RNA5SP202 |
|  |  | RN7SL362P |
|  |  | AC126323.3 |
|  |  | EEF1B2P8 |
|  |  | NCAPG |
|  |  | SGO1 |
|  |  | AC104257.1 |
|  |  | KRT33B |
|  |  | AL365226.1 |
|  |  | CEL |
|  |  | LINC01981 |
|  |  | SNORA2C |
|  |  | AC016868.1 |
|  |  | PPIAP45 |
|  |  | AC148477.4 |
|  |  | SNORD56B |
|  |  | MYZAP |
|  |  | KLK15 |
|  |  | CBLC |
|  |  | SNORD8 |
|  |  | IGKV1D-22 |
|  |  | AL589863.1 |
|  |  | AC079089.1 |
|  |  | AC132807.2 |
|  |  | RN7SL674P |
|  |  | ST8SIA6-AS1 |
|  |  | AC010894.2 |
|  |  | AL512638.1 |
|  |  | DPYD-AS1 |
|  |  | LINC00941 |
|  |  | AC091046.2 |
|  |  | NDP |
|  |  | AC084864.1 |
|  |  | PATE4 |
|  |  | ERVK-28 |
|  |  | AC093904.3 |
|  |  | SKA3 |
|  |  | SUMO2P8 |
|  |  | LINC02065 |
|  |  | AC084816.1 |
|  |  | BX510359.2 |
|  |  | TLX3 |
|  |  | ZBTB20-AS3 |
|  |  | DUSP5P1 |
|  |  | RN7SL272P |
|  |  | FGF11 |
|  |  | MIR4653 |
|  |  | SLCO5A1 |
|  |  | AC012317.1 |
|  |  | AC021683.2 |
|  |  | AC139792.1 |
|  |  | GYPE |
|  |  | CAVIN2 |
|  |  | LINC02813 |
|  |  | FOLR3 |
|  |  | CPNE7 |
|  |  | AC068389.3 |
|  |  | LINC01971 |
|  |  | FUT2 |
|  |  | GPRC6A |
|  |  | QRSL1P3 |
|  |  | MIR1205 |
|  |  | E2F8 |
|  |  | BIRC6-AS1 |
|  |  | SCG2 |
|  |  | FAM155B |
|  |  | PNCK |
|  |  | AC083841.2 |
|  |  | ACMSD |
|  |  | LGALS14 |
|  |  | IGKV1D-37 |
|  |  | MTCO2P19 |
|  |  | PPP1R14BP2 |
|  |  | SLC16A9 |
|  |  | ADH1B |
|  |  | CRYGN |
|  |  | FMO2 |
|  |  | SEPTIN7P12 |
|  |  | RHBDL1 |
|  |  | BRWD1P3 |
|  |  | AC092608.3 |
|  |  | AC020922.1 |
|  |  | C4BPAP2 |
|  |  | DLX2 |
|  |  | AC087667.1 |
|  |  | AL357153.4 |
|  |  | SAMSN1-AS1 |
|  |  | LINC01885 |
|  |  | AL360091.2 |
|  |  | H2AC11 |
|  |  | AQP12B |
|  |  | ADGRB1 |
|  |  | ABCB10P1 |
|  |  | ZNF723 |
|  |  | KRT78 |
|  |  | PIMREG |
|  |  | CASC8 |
|  |  | LINC00200 |
|  |  | AC244131.1 |
|  |  | AL645608.6 |
|  |  | TLE7 |
|  |  | CTSL3P |
|  |  | AP000462.1 |
|  |  | FOSB |
|  |  | CLVS2 |
|  |  | AL445223.1 |
|  |  | AC090802.1 |
|  |  | CKAP2L |
|  |  | LINC01436 |
|  |  | LGALS7B |
|  |  | CDCA3 |
|  |  | AC243972.3 |
|  |  | AC013437.1 |
|  |  | AC005996.1 |
|  |  | MIR1245A |
|  |  | AC069079.1 |
|  |  | CDC45 |
|  |  | C1orf105 |
|  |  | KCNJ13 |
|  |  | KIF20A |
|  |  | GABRP |
|  |  | MYO18B |
|  |  | AP005060.1 |
|  |  | AC112721.2 |
|  |  | DPP6 |
|  |  | AC093110.1 |
|  |  | SCGB1A1 |
|  |  | APOB |
|  |  | XAGE1B |
|  |  | AC105450.1 |
|  |  | NWD2 |
|  |  | TEK |
|  |  | LHX3 |
|  |  | SRPK3 |
|  |  | AC126323.1 |
|  |  | CERS3 |
|  |  | COL7A1 |
|  |  | AC103853.1 |
|  |  | AC061975.1 |
|  |  | AL513475.1 |
|  |  | AC005828.2 |
|  |  | NELL1 |
|  |  | LUCAT1 |
|  |  | ARL4AP1 |
|  |  | MAGEA5 |
|  |  | MACC1-AS1 |
|  |  | AC110741.1 |
|  |  | AC079416.4 |
|  |  | NEFL |
|  |  | PLEKHN1 |
|  |  | ESPL1 |
|  |  | NDC80 |
|  |  | AC044810.2 |
|  |  | AC016877.3 |
|  |  | RNU1-70P |
|  |  | MIR7-3HG |
|  |  | TMEM184A |
|  |  | LINC01561 |
|  |  | AP000897.1 |
|  |  | LRRTM1 |
|  |  | AC132186.1 |
|  |  | AL592148.2 |
|  |  | RN7SL648P |
|  |  | AP000526.1 |
|  |  | AC026320.1 |
|  |  | AC002381.1 |
|  |  | RNU1-2 |
|  |  | AL117341.1 |
|  |  | MKI67 |
|  |  | VIPR1 |
|  |  | AC021087.4 |
|  |  | AC013457.1 |
|  |  | AC002075.1 |
|  |  | KIF26B |
|  |  | KRTAP3-1 |
|  |  | AC006206.2 |
|  |  | FOXE3 |
|  |  | AC015921.1 |
|  |  | AL357054.1 |
|  |  | NKX2-3 |
|  |  | AC241520.1 |
|  |  | RAMP3 |
|  |  | SNORA22 |
|  |  | AC138904.1 |
|  |  | TTLL8 |
|  |  | TTLL11-IT1 |
|  |  | SLC29A4 |
|  |  | Z82173.1 |
|  |  | SNORA80B |
|  |  | LINC01863 |
|  |  | AL162584.1 |
|  |  | RPTN |
|  |  | SOX1-OT |
|  |  | CACNG7 |
|  |  | AL358178.1 |
|  |  | AL032819.2 |
|  |  | AP002358.2 |
|  |  | SPOCK1 |
|  |  | LINC00470 |
|  |  | MESP2 |
|  |  | GTSE1 |
|  |  | AL049874.3 |
|  |  | AC244250.1 |
|  |  | NAT16 |
|  |  | AC021218.1 |
|  |  | SPAG5 |
|  |  | STK32A-AS1 |
|  |  | SEMA3G |
|  |  | DEFB131E |
|  |  | OVAAL |
|  |  | AC134349.2 |
|  |  | LCE3D |
|  |  | IGFL1P1 |
|  |  | UNC5CL |
|  |  | AC092535.1 |
|  |  | GDF10 |
|  |  | AC108729.1 |
|  |  | NECAB1 |
|  |  | LINC02307 |
|  |  | AC012404.1 |
|  |  | NPFFR2 |
|  |  | GRIN1 |
|  |  | AC005291.2 |
|  |  | SPOCK2 |
|  |  | TFR2 |
|  |  | AC055733.2 |
|  |  | AL162413.1 |
|  |  | ADAM12 |
|  |  | LYVE1 |
|  |  | RN7SL12P |
|  |  | AL133372.2 |
|  |  | ZBTB8OSP1 |
|  |  | IGHD3-16 |
|  |  | AC243830.1 |
|  |  | ERN2 |
|  |  | C20orf197 |
|  |  | IGHG1 |
|  |  | AL161740.1 |
|  |  | AC021766.1 |
|  |  | EPN3 |
|  |  | AC009139.1 |
|  |  | BX510359.3 |
|  |  | AC073525.1 |
|  |  | PANTR1 |
|  |  | LINC02742 |
|  |  | ITIH6 |
|  |  | APOF |
|  |  | MDK |
|  |  | TSPY3 |
|  |  | AC106872.1 |
|  |  | TMPRSS15 |
|  |  | ECEL1P2 |
|  |  | AC007993.2 |
|  |  | AC005176.1 |
|  |  | UPK3A |
|  |  | LINC02294 |
|  |  | SLC7A11 |
|  |  | AL138847.2 |
|  |  | AC069431.2 |
|  |  | LINC00511 |
|  |  | AC134981.1 |
|  |  | MANCR |
|  |  | C15orf48 |
|  |  | AC027288.3 |
|  |  | TAS2R13 |
|  |  | AL136298.1 |
|  |  | STX1A |
|  |  | RN7SL430P |
|  |  | AGT |
|  |  | AC092910.1 |
|  |  | AC006148.1 |
|  |  | LINC01448 |
|  |  | THPO |
|  |  | AC007848.2 |
|  |  | APCDD1L-DT |
|  |  | SLC38A3 |
|  |  | RPS27AP8 |
|  |  | MYH2 |
|  |  | EFNA3 |
|  |  | IGKV2-29 |
|  |  | AL592043.1 |
|  |  | NEK2P4 |
|  |  | DRP2 |
|  |  | ALOX15P2 |
|  |  | GFAP |
|  |  | AC096669.1 |
|  |  | CDCA8 |
|  |  | AC007529.1 |
|  |  | LINC02312 |
|  |  | LINC01616 |
|  |  | MYOD1 |
|  |  | AP005137.2 |
|  |  | AC011611.2 |
|  |  | AC068305.2 |
|  |  | AC025434.1 |
|  |  | AC145343.1 |
|  |  | CCNB2 |
|  |  | JSRP1 |
|  |  | AL353583.1 |
|  |  | SPATA31D1 |
|  |  | AL136369.1 |
|  |  | IGHVII-78-1 |
|  |  | TATDN2P1 |
|  |  | PLA2G2C |
|  |  | ACADL |
|  |  | HMMR |
|  |  | DPRXP1 |
|  |  | IGHV3-43 |
|  |  | IFNK |
|  |  | BCAN |
|  |  | LINC01098 |
|  |  | TFAP2A-AS1 |
|  |  | CLDN2 |
|  |  | HEPHL1 |
|  |  | IGKV1OR1-1 |
|  |  | ORC6 |
|  |  | IGHV1-3 |
|  |  | AC097358.2 |
|  |  | AL157400.2 |
|  |  | TSACC |
|  |  | GINS1 |
|  |  | SCARNA23 |
|  |  | ADRB1 |
|  |  | AC092608.2 |
|  |  | AC078950.1 |
|  |  | AC110792.2 |
|  |  | HELT |
|  |  | ODAM |
|  |  | KRT79 |
|  |  | AC099313.1 |
|  |  | GALP |
|  |  | AP002453.1 |
|  |  | MIR5689HG |
|  |  | AC141273.2 |
|  |  | AC087783.1 |
|  |  | INMT-MINDY4 |
|  |  | LINC02433 |
|  |  | SKA1 |
|  |  | AC020934.1 |
|  |  | PRR32 |
|  |  | RECQL4 |
|  |  | AL390774.2 |
|  |  | DTD1-AS1 |
|  |  | AC012339.1 |
|  |  | EGLN3 |
|  |  | NQO1 |
|  |  | SNX9-AS1 |
|  |  | PERM1 |
|  |  | AC007686.1 |
|  |  | ARNTL2-AS1 |
|  |  | AC018442.2 |
|  |  | AC087612.1 |
|  |  | MSLNL |
|  |  | IGHV1OR15-2 |
|  |  | AC004080.2 |
|  |  | AD000813.1 |
|  |  | AP000842.3 |
|  |  | NCAPH |
|  |  | GCKR |
|  |  | AC090774.2 |
|  |  | OTX2-AS1 |
|  |  | NGEF |
|  |  | AC135782.1 |
|  |  | TGM5 |
|  |  | AL356311.1 |
|  |  | AC114296.1 |
|  |  | CYP4F3 |
|  |  | DIAPH1-AS1 |
|  |  | HMGB1P7 |
|  |  | SLC17A2 |
|  |  | DQX1 |
|  |  | TCF21 |
|  |  | AC106873.1 |
|  |  | AC091043.1 |
|  |  | TLCD3B |
|  |  | PCSK2 |
|  |  | PI3 |
|  |  | MAGI1-AS1 |
|  |  | IGKV3D-15 |
|  |  | LINC02827 |
|  |  | IGKV1D-16 |
|  |  | NEUROG2 |
|  |  | NBPF4 |
|  |  | AP002833.2 |
|  |  | SNORA11B |
|  |  | CYP27C1 |
|  |  | RNU4-25P |
|  |  | SHOC1 |
|  |  | RNU7-84P |
|  |  | AF127936.1 |
|  |  | SNORA26 |
|  |  | AL161938.1 |
|  |  | SLC35F4 |
|  |  | CTXND1 |
|  |  | PHACTR2-AS1 |
|  |  | RN7SL5P |
|  |  | TNNC2 |
|  |  | AC104852.1 |
|  |  | IGHV1-58 |
|  |  | FOXI1 |
|  |  | AC005550.2 |
|  |  | OR51E1 |
|  |  | AL356320.2 |
|  |  | AP002478.1 |
|  |  | IGKV1OR9-2 |
|  |  | RNU7-124P |
|  |  | TFF3 |
|  |  | SERPINA2 |
|  |  | LINC01081 |
|  |  | GPS2P2 |
|  |  | RXFP1 |
|  |  | RN7SL760P |
|  |  | MEIOB |
|  |  | IGKV1D-39 |
|  |  | LINC02495 |
|  |  | TMEM196 |
|  |  | LINC02594 |
|  |  | GABRR3 |
|  |  | NOX5 |
|  |  | AC090541.1 |
|  |  | LINC02806 |
|  |  | Z85994.1 |
|  |  | PTGES |
|  |  | GP9 |
|  |  | PKMYT1 |
|  |  | P2RY6 |
|  |  | RPS6P12 |
|  |  | AF127577.2 |
|  |  | FGFBP2 |
|  |  | AC015712.6 |
|  |  | IGLV3-12 |
|  |  | RAP2CP1 |
|  |  | CKM |
|  |  | CDCA5 |
|  |  | RNVU1-32 |
|  |  | HECW1-IT1 |
|  |  | LINC00456 |
|  |  | NIFKP3 |
|  |  | SYT16 |
|  |  | BNIP3P24 |
|  |  | AP000577.2 |
|  |  | LINC00488 |
|  |  | CCDC141 |
|  |  | SEC14L3 |
|  |  | AL096677.1 |
|  |  | AC016576.1 |
|  |  | SLC1A7 |
|  |  | CCNB1 |
|  |  | AC117465.1 |
|  |  | RSPO1 |
|  |  | LINC02167 |
|  |  | AC090709.1 |
|  |  | AC092964.1 |
|  |  | CCDC166 |
|  |  | B3GAT1-DT |
|  |  | AC025031.5 |
|  |  | ATP6V1C2 |
|  |  | ADRB2 |
|  |  | LRFN2 |
|  |  | IGHV3-64 |
|  |  | MNX1-AS2 |
|  |  | AL627308.1 |
|  |  | AC116025.2 |
|  |  | PRDM12 |
|  |  | MYT1 |
|  |  | LINC02470 |
|  |  | DNAJB6P7 |
|  |  | LINC02747 |
|  |  | AC091305.1 |
|  |  | DUX4L9 |
|  |  | IGHV1-17 |
|  |  | RN7SL554P |
|  |  | AL133243.4 |
|  |  | AC013724.1 |
|  |  | AKR7L |
|  |  | AC022905.1 |
|  |  | AC068722.1 |
|  |  | AC136603.1 |
|  |  | AC005344.1 |
|  |  | CDCA2 |
|  |  | PDHA1P1 |
|  |  | CAV3 |
|  |  | RN7SL498P |
|  |  | AC008277.1 |
|  |  | EN1 |
|  |  | FGF10-AS1 |
|  |  | AL445209.1 |
|  |  | PCDH15 |
|  |  | AC087286.1 |
|  |  | RN7SL183P |
|  |  | LINC00551 |
|  |  | AC078850.2 |
|  |  | RN7SKP103 |
|  |  | TLL2 |
|  |  | EPS15P1 |
|  |  | DES |
|  |  | AC008517.1 |
|  |  | AP001189.1 |
|  |  | RPE65 |
|  |  | LINC02282 |
|  |  | AC015923.1 |
|  |  | AC008833.1 |
|  |  | IGSF1 |
|  |  | IFNL2 |
|  |  | AC131206.1 |
|  |  | BCL2L12P1 |
|  |  | AL583808.1 |
|  |  | CYP4F11 |
|  |  | AC105150.1 |
|  |  | PROKR1 |
|  |  | CREB3L3 |
|  |  | F7 |
|  |  | LINC02477 |
|  |  | AC104088.1 |
|  |  | AC009044.1 |
|  |  | AC008496.1 |
|  |  | INSL6 |
|  |  | LINC02862 |
|  |  | DUTP7 |
|  |  | AL109615.3 |
|  |  | RGS7 |
|  |  | LCN9 |
|  |  | RN7SL711P |
|  |  | AC013286.1 |
|  |  | AC126696.3 |
|  |  | IGHV1OR21-1 |
|  |  | RN7SL493P |
|  |  | MTRF1LP2 |
|  |  | SMYD3-AS1 |
|  |  | SLC9A9-AS1 |
|  |  | IGHV1-14 |
|  |  | CLEC3A |
|  |  | AC012174.1 |
|  |  | AC105760.1 |
|  |  | KREMEN2 |
|  |  | AC008498.2 |
|  |  | GFRA3 |
|  |  | AC114812.3 |
|  |  | LINC02560 |
|  |  | AL354707.1 |
|  |  | DCAF12L1 |
|  |  | AVPR1B |
|  |  | BANF2 |
|  |  | ALKAL2 |
|  |  | AC034105.3 |
|  |  | BLACAT1 |
|  |  | TANK-AS1 |
|  |  | LINC01572 |
|  |  | FAM25A |
|  |  | WFIKKN2 |
|  |  | RN7SL313P |
|  |  | SLC7A9 |
|  |  | CHRNB2 |
|  |  | AC004986.1 |
|  |  | RET |
|  |  | SLC30A2 |
|  |  | CAMK2B |
|  |  | RN7SL475P |
|  |  | RNU6-251P |
|  |  | HBE1 |
|  |  | MIR194-2HG |
|  |  | IGHV1OR16-3 |
|  |  | CCDC169 |
|  |  | AC084026.1 |
|  |  | GTF2IP7 |
|  |  | TICRR |
|  |  | CKMT1B |
|  |  | LINC01587 |
|  |  | IGHV2OR16-5 |
|  |  | AC040963.1 |
|  |  | AC007431.1 |
|  |  | AC034105.1 |
|  |  | SCN8A |
|  |  | MARCO |
|  |  | AP000553.2 |
|  |  | NDST4 |
|  |  | AC027288.2 |
|  |  | SLC16A14 |
|  |  | FNDC1-IT1 |
|  |  | AC237221.2 |
|  |  | CDCA7 |
|  |  | MCIDAS |
|  |  | SCARNA11 |
|  |  | AC004947.1 |
|  |  | AL161431.1 |
|  |  | ASIC5 |
|  |  | GOLGA2P11 |
|  |  | PRSS38 |
|  |  | AP000619.1 |
|  |  | SPRR2F |
|  |  | LINC01192 |
|  |  | AC007448.3 |
|  |  | MGAT3 |
|  |  | AC105219.1 |
|  |  | AL008721.1 |
|  |  | CLRN3 |
|  |  | FAM178B |
|  |  | CYMP-AS1 |
|  |  | IGHV1OR15-4 |
|  |  | HGFAC |
|  |  | CCNA2 |
|  |  | PARAL1 |
|  |  | AC110921.1 |
|  |  | CDC37P1 |
|  |  | EPHA5-AS1 |
|  |  | SERPINA11 |
|  |  | AC004816.1 |
|  |  | AC021594.1 |
|  |  | GP2 |
|  |  | CNTNAP2 |
|  |  | AC061975.5 |
|  |  | AGBL1 |
|  |  | GDNF |
|  |  | PTBP1P |
|  |  | AL031736.1 |
|  |  | LINC00051 |
|  |  | RNU6-915P |
|  |  | CNGB1 |
|  |  | AL365356.1 |
|  |  | LINC01878 |
|  |  | AL512604.1 |
|  |  | AC010976.2 |
|  |  | STX11 |
|  |  | TRIM53AP |
|  |  | AC114878.2 |
|  |  | LINC02323 |
|  |  | MGC27382 |
|  |  | JPH3 |
|  |  | ACY3 |
|  |  | SPAG4 |
|  |  | MAGEA2 |
|  |  | MUC16 |
|  |  | TMEM171 |
|  |  | LINC01788 |
|  |  | THBS2 |
|  |  | CNBD1 |
|  |  | AC126323.6 |
|  |  | RPL17P17 |
|  |  | RN7SL329P |
|  |  | AL513188.1 |
|  |  | RNU6-619P |
|  |  | LINC02208 |
|  |  | BHLHA15 |
|  |  | MND1 |
|  |  | SCARNA18 |
|  |  | AOX2P |
|  |  | AGRP |
|  |  | INSRR |
|  |  | LINC02683 |
|  |  | AC004888.1 |
|  |  | GLDC |
|  |  | SLC25A39P1 |
|  |  | CLCA1 |
|  |  | AC113615.1 |
|  |  | AL133373.2 |
|  |  | RGS20 |
|  |  | IGHD3-22 |
|  |  | AC090753.1 |
|  |  | AC013410.2 |
|  |  | RNU6-122P |
|  |  | LINC01551 |
|  |  | MDGA2 |
|  |  | TSHR |
|  |  | AC020687.1 |
|  |  | AC008406.3 |
|  |  | FPR2 |
|  |  | AP005328.1 |
|  |  | LYPD4 |
|  |  | OTOR |
|  |  | COL1A1 |
|  |  | NIP7P3 |
|  |  | CDKN3 |
|  |  | IGHJ1 |
|  |  | RN7SL838P |
|  |  | ANKRD36C |
|  |  | IFNE |
|  |  | AC127029.2 |
|  |  | LINC01270 |
|  |  | AC093770.1 |
|  |  | LINC01913 |
|  |  | RSPH6A |
|  |  | OVCH2 |
|  |  | AL358232.1 |
|  |  | LINC01993 |
|  |  | AL591686.1 |
|  |  | Z84478.1 |
|  |  | PAQR9 |
|  |  | LINC01564 |
|  |  | AC103563.4 |
|  |  | RN7SL555P |
|  |  | IGHV2-70D |
|  |  | AC004221.1 |
|  |  | LINC02347 |
|  |  | SLC5A12 |
|  |  | IGHV1OR16-1 |
|  |  | AC109439.1 |
|  |  | AC099066.2 |
|  |  | PCARE |
|  |  | AC104982.2 |
|  |  | AC006504.4 |
|  |  | AC092691.1 |
|  |  | PGGT1BP2 |
|  |  | PMPCAP1 |
|  |  | HNF4A |
|  |  | FSIP2 |
|  |  | CACNG5 |
|  |  | AQP4 |
|  |  | GPR89P |
|  |  | AC141273.3 |
|  |  | PDSS1P1 |
|  |  | C12orf42 |
|  |  | AC079915.1 |
|  |  | IGHV3-33 |
|  |  | AC010719.1 |
|  |  | FGF10 |
|  |  | THEGL |
|  |  | AP005119.2 |
|  |  | CKMT1A |
|  |  | IL19 |
|  |  | H2AC20 |
|  |  | SCAT1 |
|  |  | AC108102.1 |
|  |  | BEAN1 |
|  |  | BHMT2 |
|  |  | COMP |
|  |  | AC009686.1 |
|  |  | DNAJC17P1 |
|  |  | RTN4RL2 |
|  |  | AC079866.2 |
|  |  | AC110769.3 |
|  |  | MIR124-2HG |
|  |  | AL844908.1 |
|  |  | AC112176.1 |
|  |  | OR7E104P |
|  |  | LINC01040 |
|  |  | AL022313.2 |
|  |  | AL162578.1 |
|  |  | CCL25 |
|  |  | METTL21AP1 |
|  |  | AL133153.1 |
|  |  | SPRR2G |
|  |  | AC109446.2 |
|  |  | FTCD |
|  |  | FXYD1 |
|  |  | LINC01447 |
|  |  | AC108865.1 |
|  |  | AL354824.2 |
|  |  | RN7SL30P |
|  |  | RETN |
|  |  | KLHDC7B-DT |
|  |  | VPS26BP1 |
|  |  | AL590666.3 |
|  |  | IGKV1OR22-1 |
|  |  | TMEM145 |
|  |  | AC099788.1 |
|  |  | VPS9D1-AS1 |
|  |  | AC020928.1 |
|  |  | AC011287.2 |
|  |  | RN7SL297P |
|  |  | MAST4-IT1 |
|  |  | AL136987.1 |
|  |  | AC067863.2 |
|  |  | AP000438.1 |
|  |  | TBR1 |
|  |  | ALDH3B2 |
|  |  | TMEFF1 |
|  |  | SYT2 |
|  |  | BNIP3P8 |
|  |  | IGLV5-37 |
|  |  | IGLV3-32 |
|  |  | GPR22 |
|  |  | DUSP5P2 |
|  |  | AC005291.1 |
|  |  | TMEM210 |
|  |  | GINS2 |
|  |  | ITGA11 |
|  |  | RN7SL709P |
|  |  | DDX18P5 |
|  |  | AC007240.1 |
|  |  | BLID |
|  |  | TRIM43 |
|  |  | AL596218.1 |
|  |  | AL513324.1 |
|  |  | U62317.3 |
|  |  | XKRX |
|  |  | AL359378.1 |
|  |  | AC007161.2 |
|  |  | IGKJ5 |
|  |  | GNRH2 |
|  |  | KCTD9P5 |
|  |  | TMEM88B |
|  |  | HTR2C |
|  |  | IGHV2-26 |
|  |  | F12 |
|  |  | AQP6 |
|  |  | ENSAP1 |
|  |  | AC140479.1 |
|  |  | AL390719.1 |
|  |  | RPSAP58 |
|  |  | RNA5SP323 |
|  |  | H2BW1 |
|  |  | HSPE1P13 |
|  |  | GS1-594A7.3 |
|  |  | AC010307.4 |
|  |  | TAC4 |
|  |  | SPINT5P |
|  |  | LINC01502 |
|  |  | LINC02188 |
|  |  | GPC6-AS1 |
|  |  | AC092447.1 |
|  |  | LRRC36 |
|  |  | PAX2 |
|  |  | AC087235.2 |
|  |  | AC100774.1 |
|  |  | NHS-AS1 |
|  |  | FAM189A2 |
|  |  | PCLAF |
|  |  | GLDCP1 |
|  |  | SCG5 |
|  |  | RN7SL792P |
|  |  | MELTF |
|  |  | AC007494.2 |
|  |  | AC005702.2 |
|  |  | AL354714.3 |
|  |  | SOX7 |
|  |  | AIM2 |
|  |  | HES6 |
|  |  | AC211433.1 |
|  |  | AL358333.2 |
|  |  | IGKV2D-40 |
|  |  | AC131971.1 |
|  |  | AP001970.1 |
|  |  | AC093844.1 |
|  |  | LINC02417 |
|  |  | EDDM3A |
|  |  | TMEM249 |
|  |  | PCNAP4 |
|  |  | SYNGR3 |
|  |  | AC022778.1 |
|  |  | SSX6P |
|  |  | AC024901.1 |
|  |  | VTCN1 |
|  |  | AC007001.1 |
|  |  | IGHV4-4 |
|  |  | RASA2-IT1 |
|  |  | STEAP1 |
|  |  | AF107885.2 |
|  |  | AC013410.1 |
|  |  | AC092115.3 |
|  |  | RN7SL683P |
|  |  | TLX2 |
|  |  | LINC01322 |
|  |  | ZNF648 |
|  |  | SELE |
|  |  | AC092120.2 |
|  |  | AL049830.4 |
|  |  | IGKV4-1 |
|  |  | IGKV1-37 |
|  |  | LINC00337 |
|  |  | AL031777.1 |
|  |  | AC046158.1 |
|  |  | IGLV9-49 |
|  |  | HOXD4 |
|  |  | DNASE2B |
|  |  | AL157937.1 |
|  |  | CMTM5 |
|  |  | ANKRD18B |
|  |  | SCGB1D2 |
|  |  | GJB4 |
|  |  | TOX3 |
|  |  | AL139010.1 |
|  |  | SATB2-AS1 |
|  |  | IGHGP |
|  |  | CYMP |
|  |  | AL021395.1 |
|  |  | FOXN3-AS2 |
|  |  | PSMA2P2 |
|  |  | IGKV1-39 |
|  |  | AC106772.1 |
|  |  | MCHR2 |
|  |  | IGLV1-36 |
|  |  | CREG2 |
|  |  | BACH1-IT3 |
|  |  | IGLV3-21 |
|  |  | AL390755.1 |
|  |  | AL160281.1 |
|  |  | SLC19A3 |
|  |  | RN7SL219P |
|  |  | C2orf91 |
|  |  | AP003497.2 |
|  |  | AC079316.2 |
|  |  | AC007405.2 |
|  |  | TDRD1 |
|  |  | AC010735.1 |
|  |  | HEBP2P1 |
|  |  | CDH3 |
|  |  | IGLV3-6 |
|  |  | RCOR2 |
|  |  | RNU11-2P |
|  |  | B3GALT5 |
|  |  | USP12P2 |
|  |  | PODNL1 |
|  |  | MIR8067 |
|  |  | LINC02266 |
|  |  | KRT8P38 |
|  |  | LINC01429 |
|  |  | AC007533.1 |
|  |  | AC010857.1 |
|  |  | AC090571.1 |
|  |  | CAB39P1 |
|  |  | CD36 |
|  |  | RPS15AP13 |
|  |  | CBLN1 |
|  |  | AC119744.1 |
|  |  | TNNI3 |
|  |  | RN7SL752P |
|  |  | SLC7A3 |
|  |  | LINC02331 |
|  |  | AC126323.4 |
|  |  | AL445524.1 |
|  |  | AC092800.1 |
|  |  | IGHV3-73 |
|  |  | CDKN2A-DT |
|  |  | CNGB3 |
|  |  | PLAAT1 |
|  |  | AL606534.2 |
|  |  | AL160408.1 |
|  |  | AC010609.1 |
|  |  | AC018629.1 |
|  |  | SMAD1-AS2 |
|  |  | AL513412.1 |
|  |  | CASR |
|  |  | MT1JP |
|  |  | AL645730.1 |
|  |  | AC022098.2 |
|  |  | LINC01411 |
|  |  | ANKRD22 |
|  |  | HHIP |
|  |  | AL163193.1 |
|  |  | LINC01399 |
|  |  | AC108471.1 |
|  |  | RAMP2 |
|  |  | ALG1L5P |
|  |  | RGCC |
|  |  | LINC02437 |
|  |  | AL592494.1 |
|  |  | MSL3P1 |
|  |  | AC091173.1 |
|  |  | LINC01425 |
|  |  | LEMD1 |
|  |  | AP001574.1 |
|  |  | LINC00866 |
|  |  | OR1N2 |
|  |  | AC120024.4 |
|  |  | AC012354.4 |
|  |  | ADGRE3 |
|  |  | RNY4P19 |
|  |  | SMKR1 |
|  |  | MIR576 |
|  |  | TAF1L |
|  |  | NBPF6 |
|  |  | TBC1D26-AS1 |
|  |  | PRKG2-AS1 |
|  |  | SNORA80E |
|  |  | MIR670HG |
|  |  | LINC02111 |
|  |  | RN7SL836P |
|  |  | AC011933.2 |
|  |  | IGLL1 |
|  |  | RARRES2P6 |
|  |  | TESMIN |
|  |  | MC5R |
|  |  | AC092447.7 |
|  |  | RBP2 |
|  |  | AC104461.1 |
|  |  | KU-MEL-3 |
|  |  | AC067805.1 |
|  |  | AC005808.1 |
|  |  | LINC01087 |
|  |  | CD177P1 |
|  |  | AC034223.1 |
|  |  | FO681492.1 |
|  |  | IGHV1-46 |
|  |  | GBX2 |
|  |  | PLD5 |
|  |  | RNVU1-33 |
|  |  | IGHG3 |
|  |  | SIRPB1 |
|  |  | BOLL |
|  |  | AC098592.2 |
|  |  | UNC13A |
|  |  | AL136320.1 |
|  |  | AL355916.1 |
|  |  | AC025211.1 |
|  |  | GALR3 |
|  |  | TRIM9 |
|  |  | AL391839.1 |
|  |  | EME1 |
|  |  | RNU4-24P |
|  |  | RN7SL260P |
|  |  | KIFC1 |
|  |  | IGHV1OR15-6 |
|  |  | AC104248.1 |
|  |  | IGKV3-15 |
|  |  | RN7SKP237 |
|  |  | RNU5D-1 |
|  |  | AC145146.1 |
|  |  | ORC1 |
|  |  | AL023775.1 |
|  |  | TMPOP1 |
|  |  | AC010776.2 |
|  |  | AP005131.4 |
|  |  | ABCA8 |
|  |  | CXCR1 |
|  |  | STRC |
|  |  | GPR37 |
|  |  | GCM1 |
|  |  | KPRP |
|  |  | AL079307.1 |
|  |  | ST7-OT4 |
|  |  | H2BC18 |
|  |  | FAM8A2P |
|  |  | MMP20 |
|  |  | AL627311.1 |
|  |  | NPPB |
|  |  | AC116096.1 |
|  |  | SLC38A11 |
|  |  | E2F7 |
|  |  | AC099677.3 |
|  |  | HROB |
|  |  | AC112721.1 |
|  |  | SLC14A1 |
|  |  | S1PR1 |
|  |  | AL513475.2 |
|  |  | AC104211.1 |
|  |  | RN7SL166P |
|  |  | IGKV1OR2-11 |
|  |  | CDT1 |
|  |  | CCNJP2 |
|  |  | KANK3 |
|  |  | COL6A6 |
|  |  | ASPHD1 |
|  |  | CENPI |
|  |  | PTHLH |
|  |  | IGHV7-40 |
|  |  | FAM111B |
|  |  | FCF1P8 |
|  |  | SPINK2 |
|  |  | IL1RL1 |
|  |  | LINC02454 |
|  |  | AC090796.1 |
|  |  | CHRND |
|  |  | AL133384.1 |
|  |  | UGT1A2P |
|  |  | ALOXE3 |
|  |  | IGSF10 |
|  |  | TAAR3P |
|  |  | LINC02816 |
|  |  | CLSPN |
|  |  | IGLV3-26 |
|  |  | DNAH8-AS1 |
|  |  | ZBTB20-AS1 |
|  |  | U8 |
|  |  | STRIP2 |
|  |  | ANKRD36BP2 |
|  |  | AC007490.1 |
|  |  | AC091925.2 |
|  |  | COL11A2 |
|  |  | ROBO4 |
|  |  | KLRF2 |
|  |  | IGFL4 |
|  |  | PLEK2 |
|  |  | AL033504.1 |
|  |  | AC005796.1 |
|  |  | AP003469.2 |
|  |  | RPS20P15 |
|  |  | IL6 |
|  |  | AC092834.1 |
|  |  | GFRA4 |
|  |  | MEGF10 |
|  |  | AL606517.1 |
|  |  | LINC00844 |
|  |  | IGKV1OR22-5 |
|  |  | AL022313.4 |
|  |  | TFAP2B |
|  |  | GOLGA7B |
|  |  | AC126407.1 |
|  |  | AWAT1 |
|  |  | AC115621.1 |
|  |  | KRT17 |
|  |  | AC107419.1 |
|  |  | DYNAP |
|  |  | LGR4 |
|  |  | LRRN3 |
|  |  | MIR196A1 |
|  |  | LINC02324 |
|  |  | SCN3A |
|  |  | AC008277.2 |
|  |  | GPRIN1 |
|  |  | HCN1 |
|  |  | IGHV4-34 |
|  |  | AL158801.1 |
|  |  | SFTPA1 |
|  |  | SCN4B |
|  |  | MTOR-AS1 |
|  |  | AC027514.1 |
|  |  | IGKV1OR2-9 |
|  |  | IGHV1OR15-9 |
|  |  | ARTN |
|  |  | RPL6P12 |
|  |  | AC023905.1 |
|  |  | PLCZ1 |
|  |  | AC064874.1 |
|  |  | PVT1 |
|  |  | AC060834.2 |
|  |  | FAM72C |
|  |  | AC015712.4 |
|  |  | AC022809.1 |
|  |  | CHN2-AS1 |
|  |  | AC010478.1 |
|  |  | ACTN2 |
|  |  | IGKV1-16 |
|  |  | TDRG1 |
|  |  | IGHV7-81 |
|  |  | AC008738.4 |
|  |  | AC007785.1 |
|  |  | PAX9 |
|  |  | IGKV5-2 |
|  |  | PCAT6 |
|  |  | LIMS2 |
|  |  | CCDC85A |
|  |  | AP001626.1 |
|  |  | HNF1A-AS1 |
|  |  | AL022323.1 |
|  |  | CPNE4 |
|  |  | BMS1P22 |
|  |  | AC022973.2 |
|  |  | KIF11 |
|  |  | AP001442.1 |
|  |  | ANKRD30BP3 |
|  |  | AC110760.2 |
|  |  | ANOS1 |
|  |  | IGHV1-69D |
|  |  | IGKV2-28 |
|  |  | GREB1 |
|  |  | AC005865.1 |
|  |  | CD101 |
|  |  | OR8A1 |
|  |  | NPM1P20 |
|  |  | IGKV1OR2-108 |
|  |  | AC135068.6 |
|  |  | KIF5A |
|  |  | MYH7 |
|  |  | NLRP8 |
|  |  | IGHV3-50 |
|  |  | LGI2 |
|  |  | AL136131.2 |
|  |  | RPS16P5 |
|  |  | FAM222A-AS1 |
|  |  | HTR1D |
|  |  | AL355987.5 |
|  |  | TAL1 |
|  |  | HIGD1B |
|  |  | SMPX |
|  |  | AL121956.1 |
|  |  | KIF15 |
|  |  | AC010480.1 |
|  |  | AC078962.3 |
|  |  | RXFP2 |
|  |  | AL831737.1 |
|  |  | SPRR2E |
|  |  | AL662889.1 |
|  |  | AC079597.1 |
|  |  | AC078899.4 |
|  |  | HID1-AS1 |
|  |  | FAM86LP |
|  |  | TENT5B |
|  |  | AC099342.1 |
|  |  | IL11 |
|  |  | PRAMENP |
|  |  | AC009268.1 |
|  |  | SOX21 |
|  |  | AL359853.1 |
|  |  | BRIP1 |
|  |  | IGLV4-69 |
|  |  | AL589743.3 |
|  |  | LCN2 |
|  |  | ADAM7 |
|  |  | GS1-279B7.1 |
|  |  | SETP10 |
|  |  | AL591501.1 |
|  |  | RHBDL2 |
|  |  | PRAC1 |
|  |  | RN7SL230P |
|  |  | MGAM2 |
|  |  | KRT9 |
|  |  | ACTG1P22 |
|  |  | AC116312.1 |
|  |  | SLC7A5 |
|  |  | HSPA12B |
|  |  | AC105219.3 |
|  |  | RN7SL144P |
|  |  | PAFAH1B2P1 |
|  |  | MMP17 |
|  |  | HOXC8 |
|  |  | AC127024.8 |
|  |  | AL355312.3 |
|  |  | AC055876.1 |
|  |  | MC4R |
|  |  | AC108676.1 |
|  |  | RPAP2P1 |
|  |  | AL031668.1 |
|  |  | AC011595.1 |
|  |  | RNU6-1283P |
|  |  | SMIM31 |
|  |  | FHL5 |
|  |  | SPC24 |
|  |  | IGHV1-2 |
|  |  | TAS2R63P |
|  |  | ENO1P3 |
|  |  | NR1I2 |
|  |  | LAMP3 |
|  |  | AL161729.2 |
|  |  | DDX4 |
|  |  | AC078860.1 |
|  |  | MEIOC |
|  |  | XKR7 |
|  |  | MYH16 |
|  |  | IGHV5-51 |
|  |  | MIR9-3HG |
|  |  | HNRNPA1P3 |
|  |  | RSU1P3 |
|  |  | HAL |
|  |  | CALCRL |
|  |  | USP12-AS1 |
|  |  | AC022748.2 |
|  |  | AC023090.1 |
|  |  | TBX18 |
|  |  | ABCG8 |
|  |  | LINC02260 |
|  |  | AC105914.2 |
|  |  | AC021683.1 |
|  |  | CA7 |
|  |  | BUB1 |
|  |  | AP000919.2 |
|  |  | RN7SL505P |
|  |  | CNFN |
|  |  | ACNATP |
|  |  | H2AC10P |
|  |  | OSTCP8 |
|  |  | AL357146.1 |
|  |  | CASQ2 |
|  |  | CENPE |
|  |  | AC080128.1 |
|  |  | AC105114.1 |
|  |  | ESPNP |
|  |  | CYP26A1 |
|  |  | IGKV1D-12 |
|  |  | PNMA3 |
|  |  | TK1 |
|  |  | AL031658.2 |
|  |  | ADAMTS14 |
|  |  | AC007546.2 |
|  |  | GOLM1 |
|  |  | AC053503.5 |
|  |  | CACNA1E |
|  |  | AURKA |
|  |  | ARL2BPP4 |
|  |  | ZBTB8B |
|  |  | PPP1R17 |
|  |  | LINC02015 |
|  |  | LINC02050 |
|  |  | UPK1B |
|  |  | AC079209.2 |
|  |  | LINC01348 |
|  |  | SALRNA1 |
|  |  | MT1M |
|  |  | AC016542.3 |
|  |  | C11orf53 |
|  |  | SEPTIN7P11 |
|  |  | IGKV6-21 |
|  |  | AC093890.1 |
|  |  | FOXF1 |
|  |  | PSMA1P1 |
|  |  | TNFRSF18 |
|  |  | NDUFAF4P4 |
|  |  | AL662890.1 |
|  |  | AL138759.1 |
|  |  | TRPM5 |
|  |  | IGHJ3P |
|  |  | AC138409.1 |
|  |  | AC021237.1 |
|  |  | F11 |
|  |  | IGKV1-5 |
|  |  | TNMD |
|  |  | EMCN |
|  |  | DCST1 |
|  |  | AL109761.1 |
|  |  | LINC01812 |
|  |  | AC084026.2 |
|  |  | GPR52 |
|  |  | AC006946.2 |
|  |  | LNX1-AS1 |
|  |  | C18orf15 |
|  |  | IFNL3 |
|  |  | AC114763.2 |
|  |  | AP000688.2 |
|  |  | AC026992.2 |
|  |  | AP000619.3 |
|  |  | NMNAT2 |
|  |  | FAM9B |
|  |  | IGKV1OR2-6 |
|  |  | ACRV1 |
|  |  | CLPS |
|  |  | PLAC9 |
|  |  | WASF1P1 |
|  |  | LILRP2 |
|  |  | DIRC1 |
|  |  | AL772337.2 |
|  |  | KRT15 |
|  |  | AC013391.3 |
|  |  | H3C13 |
|  |  | ESCO2 |
|  |  | LINC00319 |
|  |  | AC141930.1 |
|  |  | FLJ16779 |
|  |  | EPHX4 |
|  |  | AC009365.3 |
|  |  | IGKV1OR2-3 |
|  |  | AC010608.2 |
|  |  | H2BC5 |
|  |  | EMP2 |
|  |  | AKAIN1 |
|  |  | KNL1 |
|  |  | CFHR5 |
|  |  | H3C10 |
|  |  | RN7SL390P |
|  |  | QPCT |
|  |  | IFNL4 |
|  |  | NDUFB4P2 |
|  |  | RMDN2-AS1 |
|  |  | MOCS1P1 |
|  |  | AC007494.1 |
|  |  | AC009303.3 |
|  |  | DLX5 |
|  |  | PROM2 |
|  |  | IGLV6-57 |
|  |  | GRIK2 |
|  |  | KCNQ5 |
|  |  | IGKV1D-17 |
|  |  | ZNF90P1 |
|  |  | AP001429.1 |
|  |  | RN7SKP180 |
|  |  | AC096577.1 |
|  |  | AC136424.2 |
|  |  | CR382285.1 |
|  |  | AGMAT |
|  |  | GPA33 |
|  |  | PLA2G4F |
|  |  | SYT5 |
|  |  | GAPDHP49 |
|  |  | SPAAR |
|  |  | AC103563.1 |
|  |  | RPS3AP28 |
|  |  | SLCO1A2 |
|  |  | AC018647.1 |
|  |  | EDN3 |
|  |  | LINC01475 |
|  |  | AC132825.4 |
|  |  | LHCGR |
|  |  | IGKC |
|  |  | ZWINT |
|  |  | AL807761.4 |
|  |  | LINC02443 |
|  |  | MAMDC2 |
|  |  | TNXB |
|  |  | IGLV1-41 |
|  |  | DUXAP10 |
|  |  | RPL35AP13 |
|  |  | AC008870.3 |
|  |  | AC092535.2 |
|  |  | CPB2 |
|  |  | SOX17 |
|  |  | CLDN5 |
|  |  | AP003696.1 |
|  |  | ADAMTS19 |
|  |  | AC023794.2 |
|  |  | AC063926.1 |
|  |  | AP002001.1 |
|  |  | C10orf99 |
|  |  | AP004290.1 |
|  |  | IGHG2 |
|  |  | SGK2 |
|  |  | AC012363.1 |
|  |  | CACNA1B |
|  |  | AC025031.4 |
|  |  | IGHV2-5 |
|  |  | STYK1 |
|  |  | ADGRE1 |
|  |  | MRPS31P2 |
|  |  | CBX5P1 |
|  |  | MASP1 |
|  |  | AL133373.1 |
|  |  | IGHJ2 |
|  |  | VAC14-AS1 |
|  |  | RN7SL575P |
|  |  | RN7SL128P |
|  |  | RAPGEF4-AS1 |
|  |  | SCAND3P1 |
|  |  | UCN3 |
|  |  | CDO1 |
|  |  | DIRAS1 |
|  |  | NECAB2 |
|  |  | METTL7B |
|  |  | EPAS1 |
|  |  | KIF23 |
|  |  | AL590004.3 |
|  |  | SERPINB13 |
|  |  | AC124069.1 |
|  |  | LINC02362 |
|  |  | AL139002.1 |
|  |  | UNC5D |
|  |  | RBM22P4 |
|  |  | AL713899.1 |
|  |  | AC069542.1 |
|  |  | WDR72 |
|  |  | AC112236.1 |
|  |  | PLAU |
|  |  | AK4 |
|  |  | LINC01357 |
|  |  | CCDC150 |
|  |  | SYT6 |
|  |  | RNU6-608P |
|  |  | LINC01793 |
|  |  | AP003385.4 |
|  |  | SMILR |
|  |  | AK4P3 |
|  |  | LINC01968 |
|  |  | AC073569.1 |
|  |  | RN7SL569P |
|  |  | ZP2 |
|  |  | AC111000.4 |
|  |  | RNU7-79P |
|  |  | IGKV3OR22-2 |
|  |  | IGHV1-69 |
|  |  | PATE2 |
|  |  | AC103409.1 |
|  |  | SLC6A17 |
|  |  | THAP12P3 |
|  |  | AC009135.2 |
|  |  | MIR3189 |
|  |  | AC087521.1 |
|  |  | RN7SL356P |
|  |  | HMGB1P23 |
|  |  | IMPDH1P8 |
|  |  | EREG |
|  |  | LINC01819 |
|  |  | ZBTB46-AS1 |
|  |  | HOXB1 |
|  |  | OR9K1P |
|  |  | BTBD17 |
|  |  | AC002511.2 |
|  |  | CAPNS2 |
|  |  | AC112206.2 |
|  |  | NMUR1 |
|  |  | NOTO |
|  |  | AC093817.1 |
|  |  | FIRRE |
|  |  | LIPM |
|  |  | AL451054.1 |
|  |  | SRARP |
|  |  | RN7SL778P |
|  |  | IGKV3OR2-268 |
|  |  | MRPL15P1 |
|  |  | PRR20G |
|  |  | IGHV3-76 |
|  |  | LINC01645 |
|  |  | IGKV2OR22-4 |
|  |  | MUCL3 |
|  |  | CST11 |
|  |  | CCDC177 |
|  |  | LINC02804 |
|  |  | IGLV3-9 |
|  |  | PRKCG |
|  |  | AP001207.2 |
|  |  | NUSAP1 |
|  |  | NSA2P3 |
|  |  | AC022929.2 |
|  |  | RN7SL395P |
|  |  | PLAC9P1 |
|  |  | AC005064.1 |
|  |  | RPL23AP30 |
|  |  | AC106882.1 |
|  |  | MIR148A |
|  |  | LINC01096 |
|  |  | AC072039.1 |
|  |  | RPL35AP23 |
|  |  | AC024581.1 |
|  |  | MYO3B-AS1 |
|  |  | STIL |
|  |  | AC092903.1 |
|  |  | SAMMSON |
|  |  | ARHGEF26 |
|  |  | SBK1 |
|  |  | AC092881.1 |
|  |  | CHP1P1 |
|  |  | AL390718.1 |
|  |  | MAD2L1 |
|  |  | AC144450.1 |
|  |  | LINC01271 |
|  |  | FZD9 |
|  |  | MFAP4 |
|  |  | ODAPH |
|  |  | FSD1 |
|  |  | CAMP |
|  |  | SNORA80D |
|  |  | AMER3 |
|  |  | SYNPO2L |
|  |  | LINC00705 |
|  |  | RFLNA |
|  |  | SFTPA2 |
|  |  | XIAPP2 |
|  |  | CBX3P3 |
|  |  | SFRP5 |
|  |  | LINC02825 |
|  |  | IGLV3-19 |
|  |  | GCOM1 |
|  |  | AC131649.2 |
|  |  | FSTL4 |
|  |  | MTFR2P1 |
|  |  | AOC3 |
|  |  | DSCR10 |
|  |  | IGHV1-67 |
|  |  | IL20RB-AS1 |
|  |  | IBSP |
|  |  | LINC02321 |
|  |  | AC007182.1 |
|  |  | SALL4P7 |
|  |  | AC027018.1 |
|  |  | DCC |
|  |  | SNX2P1 |
|  |  | LINC01580 |
|  |  | AP001642.1 |
|  |  | AC022028.2 |
|  |  | EZH2 |
|  |  | TBC1D3P1 |
|  |  | AC009646.2 |
|  |  | KRT38 |
|  |  | CHL1-AS1 |
|  |  | TUBB1 |
|  |  | VEPH1 |
|  |  | IGHV3-36 |
|  |  | AC025575.1 |
|  |  | SLC6A13 |
|  |  | SNORD67 |
|  |  | TRIP13 |
|  |  | C8orf17 |
|  |  | MTATP6P19 |
|  |  | AC012506.3 |
|  |  | IGHV3OR16-7 |
|  |  | AC006262.4 |
|  |  | C2CD4D |
|  |  | RNU4ATAC16P |
|  |  | ELK2BP |
|  |  | ASH1L-IT1 |
|  |  | FA2H |
|  |  | MAJIN |
|  |  | KCNA7 |
|  |  | IGHVII-60-1 |
|  |  | LINC01224 |
|  |  | AL138962.1 |
|  |  | LINC01697 |
|  |  | DUXAP8 |
|  |  | RLN3 |
|  |  | AP006333.2 |
|  |  | RNF225 |
|  |  | CARM1P1 |
|  |  | EEF1A1P32 |
|  |  | AC063952.1 |
|  |  | RNF222 |
|  |  | ABCC3 |
|  |  | FBXO47 |
|  |  | IGHV3-15 |
|  |  | PRC1 |
|  |  | DDN-AS1 |
|  |  | ADH4 |
|  |  | GSDMC |
|  |  | AL450344.2 |
|  |  | PCNPP3 |
|  |  | AL512324.1 |
|  |  | IGF2-AS |
|  |  | SULF1 |
|  |  | BRD7P3 |
|  |  | ASPA |
|  |  | MCF2 |
|  |  | AC026368.1 |
|  |  | NDUFB4P1 |
|  |  | OR51B5 |
|  |  | IGHV3OR16-10 |
|  |  | FAM238B |
|  |  | C14orf132 |
|  |  | PRKG2 |
|  |  | CYP4A22-AS1 |
|  |  | ENO3 |
|  |  | RNU4ATAC |
|  |  | AC113404.1 |
|  |  | IGHV3-23 |
|  |  | TMPRSS11GP |
|  |  | AC243964.2 |
|  |  | AL078587.1 |
|  |  | FCRL5 |
|  |  | IGLV3-10 |
|  |  | AC068189.1 |
|  |  | AC018892.3 |
|  |  | TAAR1 |
|  |  | LINC01873 |
|  |  | IGLV4-60 |
|  |  | SNORD94 |
|  |  | AC119751.4 |
|  |  | C8B |
|  |  | NKPD1 |
|  |  | AC084819.1 |
|  |  | TRHDE |
|  |  | AKR1D1 |
|  |  | RN7SL43P |
|  |  | FAHD2P1 |
|  |  | PRSS46P |
|  |  | ST13P20 |
|  |  | AC103563.3 |
|  |  | RN7SL173P |
|  |  | LINC01910 |
|  |  | AC015522.1 |
|  |  | RSPO2 |
|  |  | ANKRD30BL |
|  |  | SPTBN2 |
|  |  | GRIN2D |
|  |  | IGKV3D-25 |
|  |  | AL117190.1 |
|  |  | NEFH |
|  |  | MC3R |
|  |  | C20orf202 |
|  |  | AC009248.2 |
|  |  | ST7-AS2 |
|  |  | AP001318.3 |
|  |  | AC092608.1 |
|  |  | AC092828.1 |
|  |  | AC097537.1 |
|  |  | AGGF1P1 |
|  |  | AC007161.1 |
|  |  | AL807757.1 |
|  |  | SHISA9 |
|  |  | LINC01701 |
|  |  | MZT1P1 |
|  |  | AC005828.1 |
|  |  | CECR7 |
|  |  | ZG16B |
|  |  | DANT1 |
|  |  | AC069499.2 |
|  |  | IGKV2D-29 |
|  |  | IGHVII-51-2 |
|  |  | AL022314.1 |
|  |  | AC011483.1 |
|  |  | LINC01143 |
|  |  | AC134312.5 |
|  |  | DEPDC7 |
|  |  | IGLV1-40 |
|  |  | AC104984.5 |
|  |  | GSC |
|  |  | EDDM3B |
|  |  | AC108693.1 |
|  |  | IGKV2OR22-3 |
|  |  | AC079316.1 |
|  |  | SNORD38D |
|  |  | IGHV3-79 |
|  |  | NPR1 |
|  |  | IGHV1-18 |
|  |  | LDB2 |
|  |  | ACOT12 |
|  |  | RPL26P9 |
|  |  | COL3A1 |
|  |  | CDH12 |
|  |  | GPR78 |
|  |  | IQSEC3 |
|  |  | TGFBR3L |
|  |  | NPTX2 |
|  |  | IGHV1OR16-4 |
|  |  | AC004884.2 |
|  |  | IGHV4-61 |
|  |  | SNORA47 |
|  |  | GDPD2 |
|  |  | LINC02241 |
|  |  | DOCK4-AS1 |
|  |  | HMGA1P1 |
|  |  | LINGO1 |
|  |  | AC020663.2 |
|  |  | ARNTL2 |
|  |  | C14orf39 |
|  |  | PTPRB |
|  |  | PAX3 |
|  |  | AGR2 |
|  |  | LINC01426 |
|  |  | AC008456.1 |
|  |  | B4GALNT1 |
|  |  | AC007599.2 |
|  |  | IGLV2-33 |
|  |  | CRTAC1 |
|  |  | VDAC1P4 |
|  |  | PTPN2P1 |
|  |  | SINHCAFP3 |
|  |  | CBX2 |
|  |  | IGKV1-9 |
|  |  | BNIP3P10 |
|  |  | GTSF1L |
|  |  | JPH4 |
|  |  | RHOXF2 |
|  |  | AC011511.5 |
|  |  | AC135166.1 |
|  |  | H2AW |
|  |  | IGHV3-52 |
|  |  | MKRN3 |
|  |  | LINC01344 |
|  |  | CCDC154 |
|  |  | HCAR1 |
|  |  | SLC17A9 |
|  |  | CXCR2 |
|  |  | SNORC |
|  |  | ARHGAP26-AS1 |
|  |  | AP001547.1 |
|  |  | AC010998.1 |
|  |  | IGHV3-53 |
|  |  | LINC02468 |
|  |  | AC083973.1 |
|  |  | AC127164.1 |
|  |  | RIMBP2 |
|  |  | AC133540.1 |
|  |  | LRRC26 |
|  |  | AC068580.2 |
|  |  | CTSLP2 |
|  |  | IGHV3-35 |
|  |  | IGHV2-70 |
|  |  | ELK2AP |
|  |  | TMEM215 |
|  |  | AL355994.2 |
|  |  | IL17REL |
|  |  | SPRED3 |
|  |  | AC004817.4 |
|  |  | TRPA1 |
|  |  | EFCC1 |
|  |  | AL136018.1 |
|  |  | IGKV3-11 |
|  |  | LINC02842 |
|  |  | RPL31P58 |
|  |  | AF131216.3 |
|  |  | GLRA4 |
|  |  | IGDCC3 |
|  |  | AC011939.2 |
|  |  | LARP1P1 |
|  |  | AC013467.1 |
|  |  | SI |
|  |  | TMCO2 |
|  |  | AC104958.1 |
|  |  | H1-6 |
|  |  | LARGE2 |
|  |  | PCA3 |
|  |  | GPR35 |
|  |  | AP000997.2 |
|  |  | TMBIM7P |
|  |  | AL358216.1 |
|  |  | HAGLROS |
|  |  | AWAT2 |
|  |  | GJA3 |
|  |  | AP001207.1 |
|  |  | AL355607.2 |
|  |  | PRDX2P4 |
|  |  | TMEM156 |
|  |  | AC036222.1 |
|  |  | AC006273.1 |
|  |  | IL36A |
|  |  | IGKV1OR-3 |
|  |  | AL845331.2 |
|  |  | PACSIN1 |
|  |  | UPK1A |
|  |  | NCAN |
|  |  | LINC00440 |
|  |  | CATSPERD |
|  |  | TGFBR3 |
|  |  | MTCO2P33 |
|  |  | RNU6-789P |
|  |  | AC017037.3 |
|  |  | DEFA4 |
|  |  | AC139792.2 |
|  |  | FCRL4 |
|  |  | AC099329.2 |
|  |  | AC006262.1 |
|  |  | AC007285.1 |
|  |  | AL162411.1 |
|  |  | SERPINB7 |
|  |  | IGKV7-3 |
|  |  | EGOT |
|  |  | IFNL4P1 |
|  |  | MUC4 |
|  |  | BCO1 |
|  |  | AP003031.1 |
|  |  | PI15 |
|  |  | ACVRL1 |
|  |  | SRMS |
|  |  | AC092198.1 |
|  |  | AC016866.2 |
|  |  | SCG5-AS1 |
|  |  | AP000851.2 |
|  |  | MOGAT1 |
|  |  | SMAD6 |
|  |  | UNC45B |
|  |  | IGHV3OR16-16 |
|  |  | IGHV3-11 |
|  |  | RNU2-63P |
|  |  | LDC1P |
|  |  | PANX2 |
|  |  | GALNTL5 |
|  |  | KRT80 |
|  |  | HBEGF |
|  |  | AC019211.1 |
|  |  | CDK1 |
|  |  | AL596442.1 |
|  |  | IGHV3-42 |
|  |  | KRT6C |
|  |  | DUOX1 |
|  |  | OR2W6P |
|  |  | AC019270.1 |
|  |  | CDA |
|  |  | AL049830.3 |
|  |  | AC009303.2 |
|  |  | SEMA6A |
|  |  | OLR1 |
|  |  | AC140658.5 |
|  |  | DEFB4B |
|  |  | IGLV1-50 |
|  |  | IGHV3-71 |
|  |  | LINC02836 |
|  |  | RPL37P18 |
|  |  | IGHV3OR15-7 |
|  |  | MROH6 |
|  |  | CLEC2L |
|  |  | TRIM10 |
|  |  | ZNF859P |
|  |  | AC004925.1 |
|  |  | TPH2 |
|  |  | KCNT2 |
|  |  | TYMSOS |
|  |  | SMCO2 |
|  |  | LINC02735 |
|  |  | H4C8 |
|  |  | AC004034.1 |
|  |  | IGLV5-48 |
|  |  | RN7SL706P |
|  |  | PEBP4 |
|  |  | SNORA71C |
|  |  | AC108865.2 |
|  |  | EIF2S2P5 |
|  |  | ARL9 |
|  |  | LINC01985 |
|  |  | COX5BP3 |
|  |  | CACYBPP1 |
|  |  | AC135068.1 |
|  |  | SLC5A9 |
|  |  | SLC44A5 |
|  |  | IDH2-DT |
|  |  | HNRNPRP1 |
|  |  | MIR4284 |
|  |  | ECRG4 |
|  |  | SLC22A18AS |
|  |  | AC099811.1 |
|  |  | ARHGEF39 |
|  |  | LINC01607 |
|  |  | ACHE |
|  |  | LINC02817 |
|  |  | IGHD2-15 |
|  |  | AC048383.1 |
|  |  | MIR4300HG |
|  |  | FGF20 |
|  |  | RPL23AP40 |
|  |  | AC007496.3 |
|  |  | LYPD9P |
|  |  | LINC01303 |
|  |  | TWIST1 |
|  |  | RPL23AP11 |
|  |  | CDH5 |
|  |  | DUSP4 |
|  |  | KRT8P17 |
|  |  | AC106053.1 |
|  |  | AL121952.1 |
|  |  | MYADML2 |
|  |  | IGKV3D-11 |
|  |  | PRKCE-AS1 |
|  |  | AC108058.1 |
|  |  | AC130686.1 |
|  |  | IGHV3OR16-13 |
|  |  | RBMXP1 |
|  |  | LINC01744 |
|  |  | FP236383.3 |
|  |  | PLA2G4A |
|  |  | LINC02043 |
|  |  | CILP |
|  |  | AC233280.2 |
|  |  | IGHV3OR16-8 |
|  |  | GRHL3-AS1 |
|  |  | AL353572.1 |
|  |  | AL157838.1 |
|  |  | PKP4-AS1 |
|  |  | GLDN |
|  |  | PHC2-AS1 |
|  |  | LINC00958 |
|  |  | KRT23 |
|  |  | TAF9BP1 |
|  |  | LIPN |
|  |  | AC010547.2 |
|  |  | RN7SL37P |
|  |  | RN7SL471P |
|  |  | SEMA5A |
|  |  | IGHD3-3 |
|  |  | IGHV7-27 |
|  |  | IGHV3-75 |
|  |  | PLPP2 |
|  |  | TPTE2 |
|  |  | FAM83F |
|  |  | MIR559 |
|  |  | AC024940.1 |
|  |  | GBX1 |
|  |  | AC023154.1 |
|  |  | TTYH1 |
|  |  | RN7SKP116 |
|  |  | SNORA28 |
|  |  | AC003986.2 |
|  |  | CBLIF |
|  |  | GSTA3 |
|  |  | DDX11-AS1 |
|  |  | GINS4 |
|  |  | ACTN3 |
|  |  | LINC02300 |
|  |  | IGKV3-7 |
|  |  | AC245041.2 |
|  |  | KNOP1P4 |
|  |  | AL096711.2 |
|  |  | IGHV3OR16-9 |
|  |  | AL354861.2 |
|  |  | AP001885.1 |
|  |  | AC004672.1 |
|  |  | LINC02343 |
|  |  | GPR19 |
|  |  | PTGES2-AS1 |
|  |  | ATP5PBP6 |
|  |  | IGLV2-28 |
|  |  | SLC9A3 |
|  |  | LCN12 |
|  |  | IGHV4-39 |
|  |  | IGHV3-20 |
|  |  | VWC2 |
|  |  | AL355483.2 |
|  |  | IGLL5 |
|  |  | IGLV2-8 |
|  |  | KRT37 |
|  |  | SYT15 |
|  |  | RNASE10 |
|  |  | CYP4B1 |
|  |  | HSPB3 |
|  |  | LINC00624 |
|  |  | AC084880.3 |
|  |  | PHYHIPL |
|  |  | LIN7A |
|  |  | DKK1 |
|  |  | AC005476.1 |
|  |  | AC025048.1 |
|  |  | AC036111.1 |
|  |  | AC024592.2 |
|  |  | CCL23 |
|  |  | JAM2 |
|  |  | TCEAL2 |
|  |  | NPM1P47 |
|  |  | AL354943.1 |
|  |  | AL133153.2 |
|  |  | AC113146.1 |
|  |  | AC024560.1 |
|  |  | LINC00165 |
|  |  | NAV2-AS2 |
|  |  | CCM2L |
|  |  | IGHV4OR15-8 |
|  |  | AL135960.1 |
|  |  | AC018529.1 |
|  |  | AL354824.1 |
|  |  | CASP12 |
|  |  | RTRAFP1 |
|  |  | IGLV2-14 |
|  |  | ABI3BP |
|  |  | SLC2A5 |
|  |  | LINC02106 |
|  |  | TAS2R46 |
|  |  | ASNSP3 |
|  |  | DPP10 |
|  |  | RN7SL346P |
|  |  | AL109741.1 |
|  |  | SMG6-IT1 |
|  |  | AL513164.1 |
|  |  | RN7SL513P |
|  |  | AC022784.6 |
|  |  | PCAT18 |
|  |  | IGLV3-30 |
|  |  | STEAP1B |
|  |  | RN7SL218P |
|  |  | ZG16 |
|  |  | AC011473.4 |
|  |  | DIO2-AS1 |
|  |  | TMPRSS11F |
|  |  | TRIM67 |
|  |  | AC015909.1 |
|  |  | AC008060.4 |
|  |  | AC019171.1 |
|  |  | H4C12 |
|  |  | PADI4 |
|  |  | UBTFL8 |
|  |  | IGHV3-48 |
|  |  | IGKV1-17 |
|  |  | C21orf91-OT1 |
|  |  | C11orf97 |
|  |  | HHATL |
|  |  | AC007431.3 |
|  |  | NPAS3 |
|  |  | AL359551.1 |
|  |  | AL109914.1 |
|  |  | FGF12-AS2 |
|  |  | AC145207.9 |
|  |  | TUBA5P |
|  |  | AL353803.1 |
|  |  | AC122710.2 |
|  |  | AC100782.1 |
|  |  | FNDC1 |
|  |  | PNMA6E |
|  |  | GRIK4 |
|  |  | RNU6-1161P |
|  |  | IGLV5-45 |
|  |  | AC092851.1 |
|  |  | OR7E36P |
|  |  | POM121L2 |
|  |  | AL139393.1 |
|  |  | PSMC1P11 |
|  |  | LINC01979 |
|  |  | AC024451.1 |
|  |  | AC098869.1 |
|  |  | NUP210L |
|  |  | IGHV3OR16-17 |
|  |  | AC087821.1 |
|  |  | BOLA2P3 |
|  |  | TCF24 |
|  |  | AC134312.1 |
|  |  | AC023824.3 |
|  |  | ADGRF1 |
|  |  | STAR |
|  |  | SLIT2 |
|  |  | ARHGAP23P1 |
|  |  | PELATON |
|  |  | AL049874.1 |
|  |  | AC025030.1 |
|  |  | DDX12P |
|  |  | IGHV3-66 |
|  |  | HBG2 |
|  |  | RGS9 |
|  |  | IGKV1OR9-1 |
|  |  | AC092675.1 |
|  |  | AC073323.1 |
|  |  | AC008083.2 |
|  |  | AC013652.1 |
|  |  | MIR624 |
|  |  | AC008937.2 |
|  |  | HOXC6 |
|  |  | AC009275.1 |
|  |  | FGF12-AS3 |
|  |  | GJB5 |
|  |  | OR51E2 |
|  |  | AC108159.1 |
|  |  | Z86062.1 |
|  |  | UBE2QL1 |
|  |  | SLC39A8 |
|  |  | ABCC11 |
|  |  | OSBPL10-AS1 |
|  |  | CENPU |
|  |  | RN7SL321P |
|  |  | SERPINA5 |
|  |  | AL731563.1 |
|  |  | IGLV3-1 |
|  |  | ZNF402P |
|  |  | PPFIA4 |
|  |  | AL355336.1 |
|  |  | GRK5 |
|  |  | AP001189.3 |
|  |  | DUXAP9 |
|  |  | PTCHD3P2 |
|  |  | SPC25 |
|  |  | OIP5 |
|  |  | AC025166.1 |
|  |  | AC092447.5 |
|  |  | AC023825.1 |
|  |  | AL512324.3 |
|  |  | LRRC66 |
|  |  | AC108860.1 |
|  |  | BTF3P12 |
|  |  | DACH1 |
|  |  | ANGPTL5 |
|  |  | ESPN |
|  |  | AC084880.1 |
|  |  | HSPB6 |
|  |  | DBX1 |
|  |  | AC015983.1 |
|  |  | AC244153.1 |
|  |  | MME |
|  |  | F5 |
|  |  | AC136424.1 |
|  |  | PHF5GP |
|  |  | AC142381.1 |
|  |  | AC126773.5 |
|  |  | AP002754.1 |
|  |  | IGHV3OR16-6 |
|  |  | ASF1B |
|  |  | MIR561 |
|  |  | NT5C1A |
|  |  | MTND4P20 |
|  |  | BCHE |
|  |  | LINC02533 |
|  |  | MPC1L |
|  |  | SIX1 |
|  |  | NFIA-AS1 |
|  |  | SLC22A11 |
|  |  | AC145676.1 |
|  |  | HELLS |
|  |  | AMBN |
|  |  | CAPZA3 |
|  |  | DAZL |
|  |  | AC004491.1 |
|  |  | MESP1 |
|  |  | STUM |
|  |  | RPL23AP20 |
|  |  | EIF4BP5 |
|  |  | RPS15AP29 |
|  |  | AC098868.1 |
|  |  | DIO2 |
|  |  | AC005183.1 |
|  |  | SLC9A2 |
|  |  | IGLV3-15 |
|  |  | ATP5MC1P6 |
|  |  | PRH2 |
|  |  | GSTM5 |
|  |  | IGHV3-62 |
|  |  | FGFR4 |
|  |  | IGHV3-41 |
|  |  | TMEM78 |
|  |  | AC092332.1 |
|  |  | LINC01204 |
|  |  | LINC01765 |
|  |  | DOCK9-AS1 |
|  |  | AIRN |
|  |  | LINC02008 |
|  |  | AC138965.2 |
|  |  | LRRC2 |
|  |  | AC099850.3 |
|  |  | IGFBPL1 |
|  |  | CCL24 |
|  |  | AC100801.1 |
|  |  | HPCA |
|  |  | AC024475.1 |
|  |  | NECTIN4 |
|  |  | HECW1 |
|  |  | PTPRK-AS1 |
|  |  | GIMAP8 |
|  |  | ATCAY |
|  |  | WFDC10B |
|  |  | HOATZ |
|  |  | LCN6 |
|  |  | CDC25A |
|  |  | LINC01506 |
|  |  | DLGAP1-AS5 |
|  |  | SRMP2 |
|  |  | RTP1 |
|  |  | LINC00609 |
|  |  | SLC8A1-AS1 |
|  |  | WDR62 |
|  |  | FAM71F1 |
|  |  | IGHV3-65 |
|  |  | AP006259.1 |
|  |  | AUNIP |
|  |  | AC104687.2 |
|  |  | IGLV3-27 |
|  |  | LPO |
|  |  | AL356805.1 |
|  |  | DDN |
|  |  | IGLV3-17 |
|  |  | AC006262.2 |
|  |  | IGHV8-51-1 |
|  |  | PIF1 |
|  |  | AC233280.1 |
|  |  | GPR12 |
|  |  | LINC00706 |
|  |  | LINC01703 |
|  |  | E2F2 |
|  |  | STX18-IT1 |
|  |  | IGHV3-30 |
|  |  | SCUBE3 |
|  |  | SLC2A1-AS1 |
|  |  | P2RY4 |
|  |  | AC236972.3 |
|  |  | DCST2 |
|  |  | PLPPR5 |
|  |  | AC093903.1 |
|  |  | POLE2 |
|  |  | ARHGEF15 |
|  |  | GGT4P |
|  |  | PTCRA |
|  |  | AC079866.1 |
|  |  | TRIM31-AS1 |
|  |  | AC006141.1 |
|  |  | PTPN21 |
|  |  | AC009656.1 |
|  |  | IQCN |
|  |  | AC003958.2 |
|  |  | CCL14 |
|  |  | LINC01936 |
|  |  | ELOA2 |
|  |  | ATP11A-AS1 |
|  |  | FHL2 |
|  |  | MTHFD1P1 |
|  |  | C1QTNF7 |
|  |  | SIGLEC11 |
|  |  | MARK3P1 |
|  |  | AC090164.2 |
|  |  | AL162595.1 |
|  |  | TEKT5 |
|  |  | AC134879.2 |
|  |  | IGLON5 |
|  |  | AC148477.2 |
|  |  | LINC00891 |
|  |  | AL365338.1 |
|  |  | CYP27B1 |
|  |  | SP5 |
|  |  | KCNJ6 |
|  |  | TRIM40 |
|  |  | ARHGAP26-IT1 |
|  |  | C8A |
|  |  | THBS4 |
|  |  | GSG1L |
|  |  | BMP8A |
|  |  | AL035401.1 |
|  |  | RPL23AP36 |
|  |  | PKNOX2 |
|  |  | AC092685.1 |
|  |  | IGKV3D-20 |
|  |  | PTTG1 |
|  |  | ANXA8L1 |
|  |  | CALML6 |
|  |  | CT55 |
|  |  | HCN2 |
|  |  | AL109924.1 |
|  |  | AC099811.4 |
|  |  | CYP2C19 |
|  |  | PCAT7 |
|  |  | CNGA1 |
|  |  | SEL1L2 |
|  |  | SH2D5 |
|  |  | TPI1P3 |
|  |  | SLIT3 |
|  |  | SLC27A6 |
|  |  | AC068724.1 |
|  |  | FRMD3 |
|  |  | AC083967.1 |
|  |  | OVOL1 |
|  |  | COL9A1 |
|  |  | NKX6-1 |
|  |  | AC112493.1 |
|  |  | MUC20 |
|  |  | AL512283.1 |
|  |  | FUT3 |
|  |  | RNF14P1 |
|  |  | AL360091.3 |
|  |  | TMPRSS6 |
|  |  | TGM1 |
|  |  | AC022080.2 |
|  |  | HIGD1AP17 |
|  |  | PCAT19 |
|  |  | RAC3 |
|  |  | MIR27A |
|  |  | MYO16-AS1 |
|  |  | SLITRK2 |
|  |  | AC129926.1 |
|  |  | AC245517.5 |
|  |  | AC021517.1 |
|  |  | CNIH3-AS2 |
|  |  | FOXN4 |
|  |  | IGLV1-47 |
|  |  | AC022201.1 |
|  |  | ACKR4 |
|  |  | AC000061.1 |
|  |  | AC089999.2 |
|  |  | PHLDA2 |
|  |  | THBD |
|  |  | RPL39L |
|  |  | NXPH3 |
|  |  | CNTFR |
|  |  | C1orf87 |
|  |  | AP000424.2 |
|  |  | TUBB4A |
|  |  | AC013652.2 |
|  |  | IGKV1OR-2 |
|  |  | IGLJ1 |
|  |  | NOVA2 |
|  |  | AC105206.1 |
|  |  | IGHV3-49 |
|  |  | LINC00485 |
|  |  | IMPA1P1 |
|  |  | FAM230G |
|  |  | PPFIA1P1 |
|  |  | TIMM8AP1 |
|  |  | FBXO43 |
|  |  | DAO |
|  |  | C9orf24 |
|  |  | GAL3ST2 |
|  |  | RAET1K |
|  |  | HMGB1P51 |
|  |  | Z99129.3 |
|  |  | HEMGN |
|  |  | GLYATL1P4 |
|  |  | NEUROG3 |
|  |  | LINC02128 |
|  |  | DUOXA2 |
|  |  | KCTD4 |
|  |  | MIR133A1HG |
|  |  | RPL36AP33 |
|  |  | AC090018.1 |
|  |  | LINC01165 |
|  |  | LINC00519 |
|  |  | BTBD16 |
|  |  | RNA5SP282 |
|  |  | LINC01443 |
|  |  | MTCYBP3 |
|  |  | HPSE2 |
|  |  | PRDM7 |
|  |  | IGLV2-23 |
|  |  | LINC02873 |
|  |  | IGHV3-25 |
|  |  | AP000974.1 |
|  |  | LINC01593 |
|  |  | AL031599.1 |
|  |  | IGHV4-31 |
|  |  | RNU7-75P |
|  |  | CXCL2 |
|  |  | ZNF444P1 |
|  |  | MSI1 |
|  |  | IGKV2D-26 |
|  |  | AC027088.4 |
|  |  | CLLU1-AS1 |
|  |  | SRPX2 |
|  |  | AC093281.1 |
|  |  | AC120036.3 |
|  |  | HMGA1 |
|  |  | KIAA0408 |
|  |  | AL138880.1 |
|  |  | AP003351.1 |
|  |  | Z99943.1 |
|  |  | CCNYL2 |
|  |  | AP000769.1 |
|  |  | AC053503.1 |
|  |  | AC087473.1 |
|  |  | AC138393.2 |
|  |  | AK4P1 |
|  |  | AL732366.2 |
|  |  | MIR3677 |
|  |  | LINC02463 |
|  |  | NRG3 |
|  |  | AC012370.1 |
|  |  | IGHV4-28 |
|  |  | SCN7A |
|  |  | CHST4 |
|  |  | OR9A4 |
|  |  | AC008568.1 |
|  |  | AP001025.1 |
|  |  | AC011287.1 |
|  |  | PDK4 |
|  |  | AP000977.1 |
|  |  | TMEM74 |
|  |  | PCSK1N |
|  |  | G6PC2 |
|  |  | ANGPTL1 |
|  |  | PRSS48 |
|  |  | AC109446.3 |
|  |  | SHCBP1 |
|  |  | FAM72B |
|  |  | AP003064.2 |
|  |  | AP003472.1 |
|  |  | AC126696.2 |
|  |  | LINC00665 |
|  |  | TENM1 |
|  |  | RAB1AP1 |
|  |  | AC005330.1 |
|  |  | RN7SKP257 |
|  |  | IGHV3-6 |
|  |  | AC022616.1 |
|  |  | IGHV1-12 |
|  |  | SRRM3 |
|  |  | MGAT3-AS1 |
|  |  | AP001528.2 |
|  |  | AC012640.1 |
|  |  | LEMD1-AS1 |
|  |  | AL355076.1 |
|  |  | WTAPP1 |
|  |  | ADAMTS16 |
|  |  | UMOD |
|  |  | AC005972.2 |
|  |  | OACYLP |
|  |  | AC067930.4 |
|  |  | EXD1 |
|  |  | AL031258.1 |
|  |  | TDRD12 |
|  |  | FFAR4 |
|  |  | VSIG4 |
|  |  | SNX18P7 |
|  |  | TRPC7-AS1 |
|  |  | ZNF114-AS1 |
|  |  | GAP43 |
|  |  | RMI2 |
|  |  | CACNA2D2 |
|  |  | HSPA8P19 |
|  |  | IGLCOR22-1 |
|  |  | AC007389.2 |
|  |  | AC100757.2 |
|  |  | PTPRN2-AS1 |
|  |  | COQ10BP2 |
|  |  | AC016251.1 |
|  |  | AC131391.1 |
|  |  | AC009032.1 |
|  |  | IQANK1 |
|  |  | FAM163A |
|  |  | IL1RL2 |
|  |  | AC046195.1 |
|  |  | IGKV2OR2-1 |
|  |  | LINC02576 |
|  |  | IFITM5 |
|  |  | KRTAP29-1 |
|  |  | AGTR2 |
|  |  | COL9A3 |
|  |  | LRRC15 |
|  |  | AFF3 |
|  |  | IGLC3 |
|  |  | C1QTNF6 |
|  |  | ACKR1 |
|  |  | AP005059.2 |
|  |  | IGHV4-55 |
|  |  | FAM72D |
|  |  | AC008883.1 |
|  |  | BCAS1 |
|  |  | ADAM28 |
|  |  | AC106876.1 |
|  |  | AC016559.3 |
|  |  | AC131097.1 |
|  |  | SPATA31C2 |
|  |  | RNVU1-22 |
|  |  | AC009884.2 |
|  |  | AC091868.2 |
|  |  | IGKV1-27 |
|  |  | CD52 |
|  |  | FAM83C-AS1 |
|  |  | KCNE4 |
|  |  | OPRM1 |
|  |  | C1orf220 |
|  |  | RN7SL160P |
|  |  | ECSCR |
|  |  | AC097639.2 |
|  |  | AC008147.4 |
|  |  | FAM216B |
|  |  | LINC01876 |
|  |  | PRR11 |
|  |  | RN7SL660P |
|  |  | SYNDIG1L |
|  |  | SFXN4P1 |
|  |  | MIR6835 |
|  |  | AF131215.1 |
|  |  | SPATA3-AS1 |
|  |  | LINC00857 |
|  |  | AC008649.1 |
|  |  | CSRNP1 |
|  |  | ARHGAP6 |
|  |  | LINC00698 |
|  |  | LINC01775 |
|  |  | LINC01731 |
|  |  | AC013549.3 |
|  |  | OPRD1 |
|  |  | CTSV |
|  |  | AC120036.4 |
|  |  | C15orf54 |
|  |  | AC112196.1 |
|  |  | AC072062.1 |
|  |  | RN7SKP299 |
|  |  | EPHB2 |
|  |  | FAM83D |
|  |  | RN7SKP230 |
|  |  | IGKV1OR10-1 |
|  |  | TH |
|  |  | GABRB1 |
|  |  | OR10G2 |
|  |  | AC092612.1 |
|  |  | GCLC |
|  |  | SNORD46 |
|  |  | AL445423.1 |
|  |  | RPSAP13 |
|  |  | ADAMTS9-AS1 |
|  |  | PKP1 |
|  |  | AC099494.3 |
|  |  | C12orf40 |
|  |  | S100A3 |
|  |  | CYP1D1P |
|  |  | LINC02228 |
|  |  | SELENOV |
|  |  | AC010424.1 |
|  |  | TAS2R64P |
|  |  | FOXO6 |
|  |  | AC068831.4 |
|  |  | ADGRD2 |
|  |  | LINC01168 |
|  |  | IGKV1OR2-118 |
|  |  | AC243967.1 |
|  |  | AC107959.3 |
|  |  | LRRC32 |
|  |  | ADGRG4 |
|  |  | AC005722.4 |
|  |  | CST2 |
|  |  | HSPD1P11 |
|  |  | AC130404.1 |
|  |  | B3GAT1 |
|  |  | MMRN1 |
|  |  | SVOP |
|  |  | NKX6-3 |
|  |  | AL136221.1 |
|  |  | MROH9 |
|  |  | AC131902.2 |
|  |  | ZBED2 |
|  |  | AC108174.1 |
|  |  | AC068305.1 |
|  |  | AC027243.1 |
|  |  | LINC01166 |
|  |  | CFD |
|  |  | KLK13 |
|  |  | DIPK1C |
|  |  | RNASEH1P1 |
|  |  | BEND3P2 |
|  |  | IGHV1-68 |
|  |  | PRR13P1 |
|  |  | IGHV1OR15-1 |
|  |  | RUNX3-AS1 |
|  |  | AL731563.2 |
|  |  | MIR34B |
|  |  | PCOLCE2 |
|  |  | AC018797.2 |
|  |  | AC002351.1 |
|  |  | AC025470.2 |
|  |  | RNA5SP29 |
|  |  | KPNA7 |
|  |  | AL035409.1 |
|  |  | AC020910.4 |
|  |  | FAP |
|  |  | H2BC11 |
|  |  | U52111.1 |
|  |  | HHLA2 |
|  |  | AL121832.3 |
|  |  | RXRG |
|  |  | SIX6 |
|  |  | FAM242F |
|  |  | SLC22A8 |
|  |  | ZDHHC4P1 |
|  |  | ST18 |
|  |  | FBN2 |
|  |  | U47924.1 |
|  |  | LINC01356 |
|  |  | AC107886.1 |
|  |  | AC092349.1 |
|  |  | ODF3L1 |
|  |  | TESC-AS1 |
|  |  | EGF |
|  |  | AC106827.1 |
|  |  | TAMALIN |
|  |  | IGKV1D-8 |
|  |  | STPG4 |
|  |  | REEP6 |
|  |  | TAS1R3 |
|  |  | MRGPRX3 |
|  |  | FOXD1 |
|  |  | AL358394.1 |
|  |  | ANKRD66 |
|  |  | WWC2 |
|  |  | AC011239.2 |
|  |  | AC008443.2 |
|  |  | CKS1BP2 |
|  |  | AL353807.2 |
|  |  | AC002398.2 |
|  |  | IGKV2-18 |
|  |  | AC008554.1 |
|  |  | JAKMIP3 |
|  |  | TVP23CP1 |
|  |  | IGHV3-21 |
|  |  | GYG2 |
|  |  | DOK5 |
|  |  | AL512593.1 |
|  |  | IGKV3-20 |
|  |  | EPHX3 |
|  |  | AL589765.6 |
|  |  | FCGR3B |
|  |  | CES5AP1 |
|  |  | AP000997.3 |
|  |  | AL135902.1 |
|  |  | LINC02725 |
|  |  | AC091805.1 |
|  |  | KC877392.1 |
|  |  | AC137834.2 |
|  |  | CHEK1 |
|  |  | AGXT2 |
|  |  | CYCSP4 |
|  |  | RNY4P7 |
|  |  | RUFY4 |
|  |  | MROH2A |
|  |  | IGHV3-19 |
|  |  | AC064807.2 |
|  |  | ADAMTS1 |
|  |  | HAR1B |
|  |  | CATSPERB |
|  |  | FCF1P5 |
|  |  | H4C14 |
|  |  | HMGB2P1 |
|  |  | C1QL1 |
|  |  | AP003168.1 |
|  |  | DNA2 |
|  |  | CR545473.1 |
|  |  | KDELR3 |
|  |  | SOX21-AS1 |
|  |  | CAV2 |
|  |  | RPL9P30 |
|  |  | Z84485.1 |
|  |  | AP003086.2 |
|  |  | P4HA3 |
|  |  | AC091814.1 |
|  |  | MRC1 |
|  |  | AP005264.4 |
|  |  | AL356273.1 |
|  |  | AC008680.1 |
|  |  | RNVU1-29 |
|  |  | AC026992.1 |
|  |  | ALPK2 |
|  |  | WDR86 |
|  |  | BNIP3P25 |
|  |  | GGT8P |
|  |  | TBC1D26 |
|  |  | STPG3 |
|  |  | PAFAH1B3 |
|  |  | HSF2BP |
|  |  | RNU6-917P |
|  |  | AC068700.1 |
|  |  | G3BP1P1 |
|  |  | PFKP-DT |
|  |  | AL137818.1 |
|  |  | MAGEB17 |
|  |  | ASIC4-AS1 |
|  |  | IGLJ3 |
|  |  | OTP |
|  |  | GGTLC3 |
|  |  | SLC26A4 |
|  |  | AF106564.1 |
|  |  | LINC01843 |
|  |  | ADAMDEC1 |
|  |  | AP003419.1 |
|  |  | LINC00513 |
|  |  | AC009806.1 |
|  |  | AL355304.1 |
|  |  | FAM110D |
|  |  | INSYN2A |
|  |  | AC023481.1 |
|  |  | HIF1A-AS3 |
|  |  | SCG3 |
|  |  | AC021127.1 |
|  |  | SCARA5 |
|  |  | CENPK |
|  |  | C2CD6 |
|  |  | H2BC4 |
|  |  | AL133492.1 |
|  |  | AL354861.3 |
|  |  | AL513485.1 |
|  |  | CR589904.1 |
|  |  | MCM4 |
|  |  | Z93022.1 |
|  |  | LINC01615 |
|  |  | SELP |
|  |  | AP005131.3 |
|  |  | AL353753.1 |
|  |  | RN7SL402P |
|  |  | LINC01205 |
|  |  | IGKV2D-30 |
|  |  | USHBP1 |
|  |  | BDNF |
|  |  | VWF |
|  |  | AC022973.4 |
|  |  | RSPO4 |
|  |  | DISP3 |
|  |  | EZHIP |
|  |  | RN7SL67P |
|  |  | AF131215.2 |
|  |  | IGLV2-5 |
|  |  | AC010168.1 |
|  |  | AC103957.1 |
|  |  | LINC01250 |
|  |  | GRIA4 |
|  |  | KCND2 |
|  |  | DTNB-AS1 |
|  |  | BNIP3P40 |
|  |  | MSR1 |
|  |  | DERL3 |
|  |  | CLEC1A |
|  |  | LINC02562 |
|  |  | ATP5PBP3 |
|  |  | AC026725.1 |
|  |  | AC128709.2 |
|  |  | AC099518.1 |
|  |  | AL391421.1 |
|  |  | PRSS3P1 |
|  |  | RNFT2 |
|  |  | LINC02771 |
|  |  | HABP2 |
|  |  | PECAM1 |
|  |  | MEI4 |
|  |  | AL158801.3 |
|  |  | SLC25A10 |
|  |  | RNF113B |
|  |  | KL |
|  |  | AC092143.1 |
|  |  | AC117500.1 |
|  |  | AC068643.1 |
|  |  | AC007608.2 |
|  |  | PIMREGP4 |
|  |  | AC105046.1 |
|  |  | DSP |
|  |  | APOBEC3B |
|  |  | AC092573.1 |
|  |  | INAVA |
|  |  | TMEM105 |
|  |  | RAD51 |
|  |  | GALR2 |
|  |  | H2BC16P |
|  |  | C17orf78 |
|  |  | CHRDL1 |
|  |  | SLC28A1 |
|  |  | AC104791.2 |
|  |  | RNU2-6P |
|  |  | CTXN3 |
|  |  | FAM162B |
|  |  | AC018816.2 |
|  |  | AHNAK2 |
|  |  | LINC01352 |
|  |  | AP001011.1 |
|  |  | AL354993.1 |
|  |  | AC010974.1 |
|  |  | CMTM2 |
|  |  | RUNDC3A |
|  |  | CLDN9 |
|  |  | LUARIS |
|  |  | C4BPB |
|  |  | PGM5-AS1 |
|  |  | RNU6-548P |
|  |  | RERG-AS1 |
|  |  | GUCY1B2 |
|  |  | SLC16A8 |
|  |  | AC004947.2 |
|  |  | SNHG4 |
|  |  | KRT40 |
|  |  | SPN |
|  |  | SCARNA9 |
|  |  | DKK2 |
|  |  | LINC01845 |
|  |  | AC090116.1 |
|  |  | RASIP1 |
|  |  | AL359094.1 |
|  |  | BCAS2P1 |
|  |  | AC008063.1 |
|  |  | ADAMTSL3 |
|  |  | RAMP1 |
|  |  | KLK3 |
|  |  | AC009121.2 |
|  |  | KCNA5 |
|  |  | SLURP1 |
|  |  | AC009951.4 |
|  |  | SYN2 |
|  |  | RN7SL363P |
|  |  | AC009630.2 |
|  |  | NME1 |
|  |  | DGAT2L6 |
|  |  | CLEC14A |
|  |  | PROX1-AS1 |
|  |  | ANGPT1 |
|  |  | POLD2P1 |
|  |  | RNA5SP33 |
|  |  | KCNK2 |
|  |  | GPR146 |
|  |  | ATP1A2 |
|  |  | HBD |
|  |  | AC040904.1 |
|  |  | OCIAD2 |
|  |  | SLC25A24P1 |
|  |  | MTDHP4 |
|  |  | EYS |
|  |  | AL161618.1 |
|  |  | SH2D3C |
|  |  | AL096865.1 |
|  |  | TUBB4BP2 |
|  |  | SPATS1 |
|  |  | BET1P1 |
|  |  | RPL7P42 |
|  |  | IGKV2D-18 |
|  |  | AC023347.1 |
|  |  | RN7SKP80 |
|  |  | C10orf71-AS1 |
|  |  | SBSPON |
|  |  | AC005230.1 |
|  |  | AC020658.4 |
|  |  | LINC01624 |
|  |  | AC092168.2 |
|  |  | ANKRD34C |
|  |  | AL035410.1 |
|  |  | PHACTR1 |
|  |  | AC092436.1 |
|  |  | AL353746.1 |
|  |  | AL022341.2 |
|  |  | FRG2C |
|  |  | SULT1C4 |
|  |  | PTPRD-AS1 |
|  |  | HLX-AS1 |
|  |  | LINC00867 |
|  |  | EFNA4 |
|  |  | LINC02316 |
|  |  | RNU6-998P |
|  |  | LINC02880 |
|  |  | AL121772.2 |
|  |  | AC019193.2 |
|  |  | Z94160.1 |
|  |  | AC022336.3 |
|  |  | COX4I2 |
|  |  | RNU6-711P |
|  |  | AC104984.3 |
|  |  | TRGJP2 |
|  |  | LINC01583 |
|  |  | AP006248.3 |
|  |  | AL133480.1 |
|  |  | EPHA5 |
|  |  | AL391840.1 |
