# Supplementary material for: Identification of Differentially Expressed and Prognostic lncRNAs for the Construction of ceRNA Networks in Lung Adenocarcinoma
Source: J Oncol. 2021 Dec 27;2021:2659550. doi: 10.1155/2021/2659550 (PMC8723861; doi:10.1155/2021/2659550)
Supplement: Supplementary Materials — Supplementary Figure S1. Analysis of network topology for soft thresholding powers (weighting coefficient, β) in lncRNAs, miRNAs, and mRNAs. The x-axis represented different soft thresholding powers. Upper: assessment for R2 of log(k) and log(p(k)) correlation coefficients corresponding to different β values in the network. The red line indicated a scale-free topology fitting index R2 of 0.9. Lower: analysis of the mean connectivity for various β values. Supplementary Figure S2. Correlation analysis of lncRNAs, miRNAs, and mRNAs in the ceRNA network. (a) LncRNAs were negatively correlated with miRNAs. (b) Negative correlation among mRNAs and miRNAs. (c) LncRNAs had a positive correlation with mRNAs. Supplementary Figure S3. ROC plots of the prognostic lncRNA signature in the TCGA-LUAD dataset. (a) Survival-dependent ROC curves attested the prognostic significance of DElncRNAs. The area under the red line represented the 1-year AUC; the area under the blue line represented the 3-year AUC; and the area under the black line represented the 5-year AUC. (b) ROC curve analysis showed the application value of DElncRNAs in the diagnosis. Supplementary Table S1. The summarized data of DERNAs. Supplementary Table S2. The integrated results of DERNAs via differential expression analysis, WGCNA, and website prediction. Supplementary Table S3. Baseline clinicopathological characteristics of the TCGA-LUAD cohort. (n = 513). Supplementary Table S4. Univariate Cox regression analysis of factors associated with overall survival in the TCGA-LUAD dataset. Supplementary Table S5. Multivariable Cox regression analysis of factors associated with overall survival in the TCGA-LUAD dataset. Supplementary Table S6. 6 prognostic lncRNA-mediated ceRNA networks. [file 2659550.f1.zip › 2659550.f1/Supplementary Table S3..doc]

| Supplementary Table S3. Baseline clinicopathological characteristics of the TCGA-LUAD cohort. (n=513). | | | | | | | | | | | |
| --- | --- | --- | --- | --- | --- | --- | --- | --- | --- | --- | --- |
| Samples | OS status | OS time | RFS status | RFS time | Age | Gender | Smoking history | pT stage | pN stage | pM stage | pTNM stage |
| TCGA-05-4244 | NA | NA | NA | NA | 70 | MALE | Yes | T2 | N2 | M1 | Stage IV |
| TCGA-05-4249 | 0 | 1523 | 0 | 1523 | 67 | MALE | Yes | T2 | N0 | M0 | Stage IB |
| TCGA-05-4250 | 1 | 121 | NA | NA | 79 | FEMALE | Yes | T3 | N1 | M0 | Stage IIIA |
| TCGA-05-4382 | 0 | 607 | 1 | 334 | 68 | MALE | Yes | T2 | N0 | M0 | Stage IB |
| TCGA-05-4384 | 0 | 426 | 1 | 183 | 66 | MALE | Yes | T2 | N2 | M0 | Stage IIIA |
| TCGA-05-4389 | 0 | 1369 | 0 | 1369 | 70 | MALE | Yes | T1 | N0 | M0 | Stage IA |
| TCGA-05-4390 | 0 | 1126 | 1 | 395 | 58 | FEMALE | Yes | T2 | N0 | M0 | Stage IB |
| TCGA-05-4395 | 1 | 0 | NA | NA | 76 | MALE | Yes | T4 | N2 | M0 | Stage IIIB |
| TCGA-05-4396 | 1 | 303 | NA | NA | 76 | MALE | Yes | T4 | N1 | M0 | Stage IIIB |
| TCGA-05-4397 | 1 | 731 | NA | NA | 65 | MALE | Yes | T2 | N1 | M0 | Stage IIB |
| TCGA-05-4398 | 0 | 1431 | 0 | 1431 | 47 | FEMALE | Yes | T4 | N3 | M0 | Stage IIIB |
| TCGA-05-4402 | 1 | 244 | 0 | 244 | 57 | FEMALE | No | T2 | NX | M1 | Stage IV |
| TCGA-05-4403 | 0 | 578 | NA | NA | 76 | MALE | Yes | T2 | N0 | M0 | Stage IB |
| TCGA-05-4405 | 0 | 610 | 0 | 610 | 74 | FEMALE | Yes | T2 | N0 | M0 | Stage IB |
| TCGA-05-4410 | NA | NA | NA | NA | 62 | MALE | Yes | T2 | N0 | M0 | Stage IB |
| TCGA-05-4415 | 1 | 91 | 1 | 60 | 57 | MALE | Yes | T4 | N2 | M0 | Stage IIIB |
| TCGA-05-4417 | 0 | 455 | 0 | 455 | 51 | FEMALE | Yes | T2 | N0 | M0 | Stage IB |
| TCGA-05-4418 | 1 | 274 | NA | NA | 69 | MALE | Yes | T3 | N2 | M0 | Stage IIIA |
| TCGA-05-4420 | 0 | 912 | 0 | 912 | 41 | MALE | Yes | T2 | N0 | M0 | Stage IB |
| TCGA-05-4422 | 0 | 365 | NA | NA | 68 | MALE | Yes | T2 | N0 | M0 | Stage IB |
| TCGA-05-4424 | 0 | 913 | 1 | 153 | 70 | MALE | Yes | T3 | N0 | M0 | Stage IIB |
| TCGA-05-4425 | 0 | 669 | NA | NA | 70 | FEMALE | Yes | T2 | N0 | M1 | Stage IV |
| TCGA-05-4426 | 0 | 791 | 1 | 457 | 71 | MALE | Yes | T2 | N0 | M0 | Stage IB |
| TCGA-05-4427 | 0 | 791 | 0 | 791 | 65 | FEMALE | Yes | T2 | N1 | M0 | Stage IIB |
| TCGA-05-4430 | 0 | 761 | 0 | 761 | 59 | FEMALE | Yes | T2 | N0 | M0 | Stage IB |
| TCGA-05-4432 | 0 | 761 | 0 | 761 | 66 | MALE | Yes | T2 | N1 | M0 | Stage IIB |
| TCGA-05-4433 | 0 | 730 | 0 | 730 | 82 | MALE | Yes | T2 | N0 | M0 | Stage IB |
| TCGA-05-4434 | 1 | 457 | NA | NA | 67 | FEMALE | Yes | T4 | N1 | M1 | Stage IV |
| TCGA-05-5420 | 0 | 457 | 1 | 245 | 67 | MALE | Yes | T2 | N0 | MX | Stage IB |
| TCGA-05-5423 | 0 | 151 | NA | NA | 65 | MALE | Yes | T2 | N1 | M0 | Stage IIB |
| TCGA-05-5425 | 0 | 882 | 1 | 486 | 68 | MALE | Yes | T2b | N1 | M0 | Stage IIB |
| TCGA-05-5428 | 0 | 670 | 0 | 670 | 57 | MALE | Yes | T1b | N1 | M0 | Stage IIA |
| TCGA-05-5429 | 1 | 275 | NA | NA | 60 | MALE | No | T3 | N2 | M0 | Stage IIIA |
| TCGA-05-5715 | 0 | 62 | NA | NA | 69 | FEMALE | No | T2a | N0 | M0 | Stage IB |
| TCGA-35-3615 | 0 | 14 | NA | NA | 57 | MALE | No | T2 | N0 | M0 | Stage IB |
| TCGA-35-4122 | 0 | 225 | NA | NA | 69 | MALE | Yes | T1 | N0 | M0 | Stage IA |
| TCGA-35-4123 | 0 | 182 | NA | NA | 38 | MALE | Yes | T1 | N0 | M0 | Stage IA |
| TCGA-35-5375 | 0 | 264 | NA | NA | 61 | MALE | Yes | T2 | N2 | M0 | Stage IIIA |
| TCGA-38-4625 | 0 | 2973 | NA | NA | 66 | FEMALE | Yes | T2 | N2 | MX | Stage IIIA |
| TCGA-38-4626 | 0 | 3674 | 1 | 2518 | 57 | MALE | Yes | T2b | N0 | M0 | Stage IIA |
| TCGA-38-4627 | 1 | 1147 | NA | NA | 64 | FEMALE | Yes | T2 | NX | MX | Stage IB |
| TCGA-38-4628 | 1 | 1492 | 1 | 1083 | 65 | FEMALE | No | T2 | N1 | M0 | Stage IIB |
| TCGA-38-4629 | 1 | 864 | 1 | 379 | 68 | MALE | Yes | T1 | N0 | M0 | Stage IA |
| TCGA-38-4630 | 1 | 1073 | 1 | 836 | 75 | FEMALE | No | T2 | N2 | MX | Stage IIIA |
| TCGA-38-4631 | 1 | 354 | NA | NA | 72 | FEMALE | Yes | T1 | NX | 0 | Stage IA |
| TCGA-38-4632 | 1 | 1357 | 1 | 925 | 42 | FEMALE | Yes | T2 | N1 | 1 | Stage IIB |
| TCGA-38-6178 | 0 | 448 | 0 | 448 | 70 | FEMALE | No | T2b | N2 | NA | Stage IIIA |
| TCGA-38-7271 | 1 | 800 | 1 | 633 | 72 | FEMALE | Yes | T1 | N0 | 0 | Stage IA |
| TCGA-38-A44F | 0 | 133 | 0 | 133 | 80 | MALE | Yes | T2a | N0 | 0 | Stage IB |
| TCGA-44-2655 | 0 | 1324 | 1 | 1009 | 65 | MALE | Yes | T2a | N0 | 0 | Stage IB |
| TCGA-44-2656 | 0 | 1429 | 1 | 568 | 59 | MALE | Yes | T2 | N1 | 0 | Stage IIB |
| TCGA-44-2657 | 0 | 1351 | 0 | 1351 | 74 | FEMALE | Yes | T1b | N1 | 0 | Stage IIA |
| TCGA-44-2659 | 0 | 1367 | 0 | 1367 | 65 | FEMALE | Yes | T2 | N0 | 0 | Stage IB |
| TCGA-44-2661 | 0 | 1159 | 0 | 1159 | 69 | MALE | No | T2 | N0 | 0 | Stage IB |
| TCGA-44-2662 | 0 | 1280 | 0 | 1280 | 65 | MALE | Yes | T2 | N2 | 0 | Stage IIIA |
| TCGA-44-2665 | 0 | 1301 | 0 | 1301 | 55 | FEMALE | No | T1 | N0 | 0 | Stage IA |
| TCGA-44-2666 | 1 | 97 | 0 | 97 | 43 | MALE | Yes | T2 | N0 | 0 | Stage IB |
| TCGA-44-2668 | 1 | 761 | 1 | 433 | 51 | FEMALE | Yes | T2a | N0 | 0 | Stage IB |
| TCGA-44-3396 | 0 | 1130 | 0 | 1130 | 74 | FEMALE | Yes | T1 | N0 | 0 | Stage IA |
| TCGA-44-3398 | 0 | 1163 | 0 | 1163 | 77 | MALE | Yes | T1b | N0 | 0 | Stage IA |
| TCGA-44-3917 | 0 | 1183 | 0 | 1183 | 33 | FEMALE | Yes | T2 | N0 | 0 | Stage IB |
| TCGA-44-3918 | 0 | 1036 | 1 | 511 | 60 | MALE | Yes | T2a | N1 | 0 | Stage IIA |
| TCGA-44-3919 | 1 | 1026 | 1 | 984 | 71 | MALE | No | T2a | N0 | 0 | Stage IB |
| TCGA-44-4112 | 1 | 808 | 0 | 808 | 60 | FEMALE | Yes | T1 | N0 | 0 | Stage IA |
| TCGA-44-5643 | 0 | 1013 | 0 | 1013 | 53 | FEMALE | Yes | T2a | N0 | 0 | Stage IB |
| TCGA-44-5644 | 0 | 863 | 0 | 863 | 51 | FEMALE | Yes | T2a | N0 | NA | Stage IB |
| TCGA-44-5645 | 0 | 852 | 0 | 852 | 61 | FEMALE | Yes | T1 | N2 | NA | Stage IIIA |
| TCGA-44-6145 | 0 | 595 | 0 | 595 | 62 | FEMALE | Yes | T3 | N1 | 0 | Stage IIIA |
| TCGA-44-6146 | 0 | 728 | 1 | 631 | 64 | FEMALE | Yes | T2 | N0 | 0 | Stage IB |
| TCGA-44-6147 | 0 | 845 | 0 | 845 | 67 | FEMALE | Yes | T1 | N0 | 0 | Stage IA |
| TCGA-44-6148 | 0 | 704 | 0 | 704 | 60 | FEMALE | Yes | T2 | N1 | 0 | Stage IIB |
| TCGA-44-6774 | 0 | 658 | 0 | 658 | 56 | FEMALE | Yes | T1 | N2 | 0 | Stage IIIA |
| TCGA-44-6775 | 0 | 705 | 1 | 684 | 72 | FEMALE | Yes | T2a | N0 | NA | Stage IB |
| TCGA-44-6776 | 0 | 2616 | 0 | 2616 | 60 | FEMALE | Yes | T1b | N0 | NA | Stage IA |
| TCGA-44-6777 | 1 | 987 | 0 | 987 | 85 | FEMALE | Yes | T2 | N0 | NA | Stage IB |
| TCGA-44-6778 | 0 | 1864 | 0 | 1864 | 59 | MALE | Yes | T2 | N1 | NA | Stage IIB |
| TCGA-44-6779 | 1 | 500 | 1 | 232 | 50 | FEMALE | Yes | T2 | N1 | NA | Stage IIB |
| TCGA-44-7659 | 0 | 691 | 0 | 691 | 70 | MALE | Yes | T2a | N0 | NA | Stage IB |
| TCGA-44-7660 | 0 | 592 | 0 | 592 | 72 | FEMALE | Yes | T2 | N1 | NA | Stage IIB |
| TCGA-44-7661 | 1 | 557 | 1 | 335 | 69 | FEMALE | Yes | T2 | N1 | 0 | Stage IIB |
| TCGA-44-7662 | 0 | 218 | 0 | 218 | 61 | FEMALE | Yes | T2 | N1 | NA | Stage IIB |
| TCGA-44-7667 | 0 | 1097 | 0 | 1097 | 49 | FEMALE | Yes | T2 | NX | NA | Stage IB |
| TCGA-44-7669 | 1 | 574 | 1 | 525 | 59 | FEMALE | Yes | T2 | N1 | NA | Stage IIB |
| TCGA-44-7670 | 0 | 882 | 0 | 882 | 47 | MALE | Yes | T2 | N2 | 0 | Stage IIIA |
| TCGA-44-7671 | 0 | 889 | 1 | 883 | 64 | FEMALE | Yes | T2 | N1 | 0 | Stage IIB |
| TCGA-44-7672 | 0 | 719 | 0 | 719 | 52 | FEMALE | Yes | T2 | N0 | 0 | Stage IB |
| TCGA-44-8117 | 0 | 385 | 0 | 385 | 54 | FEMALE | Yes | T2a | N0 | 0 | Stage IB |
| TCGA-44-8119 | 0 | 285 | 0 | 285 | 73 | MALE | Yes | T3 | N0 | 0 | Stage IIB |
| TCGA-44-8120 | 0 | 260 | 0 | 260 | 58 | MALE | Yes | T2a | N0 | 0 | Stage IB |
| TCGA-44-A479 | 0 | 486 | 1 | 437 | 73 | FEMALE | Yes | T2 | N0 | NA | Stage IB |
| TCGA-44-A47A | 0 | 466 | 1 | 397 | 78 | FEMALE | Yes | T2a | N0 | NA | Stage IB |
| TCGA-44-A47B | 0 | 287 | 0 | 287 | 79 | MALE | Yes | T2a | N0 | 0 | Stage IB |
| TCGA-44-A47G | 0 | 351 | 0 | 351 | 73 | FEMALE | Yes | T1 | N0 | 0 | Stage IA |
| TCGA-44-A4SS | 0 | 415 | 0 | 415 | 73 | MALE | Yes | T1b | N0 | 0 | Stage IA |
| TCGA-44-A4SU | 1 | 409 | 1 | 260 | 67 | FEMALE | Yes | T1a | N0 | NA | Stage IA |
| TCGA-49-4486 | 1 | 2318 | 1 | 2045 | 72 | MALE | Yes | T1 | N0 | 0 | Stage IA |
| TCGA-49-4487 | 1 | 855 | 1 | 697 | 72 | FEMALE | Yes | T1 | N0 | 0 | Stage IA |
| TCGA-49-4488 | 1 | 869 | 1 | 634 | 74 | MALE | Yes | T2a | N2 | NA | Stage IIIA |
| TCGA-49-4490 | 1 | 385 | 0 | 385 | 45 | FEMALE | Yes | T2 | NX | 0 | Stage IB |
| TCGA-49-4494 | 1 | 1081 | NA | NA | 77 | FEMALE | Yes | T2 | N1 | 0 | Stage IIB |
| TCGA-49-4501 | 1 | 1421 | 1 | 545 | 67 | MALE | No | T2 | N0 | 0 | Stage IB |
| TCGA-49-4505 | 1 | 428 | 1 | 417 | 61 | FEMALE | Yes | T1 | N0 | 0 | Stage IA |
| TCGA-49-4506 | 1 | 999 | 1 | 692 | 68 | MALE | Yes | T2 | N0 | 0 | Stage IA |
| TCGA-49-4507 | 1 | 268 | 1 | 158 | 73 | MALE | Yes | T2 | N2 | 0 | Stage IIIA |
| TCGA-49-4510 | 1 | 896 | 1 | 502 | 51 | MALE | Yes | T2a | N1 | 0 | Stage IIA |
| TCGA-49-4512 | 1 | 905 | 0 | 905 | 69 | FEMALE | No | T1 | N2 | NA | Stage IIIA |
| TCGA-49-4514 | 0 | 1700 | 0 | 1700 | 79 | FEMALE | Yes | T1 | N0 | 0 | Stage IA |
| TCGA-49-6742 | 1 | 488 | 1 | 214 | 70 | FEMALE | Yes | T2 | N0 | 0 | Stage IB |
| TCGA-49-6743 | 0 | 1621 | 0 | 1621 | 81 | MALE | Yes | T1 | N0 | NA | Stage IA |
| TCGA-49-6744 | 0 | 1683 | 0 | 1683 | 64 | FEMALE | Yes | T2a | N0 | NA | Stage IB |
| TCGA-49-6745 | 0 | 522 | 0 | 522 | 82 | FEMALE | Yes | T1 | N0 | 0 | Stage IA |
| TCGA-49-6761 | 0 | 354 | 0 | 354 | 68 | FEMALE | Yes | T1 | N2 | NA | Stage IIIA |
| TCGA-49-6767 | 0 | 677 | NA | NA | 46 | FEMALE | Yes | T3 | N0 | NA | Stage IIB |
| TCGA-49-AAQV | 1 | 677 | NA | NA | 63 | FEMALE | No | T1 | N1 | NA | Stage II |
| TCGA-49-AAR0 | 0 | 4765 | 0 | 4765 | 57 | MALE | Yes | T1 | N0 | NA | Stage IA |
| TCGA-49-AAR2 | 0 | 2224 | 0 | 2224 | 64 | MALE | Yes | T2 | N0 | NA | Stage IB |
| TCGA-49-AAR3 | 0 | 1893 | 0 | 1893 | 69 | MALE |  | T2 | N1 | NA | Stage IIB |
| TCGA-49-AAR4 | 1 | 879 | 0 | 879 | 51 | MALE | Yes | T2 | N2 | NA | Stage IIIA |
| TCGA-49-AAR9 | 1 | 260 | 0 | 260 | 61 | MALE | Yes | T3 | N0 | NA | Stage IIB |
| TCGA-49-AARE | 1 | 1229 | NA | NA | 51 | FEMALE | Yes | T1 | N0 | NA | Stage IA |
| TCGA-49-AARN | 1 | 1135 | NA | NA | 56 | FEMALE | Yes | T1 | N0 | NA | Stage IA |
| TCGA-49-AARO | 0 | 3759 | 1 | 3521 | 39 | FEMALE | Yes | T1a | N0 | NA | Stage IA |
| TCGA-49-AARQ | 0 | 6732 | 0 | 6732 | 41 | FEMALE | Yes | T2 | N0 | NA | Stage I |
| TCGA-49-AARR | 0 | 4992 | 1 | 4812 | 68 | MALE | Yes | T1 | N0 | NA | Stage IA |
| TCGA-4B-A93V | 1 | 300 | 1 | 251 | 52 | FEMALE | Yes | T1b | N0 | 0 | Stage IA |
| TCGA-50-5044 | 1 | 624 | 1 | 483 | 72 | FEMALE |  | T4 | N1 | 0 | Stage IIIB |
| TCGA-50-5045 | 1 | 2174 | 1 | 1433 | 57 | FEMALE |  | T2 | N1 | 0 | [Discrepancy] |
| TCGA-50-5049 | 0 | 3094 | 1 | 2544 | 70 | MALE |  | T2 | N0 | 0 | Stage IA |
| TCGA-50-5051 | 1 | 478 | 1 | 184 | 42 | FEMALE | Yes | T2 | N2 | 0 | Stage IIIA |
| TCGA-50-5055 | 1 | 1830 | 1 | 771 | 79 | FEMALE |  | T1 | N1 | 0 | Stage IIA |
| TCGA-50-5066 | 0 | 1442 | 0 | 1442 | 72 | FEMALE | No | T2 | N0 | 0 | Stage IB |
| TCGA-50-5068 | 1 | 1499 | 1 | 686 | 59 | FEMALE |  | T2 | N1 | NA | Stage IIB |
| TCGA-50-5072 | 1 | 250 | 1 | 213 | 74 | MALE | Yes | T2 | N2 | 0 | Stage IIIA |
| TCGA-50-5930 | 1 | 282 | 1 | 177 | 47 | FEMALE |  | T1b | NX | 0 | Stage IA |
| TCGA-50-5931 | 1 | 434 | 1 | 425 | 75 | MALE | Yes | T3 | N2 | 0 | Stage IIIA |
| TCGA-50-5932 | 1 | 1235 | 1 | 1090 | 75 | MALE |  | T4 | N2 | 0 | Stage IIIB |
| TCGA-50-5933 | 1 | 2393 | 0 | 2393 | 72 | MALE |  | T2 | N2 | 0 | Stage IIIA |
| TCGA-50-5935 | 1 | 653 | 0 | 653 | 86 | FEMALE |  | T1 | N2 | 0 | Stage IIIA |
| TCGA-50-5936 | 1 | 257 | 1 | 150 | 58 | MALE |  | T3 | N1 | 0 | Stage IIIA |
| TCGA-50-5939 | 1 | 460 | 0 | 460 | 85 | FEMALE | Yes | T1b | N1 | 0 | Stage IIA |
| TCGA-50-5941 | 0 | 1474 | 0 | 1474 | 55 | FEMALE | Yes | T2a | N2 | 0 | Stage IIIA |
| TCGA-50-5942 | 0 | 1847 | 1 | 1378 | 67 | FEMALE | Yes | T1 | N0 | 0 | Stage IA |
| TCGA-50-5944 | 0 | 1750 | 0 | 1750 | 69 | FEMALE |  | T1 | N0 | 0 | Stage IA |
| TCGA-50-5946 | 0 | 1617 | 1 | 811 | 62 | MALE | Yes | T2b | N0 | NA | Stage IIA |
| TCGA-50-6590 | 1 | 1288 | 0 | 1288 | 72 | FEMALE | Yes | T2 | N0 | 0 | Stage IB |
| TCGA-50-6591 | 1 | 119 | 0 | 119 | 63 | MALE | No | T3 | N0 | 1 | Stage IIB |
| TCGA-50-6592 | 1 | 777 | 0 | 777 | 71 | FEMALE | Yes | T1 | N0 | 0 | Stage IA |
| TCGA-50-6593 | 1 | 336 | 1 | 266 | 49 | FEMALE | Yes | T2 | N0 | 0 | Stage IB |
| TCGA-50-6594 | 1 | 370 | 1 | 285 | 79 | FEMALE | Yes | T1b | NX | 0 | Stage IA |
| TCGA-50-6595 | 1 | 189 | 1 | 182 | 74 | MALE | Yes | T1 | N0 | 0 | Stage IA |
| TCGA-50-6597 | 1 | 1268 | 0 | 1268 | 79 | FEMALE | No | T2 | N0 | 0 | Stage IB |
| TCGA-50-6673 | 1 | 22 | 0 | 22 | 84 | FEMALE | No | T1 | N0 | 0 | Stage I |
| TCGA-50-7109 | 1 | 308 | 1 | 15 | 60 | FEMALE | Yes | T2 | N1 | 0 | Stage IIB |
| TCGA-50-8457 | 0 | 1125 | 0 | 1125 | 63 | FEMALE | Yes | T1a | N0 | 0 | Stage IA |
| TCGA-50-8459 | 0 | 1119 | 0 | 1119 | 68 | MALE | Yes | T3 | N0 | 0 | Stage IIB |
| TCGA-50-8460 | 0 | 829 | 0 | 829 | 74 | MALE |  | T1a | N0 | 0 | Stage IA |
| TCGA-53-7624 | 1 | 1043 | 1 | 400 | 40 | FEMALE | Yes | T2 | N0 | 1 | Stage IV |
| TCGA-53-7626 | 1 | 929 | 1 | 865 | 76 | FEMALE | Yes | T1 | N1 | 0 | Stage IIA |
| TCGA-53-7813 | 0 | 424 | NA | NA | 51 | FEMALE | Yes | T4 | N0 | 0 | Stage IIIB |
| TCGA-53-A4EZ | 0 | 1071 | 0 | 1071 | 63 | MALE | Yes | T2a | N1 | NA | Stage IIA |
| TCGA-55-1592 | 1 | 701 | 1 | 452 | 65 | MALE | Yes | T2 | N1 | 0 | Stage IIB |
| TCGA-55-1594 | 0 | 1178 | NA | NA | 68 | MALE | Yes | T3 | N1 | 0 | Stage IIIA |
| TCGA-55-1596 | 0 | 2065 | 0 | 2065 | 55 | FEMALE | Yes | T1 | N1 | 0 | Stage IIB |
| TCGA-55-5899 | 0 | 930 | 0 | 930 | 58 | MALE | Yes | T1a | N1 | 0 | [Discrepancy] |
| TCGA-55-6543 | 0 | 435 | 0 | 435 | 60 | FEMALE | Yes | T1b | N0 | NA | Stage IA |
| TCGA-55-6642 | 0 | 2449 | 0 | 2449 | 63 | MALE | Yes | T2 | N0 | NA | Stage IB |
| TCGA-55-6712 | 1 | 171 | 0 | 171 | 71 | MALE | Yes | T2a | N1 | NA | Stage IIA |
| TCGA-55-6968 | 1 | 1293 | 0 | 1293 | 61 | FEMALE | Yes | T1 | N0 | 1 | Stage IA |
| TCGA-55-6970 | 1 | 464 | 1 | 457 | 67 | FEMALE | Yes | T3 | N1 | NA | Stage IIIA |
| TCGA-55-6971 | 0 | 1400 | 0 | 1400 | 59 | MALE | Yes | T2 | N2 | NA | Stage IIIA |
| TCGA-55-6972 | 1 | 1632 | 0 | 1632 | 72 | FEMALE | Yes | T2 | N0 | 0 | Stage IB |
| TCGA-55-6975 | 1 | 118 | 0 | 118 | 61 | MALE | Yes | T2 | N2 | 0 | Stage IIIA |
| TCGA-55-6978 | 1 | 176 | 1 | 42 | 81 | FEMALE | No | T2 | N2 | NA | Stage IIIA |
| TCGA-55-6979 | 1 | 237 | 1 | 195 | 59 | MALE | Yes | T2a | N1 | 0 | Stage IIA |
| TCGA-55-6980 | 0 | 2109 | 0 | 2109 | 56 | MALE | No | T2a | N0 | 0 | Stage IB |
| TCGA-55-6981 | 1 | 1379 | 0 | 1379 | 53 | FEMALE | Yes | T1 | N1 | 0 | Stage IIB |
| TCGA-55-6982 | 1 | 995 | 1 | 183 | 79 | MALE | No | T2 | N0 | 0 | Stage IA |
| TCGA-55-6983 | 0 | 2823 | 0 | 2823 | 81 | MALE | Yes | T1 | N0 | 0 | Stage IA |
| TCGA-55-6984 | 1 | 760 | 1 | 724 | 71 | MALE |  | T2a | N1 | 0 | Stage IIA |
| TCGA-55-6985 | 0 | 1233 | 0 | 1233 | 58 | MALE | Yes | T2 | N1 | NA | Stage IIB |
| TCGA-55-6986 | 0 | 3261 | 0 | 3261 | 74 | FEMALE | No | T1b | N0 | 0 | Stage IA |
| TCGA-55-6987 | 0 | 2137 | 0 | 2137 | 77 | MALE | Yes | T1 | N0 | 0 | Stage IA |
| TCGA-55-7227 | 1 | 952 | 1 | 255 | 77 | MALE | Yes | T3 | N1 | NA | Stage IIIA |
| TCGA-55-7281 | 0 | 872 | 1 | 339 | 70 | FEMALE | Yes | T1b | N0 | 0 | Stage IA |
| TCGA-55-7283 | 0 | 609 | 0 | 609 | 76 | FEMALE | Yes | T1 | N0 | NA | Stage IA |
| TCGA-55-7284 | 1 | 243 | 1 | 231 | 74 | MALE | Yes | T3 | N0 | NA | Stage IIB |
| TCGA-55-7570 | 0 | 824 | 0 | 824 | 60 | MALE | Yes | T2 | N2 | NA | Stage IIIA |
| TCGA-55-7573 | 0 | 487 | 0 | 487 | 72 | FEMALE | Yes | T1b | N0 | NA | Stage IA |
| TCGA-55-7574 | 1 | 995 | 1 | 478 | 64 | MALE | Yes | T1 | N0 | 0 | Stage IA |
| TCGA-55-7576 | 0 | 670 | 0 | 670 | 54 | MALE | Yes | T2 | N0 | 0 | Stage IB |
| TCGA-55-7724 | 0 | 705 | 0 | 705 | 76 | MALE | Yes | T1b | N0 | NA | Stage IA |
| TCGA-55-7725 | 0 | 442 | 0 | 442 | 68 | FEMALE | Yes | T2 | N2 | NA | Stage IIIA |
| TCGA-55-7726 | 0 | 652 | 0 | 652 | 72 | FEMALE | Yes | T2 | N0 | NA | Stage IB |
| TCGA-55-7727 | 0 | 119 | 0 | 119 | 70 | MALE | Yes | T1a | N2 | NA | Stage IIIA |
| TCGA-55-7728 | 0 | 704 | 0 | 704 | 64 | FEMALE | Yes | T2a | N0 | NA | Stage IB |
| TCGA-55-7815 | 0 | 773 | 1 | 592 | 76 | MALE | No | T2a | N0 | NA | Stage IB |
| TCGA-55-7816 | 1 | 468 | 0 | 468 | 49 | FEMALE | No | TX | NX | NA | Stage IV |
| TCGA-55-7903 | 0 | 567 | 0 | 567 | 64 | MALE | Yes | T3 | N0 | NA | Stage IIB |
| TCGA-55-7907 | 1 | 343 | 1 | 294 | 77 | MALE | Yes | T2a | N1 | NA | Stage IIA |
| TCGA-55-7910 | 0 | 1040 | 1 | 1018 | 50 | MALE | Yes | T2a | N2 | 0 | Stage IIIA |
| TCGA-55-7911 | 0 | 537 | 1 | 515 | 70 | FEMALE | Yes | T1 | NX | NA | Stage IA |
| TCGA-55-7913 | 1 | 561 | 1 | 480 | 61 | FEMALE | Yes | T1b | N0 | NA | Stage IA |
| TCGA-55-7914 | 1 | 187 | 0 | 187 | 71 | FEMALE | Yes | T2 | N0 | NA | Stage IB |
| TCGA-55-7994 | 0 | 603 | 0 | 603 | 81 | MALE | Yes | T3 | N0 | NA | Stage IIB |
| TCGA-55-7995 | 0 | 889 | 1 | 468 | 73 | FEMALE | Yes | T1b | N0 | 0 | Stage IA |
| TCGA-55-8085 | 0 | 904 | 0 | 904 | 64 | MALE | Yes | T1b | N0 | 0 | Stage IA |
| TCGA-55-8087 | 0 | 462 | 0 | 462 | 59 | FEMALE | No | T2a | N0 | NA | Stage IB |
| TCGA-55-8089 | 1 | 702 | 0 | 702 | 56 | MALE | Yes | T1a | N0 | 0 | Stage IA |
| TCGA-55-8090 | 1 | 598 | 1 | 548 | 80 | MALE | Yes | T1a | N0 | 0 | Stage IA |
| TCGA-55-8091 | 0 | 600 | 0 | 600 | 74 | MALE | Yes | T2 | N0 | NA | Stage IB |
| TCGA-55-8092 | 1 | 154 | 1 | 127 | 75 | MALE | Yes | T3 | N0 | NA | Stage IIB |
| TCGA-55-8094 | 0 | 541 | 0 | 541 | 51 | MALE | Yes | T2b | N0 | 1 | Stage IV |
| TCGA-55-8096 | 1 | 719 | 1 | 566 | 67 | FEMALE | Yes | T2a | N0 | NA | Stage IB |
| TCGA-55-8097 | 0 | 476 | 0 | 476 | 60 | FEMALE | Yes | T1a | N0 | NA | Stage IA |
| TCGA-55-8203 | 0 | 547 | 0 | 547 | 69 | FEMALE | Yes | T1b | N0 | 0 | Stage IA |
| TCGA-55-8204 | 0 | 515 | 0 | 515 | 87 | FEMALE | Yes | T2a | N0 | NA | Stage IB |
| TCGA-55-8205 | 0 | 599 | 1 | 495 | 76 | FEMALE | Yes | T2b | N0 | 0 | Stage IIA |
| TCGA-55-8206 | 0 | 888 | 0 | 888 | 56 | MALE | No | T1b | N0 | 0 | Stage IA |
| TCGA-55-8207 | 0 | 977 | 0 | 977 | 73 | MALE | Yes | T2a | N0 | NA | Stage IB |
| TCGA-55-8208 | 0 | 674 | 1 | 511 | 73 | FEMALE | Yes | T1b | N0 | 0 | Stage IA |
| TCGA-55-8299 | 1 | 469 | 1 | 274 | 61 | FEMALE | Yes | T1b | N0 | NA | Stage IA |
| TCGA-55-8301 | 0 | 534 | 1 | 238 | 58 | MALE | Yes | T2a | N0 | NA | Stage IB |
| TCGA-55-8302 | 0 | 478 | 0 | 478 | 54 | MALE | Yes | T2 | N0 | NA | Stage IB |
| TCGA-55-8505 | 0 | 440 | 0 | 440 | 62 | MALE | No | T1a | N2 | NA | Stage IIIA |
| TCGA-55-8506 | 0 | 11 | 0 | 11 | 62 | FEMALE | Yes | T3 | N0 | NA | Stage IIB |
| TCGA-55-8507 | 0 | 418 | 0 | 418 | 53 | MALE | Yes | T1a | N0 | NA | Stage IA |
| TCGA-55-8508 | 0 | 617 | 0 | 617 | 60 | FEMALE | Yes | T2a | N1 | NA | Stage IIA |
| TCGA-55-8510 | 0 | 539 | 0 | 539 | 55 | FEMALE | Yes | T2a | N0 | NA | Stage IB |
| TCGA-55-8511 | 0 | 552 | 1 | 460 | 73 | FEMALE | Yes | T2a | N0 | NA | Stage IB |
| TCGA-55-8512 | 1 | 607 | 0 | 607 | 41 | MALE | Yes | T1a | N1 | 1 | Stage IV |
| TCGA-55-8513 | 0 | 791 | 1 | 317 | 77 | FEMALE | No | T3 | N0 | NA | Stage IIB |
| TCGA-55-8514 | 0 | 520 | 0 | 520 | 70 | FEMALE | Yes | T2a | N0 | NA | Stage IB |
| TCGA-55-8614 | 0 | 536 | 0 | 536 | 76 | MALE | Yes | T2a | N0 | NA | Stage IB |
| TCGA-55-8615 | 0 | 446 | 1 | 224 | 67 | MALE | Yes | T3 | N2 | NA | Stage IIIA |
| TCGA-55-8616 | 0 | 48 | 0 | 48 | 58 | FEMALE | Yes | T2a | N0 | 0 | Stage IB |
| TCGA-55-8619 | 0 | 416 | 0 | 416 | 72 | FEMALE | No | T3 | N0 | NA | Stage IIB |
| TCGA-55-8620 | 1 | 375 | 0 | 375 | 60 | MALE | Yes | T1a | N1 | 1 | Stage IV |
| TCGA-55-8621 | 0 | 515 | 0 | 515 | 75 | FEMALE | Yes | T1a | N0 | NA | Stage IA |
| TCGA-55-A48X | 0 | 689 | 1 | 615 | 63 | FEMALE | Yes | T1b | N1 | 0 | Stage IIA |
| TCGA-55-A48Y | 0 | 630 | 0 | 630 | 69 | MALE | Yes | T2b | N0 | 0 | Stage IIA |
| TCGA-55-A48Z | 0 | 651 | 1 | 536 | 60 | FEMALE | Yes | T1a | N3 | NA | Stage IIIB |
| TCGA-55-A490 | 1 | 99 | 0 | 99 | 78 | MALE | Yes | T2b | N0 | NA | Stage IIA |
| TCGA-55-A491 | 0 | 626 | 0 | 626 | 81 | FEMALE | Yes | T1b | N0 | NA | Stage IA |
| TCGA-55-A492 | 0 | 596 | 0 | 596 | 70 | FEMALE | Yes | T1a | N0 | NA | Stage IA |
| TCGA-55-A493 | 0 | 28 | 0 | 28 | 54 | FEMALE | Yes | T2a | N0 | 0 | Stage IB |
| TCGA-55-A494 | 0 | 481 | 0 | 481 | 61 | FEMALE | Yes | T2a | N0 | NA | Stage IB |
| TCGA-55-A4DF | 1 | 614 | 1 | 515 | 88 | MALE | Yes | T1b | N0 | NA | Stage IA |
| TCGA-55-A4DG | 0 | 608 | 0 | 608 | 71 | MALE | Yes | T1b | N0 | NA | Stage IA |
| TCGA-55-A57B | 0 | 546 | 0 | 546 | 80 | FEMALE | No | T1b | N0 | 0 | Stage IA |
| TCGA-62-8394 | 1 | 139 | 0 | 139 | 65 | FEMALE | No | T4 | N2 | 0 | Stage IIIB |
| TCGA-62-8395 | 0 | 1216 | 1 | 395 | 80 | FEMALE | No | T3 | N0 | 0 | Stage IIB |
| TCGA-62-8397 | 0 | 1289 | 0 | 1289 | 70 | FEMALE | No | T3 | N0 | 0 | Stage IIB |
| TCGA-62-8398 | 1 | 444 | 0 | 444 | 55 | MALE | Yes | T2 | N2 | 0 | Stage IIIA |
| TCGA-62-8399 | 0 | 2696 | 0 | 2696 | 62 | MALE | Yes | T2 | N2 | 0 | Stage IIIA |
| TCGA-62-8402 | 1 | 1498 | 1 | 772 | 73 | FEMALE | No | T2 | N2 | 0 | Stage IIIA |
| TCGA-62-A46O | 1 | 1454 | NA | NA | 65 | FEMALE | Yes | T2 | N0 | 0 | Stage IB |
| TCGA-62-A46P | 1 | 594 | NA | NA | 65 | MALE | Yes | T2 | N0 | 0 | Stage IB |
| TCGA-62-A46R | 1 | 1725 | 0 | 1725 | 54 | FEMALE | Yes | T2 | N0 | 0 | Stage IB |
| TCGA-62-A46S | 1 | 1653 | NA | NA | 73 | MALE | Yes | T2 | N0 | 0 | Stage IB |
| TCGA-62-A46V | 0 | 2199 | 0 | 2199 | 78 | FEMALE | Yes | T2 | N0 | 0 | Stage IB |
| TCGA-62-A46Y | 1 | 414 | NA | NA | 70 | FEMALE | No | T2 | N2 | 0 | Stage IIIA |
| TCGA-62-A470 | 1 | 1194 | NA | NA | 84 | MALE | Yes | T2 | N0 | 0 | Stage IB |
| TCGA-62-A471 | 0 | 1246 | 0 | 1246 | 64 | MALE | Yes | T2b | N1 | 0 | Stage IIB |
| TCGA-62-A472 | 0 | 910 | 1 | 290 | 70 | MALE | Yes | T3 | N0 | 0 | Stage IIB |
| TCGA-64-1676 | 0 | 1728 | 0 | 1728 | 58 | MALE | Yes | T1a | N0 | 0 | Stage IA |
| TCGA-64-1677 | 1 | 628 | 1 | 360 | 77 | FEMALE | Yes | T2 | N2 | 0 | Stage IIIA |
| TCGA-64-1678 | 0 | 1189 | 0 | 1189 | 70 | FEMALE | Yes | T2b | N0 | 0 | [Discrepancy] |
| TCGA-64-1679 | 0 | 2488 | 0 | 2488 | 58 | FEMALE | Yes | T1 | N2 | 0 | Stage IIIA |
| TCGA-64-1680 | 0 | 1126 | 0 | 1126 | 63 | MALE | Yes | T2a | N2 | 1 | Stage IV |
| TCGA-64-1681 | 1 | 1167 | 1 | 439 | 61 | FEMALE | Yes | T1 | N0 | 0 | Stage IA |
| TCGA-64-5774 | 0 | 2676 | 1 | 277 | 60 | MALE | Yes | T2 | N0 | 0 | Stage IB |
| TCGA-64-5775 | 1 | 62 | 0 | 62 | 71 | MALE | Yes | T4 | N0 | 0 | Stage IIIA |
| TCGA-64-5778 | 0 | 1305 | 1 | 1168 | 60 | MALE | Yes | T2 | N0 | 0 | Stage IB |
| TCGA-64-5779 | 0 | 864 | 1 | 795 | 61 | MALE | Yes | T2 | N2 | 0 | Stage IIIA |
| TCGA-64-5781 | 0 | 1559 | 1 | 96 | 55 | FEMALE | Yes | T2 | N0 | 0 | Stage IB |
| TCGA-64-5815 | 0 | 866 | NA | NA | 74 | MALE | Yes | T2 | N1 | 0 | Stage IIB |
| TCGA-67-3770 | 0 | 610 | NA | NA | 70 | FEMALE | Yes | T1 | N0 | 0 | Stage IA |
| TCGA-67-3771 | 0 | 610 | NA | NA | 77 | FEMALE | Yes | T1 | N0 | 0 | Stage IA |
| TCGA-67-3772 | 0 | 573 | NA | NA | 82 | FEMALE | No | T2 | N0 | 0 | Stage IB |
| TCGA-67-3773 | 0 | 427 | NA | NA | 84 | FEMALE | Yes | T2 | N0 | 0 | Stage IB |
| TCGA-67-3774 | 0 | 385 | NA | NA | 73 | FEMALE | Yes | T2 | N0 | 0 | Stage IB |
| TCGA-67-4679 | 0 | 448 | NA | NA | 69 | MALE | Yes | T3 | N0 | 0 | [Discrepancy] |
| TCGA-67-6215 | 0 | 174 | 0 | 174 | 52 | FEMALE | No | T2a | N0 | 0 | Stage IB |
| TCGA-67-6216 | 0 | 141 | 0 | 141 | 57 | FEMALE | No | T1a | N0 | 0 | Stage IA |
| TCGA-67-6217 | 0 | 422 | 1 | 296 | 73 | FEMALE | Yes | T2a | N1 | 0 | Stage IIA |
| TCGA-69-7760 | 0 | 202 | 0 | 202 | 73 | MALE | No | T3 | N0 | 0 | Stage IIB |
| TCGA-69-7761 | 0 | 186 | 0 | 186 | 84 | MALE | Yes | T2a | N0 | NA | Stage IB |
| TCGA-69-7763 | 0 | 690 | 0 | 690 | 69 | MALE | Yes | T1b | N0 | 0 | Stage IA |
| TCGA-69-7764 | 0 | 414 | 0 | 414 | 75 | MALE | Yes | T1b | N0 | 0 | Stage IA |
| TCGA-69-7765 | 0 | 165 | 0 | 165 | 56 | MALE | Yes | T4 | N0 | NA | [Discrepancy] |
| TCGA-69-7973 | 0 | 230 | 0 | 230 | 42 | FEMALE | Yes | T2a | N0 | 0 | Stage IB |
| TCGA-69-7974 | 0 | 184 | 0 | 184 | 54 | FEMALE | Yes | T2a | N2 | NA | Stage IIIA |
| TCGA-69-7978 | 0 | 134 | 0 | 134 | 59 | MALE | Yes | T2b | N1 | NA | Stage IIB |
| TCGA-69-7979 | 0 | 408 | NA | NA | 71 | FEMALE | Yes | T2a | N0 | NA | Stage IB |
| TCGA-69-7980 | 0 | 362 | 0 | 362 | 70 | FEMALE | Yes | T1b | N0 | 0 | Stage I |
| TCGA-69-8253 | 0 | 426 | 0 | 426 | 59 | FEMALE | Yes | T1a | N1 | NA | Stage IIA |
| TCGA-69-8254 | 0 | 409 | 0 | 409 | 85 | MALE | Yes | T2b | NA | NA | NA |
| TCGA-69-8255 | 0 | 129 | 0 | 129 | 71 | MALE | Yes | T1a | 1 | 0 | 1 |
| TCGA-69-8453 | 0 | 813 | 0 | 813 | 77 | MALE | Yes | T3 | 1 | NA | 2 |
| TCGA-69-A59K | 0 | 522 | 0 | 522 | 60 | FEMALE | Yes | T3 | 1 | 0 | 2 |
| TCGA-71-6725 | 0 | 256 | 1 | 164 | 48 | FEMALE | Yes | T2 | 1 | 0 | 1 |
| TCGA-71-8520 | 1 | 210 | 1 | 179 | 60 | FEMALE | No | T2 | 1 | 0 | 1 |
| TCGA-73-4658 | 1 | 1600 | 0 | 1600 | 80 | FEMALE | Yes | T1 | 1 | 0 | 1 |
| TCGA-73-4659 | 1 | 711 | 1 | 316 | 66 | FEMALE | Yes | T2 | 3 | 0 | 3 |
| TCGA-73-4662 | 0 | 2515 | 0 | 2515 | 65 | FEMALE | Yes | T2 | 1 | 0 | 1 |
| TCGA-73-4666 | 0 | 800 | NA | NA | 52 | MALE | Yes | T2 | 1 | 1 | 4 |
| TCGA-73-4668 | 0 | 467 | 1 | 282 | 66 | FEMALE | Yes | T2 | 2 | 0 | 2 |
| TCGA-73-4670 | 0 | 131 | 0 | 131 | 69 | FEMALE | Yes | T1 | 1 | 1 | 4 |
| TCGA-73-4675 | 1 | 922 | 1 | 361 | 59 | FEMALE | Yes | T2 | 2 | 0 | 3 |
| TCGA-73-4676 | 1 | 281 | NA | NA | 45 | MALE | Yes | T2 | 2 | 0 | 2 |
| TCGA-73-4677 | 1 | 38 | 0 | 38 | 74 | FEMALE | Yes | T2 | 1 | 0 | 1 |
| TCGA-73-7498 | 0 | 1189 | 0 | 1189 | 58 | FEMALE | Yes | T1b | 1 | 0 | 1 |
| TCGA-73-7499 | 1 | 1531 | 1 | 1447 | 81 | FEMALE | No | T2a | 1 | 0 | 1 |
| TCGA-73-A9RS | 1 | 340 | 0 | 340 | 41 | MALE | Yes | T3 | 1 | 0 | 2 |
| TCGA-75-5122 | 1 | NA | NA | NA | NA | MALE | Yes | T2 | 1 | 0 | 1 |
| TCGA-75-5125 | 1 | 2027 | 1 | 1752 | NA | MALE | Yes | T2 | 2 | 0 | 2 |
| TCGA-75-5126 | NA | NA | NA | NA | NA | FEMALE | Yes | T3 | 3 | 0 | 3 |
| TCGA-75-5146 | 0 | 2368 | 1 | 1773 | NA | MALE | Yes | T2 | 1 | 0 | 1 |
| TCGA-75-5147 | 0 | 1333 | 0 | 1333 | NA | FEMALE | No | T2 | 1 | 0 | 1 |
| TCGA-75-6203 | NA | NA | NA | NA | NA | FEMALE | No | T2 | 3 | 0 | 3 |
| TCGA-75-6205 | 1 | NA | NA | NA | NA | FEMALE | No | T2a | 1 | 0 | 1 |
| TCGA-75-6206 | 0 | 2590 | 0 | 2590 | NA | MALE | Yes | T2 | 1 | 0 | 1 |
| TCGA-75-6207 | 1 | NA | NA | NA | NA | MALE | Yes | T2 | 3 | 0 | 3 |
| TCGA-75-6211 | 1 | NA | NA | NA | NA | FEMALE | Yes | T2 | 1 | 0 | 1 |
| TCGA-75-6212 | 1 | 1516 | 1 | 1340 | NA | FEMALE | No | T2 | 2 | 0 | 2 |
| TCGA-75-6214 | 1 | 1115 | 1 | 419 | NA | FEMALE | Yes | T2 | 3 | 0 | 3 |
| TCGA-75-7025 | 0 | 3305 | 1 | 1481 | NA | MALE | Yes | T2 | 1 | 0 | 1 |
| TCGA-75-7027 | 0 | 3059 | 1 | 2949 | NA | MALE | Yes | T2 | 1 | 0 | 1 |
| TCGA-75-7030 | NA | NA | NA | NA | NA | MALE | No | T3 | 1 | 0 | 2 |
| TCGA-75-7031 | 0 | NA | NA | NA | NA | FEMALE | Yes | T2 | 1 | 0 | 1 |
| TCGA-78-7143 | 1 | 4961 | 1 | 4608 | 62 | FEMALE | No | T2 | 1 | 0 | 1 |
| TCGA-78-7145 | 1 | 826 | 1 | 422 | 52 | FEMALE | Yes | T4 | 2 | 1 | 4 |
| TCGA-78-7146 | 1 | 173 | NA | NA | 71 | FEMALE | Yes | T2 | 3 | 0 | 3 |
| TCGA-78-7147 | 1 | 586 | 1 | 578 | 67 | FEMALE | Yes | T2 | 2 | 0 | 2 |
| TCGA-78-7148 | 1 | 626 | 1 | 182 | 71 | MALE | Yes | T2 | 2 | 0 | 2 |
| TCGA-78-7149 | 0 | 3940 | NA | NA | 71 | MALE | Yes | T4 | 1 | 0 | 3 |
| TCGA-78-7150 | 1 | 666 | 1 | 139 | 59 | MALE | Yes | T2 | 2 | 0 | 2 |
| TCGA-78-7152 | 1 | 1215 | 1 | 1202 | 65 | MALE | Yes | T2 | 1 | 0 | 1 |
| TCGA-78-7153 | 0 | 3635 | NA | NA | 65 | FEMALE | Yes | T2 | 1 | 0 | 1 |
| TCGA-78-7154 | 1 | 593 | NA | NA | 72 | MALE | Yes | T3 | 3 | 0 | 3 |
| TCGA-78-7155 | 1 | 1171 | 1 | 209 | 68 | MALE | Yes | T2 | 1 | 0 | 1 |
| TCGA-78-7156 | 1 | 976 | NA | NA | 62 | MALE | Yes | T4 | 2 | 1 | 4 |
| TCGA-78-7158 | 1 | 179 | 1 | 132 | 59 | FEMALE | Yes | T4 | 3 | 0 | 3 |
| TCGA-78-7159 | 0 | 1974 | NA | NA | 60 | FEMALE | Yes | T1 | NA | 0 | 1 |
| TCGA-78-7160 | 1 | 697 | NA | NA | 61 | MALE | Yes | T4 | 3 | 1 | 4 |
| TCGA-78-7161 | 1 | 291 | 1 | 162 | 69 | FEMALE | Yes | T3 | 1 | 0 | 2 |
| TCGA-78-7162 | 1 | 3169 | 1 | 2740 | 75 | MALE | Yes | T1 | 1 | 0 | 1 |
| TCGA-78-7163 | 0 | 7248 | NA | NA | 60 | MALE | Yes | T2 | 1 | 0 | 1 |
| TCGA-78-7166 | 1 | 258 | 0 | 258 | 84 | MALE | Yes | T2 | 2 | 0 | 2 |
| TCGA-78-7167 | 1 | 2681 | 1 | 726 | 77 | MALE | Yes | T2 | 1 | 1 | 4 |
| TCGA-78-7220 | 1 | 807 | 1 | 531 | 53 | FEMALE | Yes | T2 | 3 | 0 | 3 |
| TCGA-78-7535 | 1 | 949 | 1 | 809 | 45 | MALE | Yes | T2 | 1 | 0 | 1 |
| TCGA-78-7536 | 1 | 244 | 1 | 231 | 69 | MALE | Yes | T2 | 3 | 0 | 3 |
| TCGA-78-7537 | 1 | 1622 | NA | NA | 72 | MALE | Yes | T2 | 1 | 0 | 1 |
| TCGA-78-7539 | 0 | 791 | 1 | 688 | 75 | FEMALE | Yes | T2b | 1 | 0 | 2 |
| TCGA-78-7540 | 1 | 1197 | NA | NA | 66 | FEMALE | No | T1 | 1 | 0 | 1 |
| TCGA-78-7542 | 1 | 321 | NA | NA | 56 | MALE | Yes | T2 | 1 | 0 | 1 |
| TCGA-78-7633 | 1 | 1528 | 1 | 1450 | 67 | MALE | Yes | T2 | 1 | 0 | 1 |
| TCGA-78-8640 | 0 | 7062 | 0 | 7062 | 59 | MALE | Yes | T1 | 2 | 0 | 2 |
| TCGA-78-8648 | 1 | 1209 | NA | NA | 58 | FEMALE | Yes | T3 | 1 | 0 | 2 |
| TCGA-78-8655 | 0 | 2360 | NA | NA | 77 | FEMALE | Yes | T1 | 1 | 0 | 1 |
| TCGA-78-8660 | 1 | 321 | NA | NA | 69 | MALE | Yes | T2 | 2 | 0 | 2 |
| TCGA-78-8662 | 1 | 3361 | NA | NA | 53 | FEMALE | Yes | T2 | 1 | 0 | 1 |
| TCGA-80-5607 | NA | NA | NA | NA | NA | FEMALE | Yes | T2 | 2 | 0 | 2 |
| TCGA-80-5608 | 0 | 2832 | 0 | 2832 | NA | FEMALE | Yes | T1 | 1 | 0 | 1 |
| TCGA-80-5611 | 0 | 2595 | 0 | 2595 | NA | MALE | Yes | T2 | 1 | 0 | 1 |
| TCGA-83-5908 | 0 | 824 | 0 | 824 | 59 | FEMALE | Yes | T1 | 1 | 0 | 1 |
| TCGA-86-6562 | 1 | 376 | 1 | 274 | 52 | MALE | No | T2a | 2 | 0 | 2 |
| TCGA-86-6851 | 0 | 179 | 0 | 179 | 73 | FEMALE | Yes | T1b | 2 | 0 | 2 |
| TCGA-86-7701 | 0 | 947 | 1 | 900 | 66 | MALE | No | T2 | 1 | 1 | 4 |
| TCGA-86-7711 | 1 | 1046 | 0 | 1046 | 70 | FEMALE | Yes | T1 | 2 | 0 | 2 |
| TCGA-86-7713 | 0 | 1157 | 0 | 1157 | 70 | MALE | No | T2a | 1 | 0 | 2 |
| TCGA-86-7714 | 1 | 625 | 0 | 625 | 61 | FEMALE | Yes | T1b | 3 | 0 | 3 |
| TCGA-86-7953 | 0 | 997 | 0 | 997 | 69 | FEMALE | No | T1b | 1 | 0 | 1 |
| TCGA-86-7954 | 0 | 605 | 0 | 605 | 68 | FEMALE | Yes | T2 | 1 | 0 | 1 |
| TCGA-86-7955 | 0 | 1072 | 1 | 820 | 62 | MALE | No | T2a | 1 | 0 | 1 |
| TCGA-86-8054 | 0 | 1148 | 0 | 1148 | 61 | MALE | Yes | T2b | 2 | 0 | 2 |
| TCGA-86-8055 | 1 | 124 | 0 | 124 | 79 | MALE | Yes | T2a | 2 | 0 | 2 |
| TCGA-86-8056 | 0 | 139 | 0 | 139 | 63 | FEMALE | Yes | T4 | 1 | 0 | 3 |
| TCGA-86-8073 | 0 | 740 | 0 | 740 | 58 | MALE | Yes | T2a | 1 | 0 | 1 |
| TCGA-86-8074 | 0 | 24 | NA | NA | 62 | FEMALE | Yes | T1b | 2 | 0 | 2 |
| TCGA-86-8075 | 1 | 694 | 0 | 694 | 66 | FEMALE | No | T2 | 1 | 0 | 1 |
| TCGA-86-8076 | 0 | 993 | 0 | 993 | 42 | MALE | No | T1 | 1 | 0 | 1 |
| TCGA-86-8278 | 0 | 944 | 0 | 944 | 63 | FEMALE | No | T2 | 2 | 0 | 2 |
| TCGA-86-8279 | 0 | 949 | 0 | 949 | 46 | MALE | No | T2a | 2 | 0 | 2 |
| TCGA-86-8280 | 0 | 701 | 0 | 701 | 54 | FEMALE | No | T2b | 1 | 0 | 2 |
| TCGA-86-8281 | NA | NA | NA | NA | 75 | MALE | Yes | T1 | NA | 0 | 1 |
| TCGA-86-8358 | 0 | 653 | 0 | 653 | 44 | MALE | Yes | T2a | 1 | 0 | 1 |
| TCGA-86-8359 | 1 | 444 | NA | NA | 52 | MALE | Yes | T3 | 3 | 0 | 3 |
| TCGA-86-8585 | 0 | 353 | 0 | 353 | 57 | MALE | No | T2a | 1 | 0 | 1 |
| TCGA-86-8668 | 0 | 423 | 0 | 423 | 61 | FEMALE | No | T1b | 1 | 0 | 1 |
| TCGA-86-8669 | 0 | 938 | 1 | 849 | 64 | MALE | Yes | T1b | 1 | 0 | 1 |
| TCGA-86-8671 | 0 | 839 | 0 | 839 | 72 | FEMALE | No | T2b | 2 | 0 | 2 |
| TCGA-86-8672 | 1 | 19 | 0 | 19 | 59 | MALE | No | T3 | 1 | 0 | 2 |
| TCGA-86-8673 | 0 | 862 | 1 | 636 | 61 | MALE | Yes | T2 | 1 | 0 | 1 |
| TCGA-86-8674 | 0 | 806 | 0 | 806 | 50 | MALE | Yes | T2a | 2 | 0 | 2 |
| TCGA-86-A456 | 0 | 896 | 0 | 896 | 78 | FEMALE | Yes | T1a | 1 | 0 | 1 |
| TCGA-86-A4D0 | 1 | 116 | 0 | 116 | 48 | MALE | Yes | T2b | 1 | 0 | 2 |
| TCGA-86-A4JF | 1 | 737 | 0 | 737 | 56 | MALE | Yes | T3 | 1 | 0 | 2 |
| TCGA-86-A4P7 | 0 | 415 | 0 | 415 | 63 | FEMALE | No | T2a | 1 | 0 | 1 |
| TCGA-86-A4P8 | 0 | 805 | 0 | 805 | 59 | FEMALE | No | T1b | 3 | NA | 3 |
| TCGA-91-6828 | 0 | 323 | 0 | 323 | 70 | FEMALE | Yes | T1 | 1 | 0 | 1 |
| TCGA-91-6829 | 1 | 1258 | 0 | 1258 | 78 | MALE | Yes | T2 | 1 | NA | 1 |
| TCGA-91-6830 | 0 | 60 | 1 | 18 | 65 | FEMALE | Yes | T1 | 2 | NA | 2 |
| TCGA-91-6831 | 0 | 310 | 0 | 310 | 66 | FEMALE | Yes | T2 | 1 | NA | 1 |
| TCGA-91-6835 | 0 | 79 | 0 | 79 | 81 | FEMALE | Yes | T2a | 1 | 0 | 1 |
| TCGA-91-6836 | 0 | 417 | 0 | 417 | 52 | FEMALE | Yes | T1a | 1 | NA | 1 |
| TCGA-91-6840 | 0 | 372 | 0 | 372 | 59 | FEMALE | Yes | T1b | 1 | 0 | 1 |
| TCGA-91-6847 | 0 | 842 | 1 | 772 | 62 | MALE | Yes | T3 | 1 | NA | 1 |
| TCGA-91-6848 | 0 | 224 | 0 | 224 | 59 | MALE | Yes | T2 | 3 | NA | 3 |
| TCGA-91-6849 | 0 | 35 | NA | NA | 75 | MALE | Yes | T2a | 3 | NA | 3 |
| TCGA-91-7771 | 0 | 492 | 0 | 492 | 62 | MALE | Yes | T3 | 1 | NA | 2 |
| TCGA-91-8496 | 0 | 505 | 0 | 505 | 63 | FEMALE | No | T2a | NA | NA | 1 |
| TCGA-91-8497 | 1 | 434 | 0 | 434 | 75 | FEMALE | No | T1a | 1 | NA | 1 |
| TCGA-91-8499 | 0 | 36 | 0 | 36 | 76 | FEMALE | Yes | T1b | 1 | NA | 1 |
| TCGA-91-A4BC | 0 | 44 | NA | NA | 59 | MALE | Yes | T2b | 1 | NA | 2 |
| TCGA-91-A4BD | 0 | 603 | 0 | 603 | 78 | MALE | Yes | T1b | 2 | NA | 2 |
| TCGA-93-7347 | 0 | 683 | 0 | 683 | 76 | FEMALE | Yes | T1a | 1 | NA | 1 |
| TCGA-93-7348 | 0 | 531 | 0 | 531 | 75 | MALE | Yes | T4 | 1 | NA | 1 |
| TCGA-93-8067 | 0 | 186 | 0 | 186 | 77 | MALE | Yes | T2a | 1 | NA | 1 |
| TCGA-93-A4JN | 0 | 718 | 0 | 718 | 71 | MALE | Yes | T2a | 1 | 1 | 4 |
| TCGA-93-A4JO | 1 | 33 | 0 | 33 | 70 | MALE | Yes | T1a | 1 | NA | 1 |
| TCGA-93-A4JP | 0 | 578 | 1 | 497 | 64 | MALE | No | TX | NA | 1 | 4 |
| TCGA-93-A4JQ | 0 | 526 | 0 | 526 | 49 | MALE | Yes | T1b | 1 | NA | 1 |
| TCGA-95-7039 | 0 | 1272 | 1 | 1258 | 54 | FEMALE | Yes | T3 | 1 | NA | 2 |
| TCGA-95-7043 | 1 | 503 | NA | NA | 63 | FEMALE | Yes | T1a | 1 | NA | 1 |
| TCGA-95-7562 | 1 | 87 | 0 | 87 | 71 | MALE | Yes | T2a | 2 | 0 | 2 |
| TCGA-95-7567 | 0 | 568 | 0 | 568 | 61 | MALE | Yes | T2b | 2 | 0 | 2 |
| TCGA-95-7944 | 0 | 377 | 0 | 377 | 71 | MALE | Yes | T1a | 1 | 0 | 1 |
| TCGA-95-7947 | 0 | 477 | 0 | 477 | 67 | MALE | Yes | T1a | 1 | 0 | 1 |
| TCGA-95-7948 | 0 | 476 | 0 | 476 | 42 | FEMALE | Yes | T2a | 1 | 0 | 1 |
| TCGA-95-8039 | 0 | 830 | 1 | 228 | 72 | MALE | No | T1 | 1 | NA | 1 |
| TCGA-95-8494 | 0 | 84 | 0 | 84 | 67 | MALE | Yes | T2a | 2 | 0 | 2 |
| TCGA-95-A4VK | 0 | 651 | 1 | 496 | 74 | FEMALE | Yes | T2b | 3 | 0 | 3 |
| TCGA-95-A4VN | 0 | 553 | 0 | 553 | 62 | FEMALE | Yes | T2a | 2 | 0 | 2 |
| TCGA-95-A4VP | 0 | 605 | 1 | 216 | 66 | FEMALE | Yes | T2b | 3 | 0 | 3 |
| TCGA-97-7546 | 0 | 1285 | 1 | 1255 | 76 | FEMALE | Yes | T1 | 1 | NA | 1 |
| TCGA-97-7547 | 0 | 1965 | 1 | 1147 | 67 | FEMALE | Yes | T2 | 1 | NA | 1 |
| TCGA-97-7552 | 0 | 1932 | 1 | 798 | 70 | MALE | Yes | T2 | 1 | NA | 1 |
| TCGA-97-7553 | 0 | 1870 | 0 | 1870 | 58 | FEMALE | Yes | T1 | 1 | NA | 1 |
| TCGA-97-7554 | 0 | 775 | 0 | 775 | 83 | FEMALE | Yes | T2a | 3 | 0 | 3 |
| TCGA-97-7937 | 0 | 564 | 0 | 564 | 65 | MALE | Yes | T2a | 1 | NA | 1 |
| TCGA-97-7938 | 1 | 18 | 0 | 18 | 76 | FEMALE | Yes | T1a | 1 | NA | 1 |
| TCGA-97-7941 | 0 | 484 | 0 | 484 | 72 | FEMALE | Yes | T1b | 1 | NA | 1 |
| TCGA-97-8171 | 0 | 568 | 1 | 441 | 81 | MALE | Yes | T2a | 3 | 1 | 4 |
| TCGA-97-8172 | 0 | 545 | 0 | 545 | 75 | FEMALE | Yes | T2a | 1 | 0 | 1 |
| TCGA-97-8174 | 1 | 164 | 0 | 164 | 67 | MALE | Yes | T2b | 1 | 0 | 2 |
| TCGA-97-8175 | 0 | 551 | 1 | 294 | 55 | FEMALE | Yes | T2a | 1 | 0 | 1 |
| TCGA-97-8176 | 1 | 468 | 0 | 468 | 63 | MALE | Yes | T3 | 2 | 0 | 3 |
| TCGA-97-8177 | 0 | 499 | 0 | 499 | 59 | FEMALE | No | T2a | 1 | 0 | 1 |
| TCGA-97-8179 | 0 | 435 | 0 | 435 | 72 | MALE | Yes | T1a | 1 | 0 | 1 |
| TCGA-97-8547 | 0 | 657 | 0 | 657 | 78 | FEMALE | No | T2a | 3 | NA | 3 |
| TCGA-97-8552 | 0 | 626 | 0 | 626 | 55 | FEMALE | No | T1a | 1 | NA | 1 |
| TCGA-97-A4LX | 0 | 614 | 0 | 614 | 81 | MALE | Yes | T2a | 1 | 0 | 1 |
| TCGA-97-A4M0 | 0 | 652 | 0 | 652 | 60 | FEMALE | Yes | T2a | 1 | 0 | 1 |
| TCGA-97-A4M1 | 0 | 601 | 0 | 601 | 52 | FEMALE | Yes | T1a | 1 | 0 | 1 |
| TCGA-97-A4M2 | 0 | 624 | 0 | 624 | 66 | MALE | Yes | T1a | 1 | 0 | 1 |
| TCGA-97-A4M3 | 0 | 540 | 1 | 54 | 69 | FEMALE | Yes | T1b | 1 | 0 | 1 |
| TCGA-97-A4M5 | 0 | 634 | 0 | 634 | 83 | MALE | Yes | T1b | 1 | 0 | 1 |
| TCGA-97-A4M6 | 0 | 568 | 0 | 568 | 45 | FEMALE | No | T1a | 1 | 0 | 1 |
| TCGA-97-A4M7 | 0 | 629 | 0 | 629 | 74 | MALE | Yes | T1b | 1 | 0 | 1 |
| TCGA-99-7458 | 0 | 747 | 0 | 747 | 74 | FEMALE | Yes | T4 | 1 | 0 | 3 |
| TCGA-99-8025 | 0 | 1060 | 0 | 1060 | 72 | FEMALE | Yes | T3 | 3 | 0 | 3 |
| TCGA-99-8028 | 0 | 1118 | 0 | 1118 | 50 | FEMALE | Yes | T1a | 1 | 0 | 1 |
| TCGA-99-8032 | 0 | 44 | NA | NA | 61 | MALE | Yes | T1a | 1 | 0 | 1 |
| TCGA-99-8033 | 1 | 656 | 0 | 656 | 74 | FEMALE | No | TX | NA | 1 | 4 |
| TCGA-99-AA5R | 0 | 658 | 0 | 658 | 70 | FEMALE | Yes | T1a | 1 | 0 | 1 |
| TCGA-J2-8192 | 0 | 739 | 1 | 482 | 65 | FEMALE | No | T2a | 2 | NA | 2 |
| TCGA-J2-8194 | 0 | 724 | 1 | 470 | 69 | FEMALE | Yes | T3 | 1 | NA | 2 |
| TCGA-J2-A4AD | 1 | 550 | 1 | 524 | 61 | FEMALE | Yes | T1a | 1 | NA | 1 |
| TCGA-J2-A4AE | 0 | 1079 | 0 | 1079 | 77 | FEMALE | No | T1a | 1 | NA | 1 |
| TCGA-J2-A4AG | 0 | 988 | 0 | 988 | 66 | FEMALE | Yes | T1b | 1 | NA | 1 |
| TCGA-L4-A4E5 | 0 | 578 | 0 | 578 | 48 | FEMALE | Yes | T1 | 1 | 0 | 1 |
| TCGA-L4-A4E6 | 0 | 435 | 0 | 435 | 67 | MALE | Yes | T1 | 1 | 0 | 1 |
| TCGA-L9-A443 | 1 | 193 | 0 | 193 | 63 | FEMALE | Yes | T1a | 1 | NA | 1 |
| TCGA-L9-A444 | 0 | 307 | 0 | 307 | 60 | FEMALE | Yes | T1a | 1 | NA | 1 |
| TCGA-L9-A50W | 1 | 442 | 1 | 389 | 75 | MALE | Yes | T1b | 2 | NA | 2 |
| TCGA-L9-A5IP | 1 | 58 | NA | NA | 40 | FEMALE | Yes | T3 | 3 | 1 | 4 |
| TCGA-L9-A743 | 0 | 664 | 0 | 664 | 56 | MALE | Yes | T2a | 2 | 0 | 2 |
| TCGA-L9-A7SV | 0 | 565 | 0 | 565 | 69 | MALE | Yes | T2a | 2 | 0 | 2 |
| TCGA-L9-A8F4 | 0 | 476 | 0 | 476 | 64 | FEMALE | Yes | T2a | 1 | NA | 1 |
| TCGA-MN-A4N1 | 0 | 827 | 0 | 827 | 60 | MALE | Yes | T2a | 2 | 0 | 2 |
| TCGA-MN-A4N4 | 0 | 1175 | 0 | 1175 | 57 | MALE | Yes | T1b | 1 | 0 | 1 |
| TCGA-MN-A4N5 | 0 | 84 | 0 | 84 | 63 | MALE | Yes | T1a | 1 | 0 | 1 |
| TCGA-MP-A4SV | 1 | 2620 | NA | NA | 67 | MALE | Yes | T2 | 1 | 0 | 1 |
| TCGA-MP-A4SW | 1 | 1778 | NA | NA | 53 | MALE | Yes | T2 | 2 | 0 | 2 |
| TCGA-MP-A4SY | 1 | 1501 | NA | NA | 61 | MALE | Yes | T2 | 2 | 0 | 2 |
| TCGA-MP-A4T4 | 1 | 2617 | NA | NA | 68 | FEMALE | Yes | T2 | 2 | 0 | 2 |
| TCGA-MP-A4T6 | 1 | 1790 | 0 | 1790 | 76 | FEMALE | Yes | T1 | 3 | NA | 3 |
| TCGA-MP-A4T7 | 1 | 167 | NA | NA | 75 | FEMALE | Yes | T2 | 1 | 1 | 4 |
| TCGA-MP-A4T8 | 1 | 161 | NA | NA | 68 | MALE | Yes | T2 | 3 | 0 | 3 |
| TCGA-MP-A4T9 | 1 | 1265 | 1 | 1248 | 54 | FEMALE | Yes | T2 | 3 | NA | 3 |
| TCGA-MP-A4TA | 1 | 950 | NA | NA | 75 | FEMALE | Yes | T1 | 1 | 0 | 1 |
| TCGA-MP-A4TC | 1 | 74 | 0 | 74 | 77 | MALE | Yes | T1 | 3 | 0 | 3 |
| TCGA-MP-A4TD | 1 | 307 | NA | NA | 71 | MALE | Yes | T2 | 3 | 0 | 3 |
| TCGA-MP-A4TE | 1 | 896 | 1 | 845 | 56 | MALE | Yes | T2b | 1 | NA | 2 |
| TCGA-MP-A4TF | 1 | 336 | NA | NA | 58 | FEMALE | Yes | T2b | 1 | 0 | 2 |
| TCGA-MP-A4TH | 0 | 741 | 0 | 741 | 70 | FEMALE | Yes | T1a | 1 | 0 | 1 |
| TCGA-MP-A4TI | 1 | 429 | NA | NA | 72 | MALE | Yes | T2a | 2 | 0 | 2 |
| TCGA-MP-A4TJ | 1 | 339 | NA | NA | 62 | FEMALE | Yes | T1 | 1 | 0 | 1 |
| TCGA-MP-A4TK | 1 | 582 | 1 | 545 | 56 | FEMALE | Yes | T2 | 2 | NA | 2 |
| TCGA-MP-A5C7 | 0 | 2248 | 0 | 2248 | 76 | FEMALE | Yes | T2 | 1 | 0 | 1 |
| TCGA-NJ-A4YF | 0 | 2161 | 0 | 2161 | 50 | FEMALE | Yes | T1 | 1 | 0 | 1 |
| TCGA-NJ-A4YG | 0 | 2261 | 0 | 2261 | 65 | MALE | Yes | T2 | 1 | 0 | 1 |
| TCGA-NJ-A4YI | 1 | 4 | 0 | 4 | 87 | FEMALE | Yes | T2 | 3 | 0 | 3 |
| TCGA-NJ-A4YP | 0 | 50 | 0 | 50 | 52 | MALE | Yes | T2a | 1 | 0 | 1 |
| TCGA-NJ-A4YQ | 0 | 1432 | 0 | 1432 | 69 | FEMALE | Yes | T1b | 1 | 0 | 1 |
| TCGA-NJ-A55A | 0 | 15 | 0 | 15 | 76 | FEMALE | Yes | T2 | 1 | 0 | 1 |
| TCGA-NJ-A55O | 0 | 13 | 0 | 13 | 56 | FEMALE | Yes | T1b | 2 | 0 | 2 |
| TCGA-NJ-A55R | 0 | 603 | 0 | 603 | 67 | MALE | Yes | T1b | 1 | NA | 1 |
| TCGA-NJ-A7XG | 0 | 617 | 0 | 617 | 49 | MALE | Yes | T4 | 2 | 0 | 3 |
| TCGA-O1-A52J | 1 | 1798 | NA | NA | 74 | FEMALE | Yes | T1 | 1 | NA | 1 |
| TCGA-S2-AA1A | 0 | 513 | 0 | 513 | 68 | FEMALE | Yes | T1b | 1 | 0 | 1 |
