# Supplementary material for: Identification of Differentially Expressed and Prognostic lncRNAs for the Construction of ceRNA Networks in Lung Adenocarcinoma
Source: J Oncol. 2021 Dec 27;2021:2659550. doi: 10.1155/2021/2659550 (PMC8723861; doi:10.1155/2021/2659550)
Supplement: Supplementary Materials — Supplementary Figure S1. Analysis of network topology for soft thresholding powers (weighting coefficient, β) in lncRNAs, miRNAs, and mRNAs. The x-axis represented different soft thresholding powers. Upper: assessment for R2 of log(k) and log(p(k)) correlation coefficients corresponding to different β values in the network. The red line indicated a scale-free topology fitting index R2 of 0.9. Lower: analysis of the mean connectivity for various β values. Supplementary Figure S2. Correlation analysis of lncRNAs, miRNAs, and mRNAs in the ceRNA network. (a) LncRNAs were negatively correlated with miRNAs. (b) Negative correlation among mRNAs and miRNAs. (c) LncRNAs had a positive correlation with mRNAs. Supplementary Figure S3. ROC plots of the prognostic lncRNA signature in the TCGA-LUAD dataset. (a) Survival-dependent ROC curves attested the prognostic significance of DElncRNAs. The area under the red line represented the 1-year AUC; the area under the blue line represented the 3-year AUC; and the area under the black line represented the 5-year AUC. (b) ROC curve analysis showed the application value of DElncRNAs in the diagnosis. Supplementary Table S1. The summarized data of DERNAs. Supplementary Table S2. The integrated results of DERNAs via differential expression analysis, WGCNA, and website prediction. Supplementary Table S3. Baseline clinicopathological characteristics of the TCGA-LUAD cohort. (n = 513). Supplementary Table S4. Univariate Cox regression analysis of factors associated with overall survival in the TCGA-LUAD dataset. Supplementary Table S5. Multivariable Cox regression analysis of factors associated with overall survival in the TCGA-LUAD dataset. Supplementary Table S6. 6 prognostic lncRNA-mediated ceRNA networks. [file 2659550.f1.zip › 2659550.f1/Supplementary Table S5..doc]

| Supplementary Table S5. Multivariable Cox regression analysis of factors associated with overall survival in the TCGA-LUAD dataset. | | | | |
| --- | --- | --- | --- | --- |
|  | HR | 95%CI_lower | 95%CI_upper | P value |
| pT stage | 1.17 | 0.91 | 1.5 | 0.211 |
| pN stage | 0.96 | 0.64 | 1.46 | 0.865 |
| pM stage | 0.43 | 0.13 | 1.37 | 0.153 |
| pTNM stage | 1.8 | 1.13 | 2.86 | 0.013 |
| LINC01447 | 0.78 | 0.65 | 0.94 | 0.008 |
| MED4-AS1 | 0.93 | 0.77 | 1.13 | 0.477 |
| C5orf64 | 0.94 | 0.77 | 1.13 | 0.504 |
| WWC2-AS2 | 1.39 | 1.15 | 1.67 | 0.001 |
| AC133785.1 | 0.97 | 0.89 | 1.06 | 0.52 |
| OGFRP1 | 1.35 | 1.08 | 1.69 | 0.01 |
| ADAMTS9-AS2 | 0.93 | 0.81 | 1.07 | 0.288 |
| LINC00942 | 1.09 | 1.02 | 1.17 | 0.017 |
| AC090505.1 | 1.03 | 0.92 | 1.16 | 0.592 |
| LINC00887 | 0.94 | 0.85 | 1.04 | 0.231 |
| MIR99AHG | 0.96 | 0.83 | 1.11 | 0.588 |
| LINC01168 | 1.41 | 1.2 | 1.66 | <0.0001 |
| AC108676.1 | 0.94 | 0.84 | 1.06 | 0.317 |
| LNX1-AS2 | 1.1 | 0.94 | 1.27 | 0.236 |
| HOTTIP | 1.07 | 0.94 | 1.22 | 0.311 |
| AC005863.1 | 1.33 | 1.11 | 1.58 | 0.002 |
| FAM41C | 0.94 | 0.86 | 1.03 | 0.164 |
